# Genome mapping of a *LYST* mutation in corn snakes indicates that vertebrate chromatophore vesicles are lysosome-related organelles

**DOI:** 10.1073/pnas.2003724117

**Published:** 2020-10-05

**Authors:** Asier Ullate-Agote, Ingrid Burgelin, Adrien Debry, Carine Langrez, Florent Montange, Rodrigue Peraldi, Jean Daraspe, Henrik Kaessmann, Michel C. Milinkovitch, Athanasia C. Tzika

**Affiliations:** ^a^Laboratory of Artificial & Natural Evolution (LANE), Department of Genetics & Evolution, University of Geneva, CH-1211 Geneva, Switzerland;; ^b^SIB Swiss Institute of Bioinformatics, Switzerland;; ^c^Institute of Genetics and Genomics of Geneva (iGE3), University of Geneva, Geneva, Switzerland;; ^d^Faculté de Biologie et de Médecine, Electron Microscopy Facility, University of Lausanne, CH-1015 Lausanne, Switzerland;; ^e^DKFZ-ZMBH Alliance, Center for Molecular Biology of Heidelberg University (ZMBH), D-69120 Heidelberg, Germany

**Keywords:** corn snake, chromatophores, LYST, pigmentation, lysosome-related organelles

## Abstract

Reptiles exhibit a spectacular diversity of skin colors and patterns brought about by the interactions among three chromatophore types: black melanophores with melanin-packed melanosomes, red and yellow xanthophores with pteridine- and/or carotenoid-containing vesicles, and iridophores filled with light-reflecting platelets generating structural colors. Whereas the melanosome, the only color-producing endosome in mammals and birds, has been documented as a lysosome-related organelle, the maturation paths of xanthosomes and iridosomes are unknown. Here, we first use 10x Genomics linked-reads and optical mapping to assemble and annotate a nearly chromosome-quality genome of the corn snake *Pantherophis guttatus*. The assembly is 1.71 Gb long, with an N50 of 16.8 Mb and L50 of 24. Second, we perform mapping-by-sequencing analyses and identify a 3.9-Mb genomic interval where the *lavender* variant resides. The lavender color morph in corn snakes is characterized by gray, rather than red, blotches on a pink, instead of orange, background. Third, our sequencing analyses reveal a single nucleotide polymorphism introducing a premature stop codon in the lysosomal trafficking regulator gene (*LYST*) that shortens the corresponding protein by 603 amino acids and removes evolutionary-conserved domains. Fourth, we use light and transmission electron microscopy comparative analyses of wild type versus lavender corn snakes and show that the color-producing endosomes of all chromatophores are substantially affected in the *LYST* mutant. Our work provides evidence characterizing xanthosomes in xanthophores and iridosomes in iridophores as lysosome-related organelles.

## Significance

Reptiles exhibit a spectacular diversity of skin colors generated by interactions among black melanophores, red and yellow xanthophores, as well as iridophores producing structural colors. Here, we use the corn snake to investigate the generative mechanisms of skin colors beyond the zebrafish model. We perform sequencing and annotation of a nearly chromosome-quality genome of the corn snake, followed by mapping-by-sequencing and identification of a mutation in the lysosomal trafficking regulator gene (*LYST*) in the lavender variant with strongly affected coloration. Further analyses indicate that color-producing organelles of all chromatophores are substantially impacted in the *LYST* mutant, indicating that not only melanosomes, but also xanthosomes and iridosomes, are all lysosome-related organelles.

Classical model species represent remarkable resources to efficiently investigate the mechanisms producing a wealth of phenotypes and to develop tools and techniques that can be applied to emerging models. The zebrafish (*Danio rerio*) is the main nonmammalian vertebrate model of skin coloration, and its study has provided important insights into the development and organization of chromatophore cells, as well as their interactions that bring about its pattern of yellow and black stripes (1, 2). The dark stripes of the adult zebrafish consist of the superimposed layers of three chromatophore cell types in the skin: the melanin-producing and light-absorbing melanophores at the bottom of the dermis, the light-reflecting iridophores containing guanine crystals in the middle, and xanthoblasts, i.e., unpigmented xanthophores, in the top layer (3). In the interstripe regions, melanophores are absent, yellow-pigmented xanthophores are compact and densely packed, and iridophores are cuboidal and epithelium-like organized (4). The numerous spontaneously occurring or mutagenesis-induced skin color and color-pattern phenotypes in zebrafish, along with its established position as a model species for developmental studies, facilitated the identification of the signaling pathways and cell interactions involved in vertebrate skin color and color patterning. This knowledge provides the basis for the study of the striking diversity of skin color and color patterns in other vertebrates, such as squamate reptiles.

In zebrafish, direct observation of chromatophores is facilitated by the transparent and translucent skin of larvae and adults, respectively, so densities of melanophores and xanthophores can be readily quantified in various color- and pattern-affecting genetic variants (5). Yet, limited information is available on the effect of these mutations at the chromatophore subcellular level. The analysis of the wild type zebrafish revealed that 1) melanophores contain uniform (∼0.5 μm in diameter) and densely packed melanosomes, 2) iridophores hold uniformly spaced guanine crystals, and 3) xanthophores can carry both pteridine-containing xanthosomes and carotenoid-containing vesicles (3). In vertebrates, xanthophores and erythrophores contain organelles with yellow and red pigments, respectively, but these are not distinguishable in TEM imaging and are therefore often interchangeably referred to as xanthophores. Note also that, in lizards of the genus *Phelsuma*, the in vivo red color of erythrophores and yellow color of xanthophores are likely generated by identical pigments subjected to different endosomal pH or pigment redox states (6). We thus use the term xanthophores to refer to both red- and yellow-pigmented cells.

The only chromatophore present in mammals and birds is the melanophore. Extensive literature exists on the migration, maturation, and differentiation of melanophores from neural crest cells (7–9). It is well documented that melanosomes are lysosome-related organelles (LROs), which start as nonpigmented vacuolar early endosomes in stage I of their biogenesis, acquire internal striations in stage II, and progressively accumulate melanin in stage III, until they are fully melanized in stage IV (10). Genetic studies on naturally occurring human and mouse color phenotypes presenting hypopigmentation and amelanism identified genes exhibiting key functions in neural crest cells development and survival (Waardenburg syndrome; e.g., ref. 11), melanosome biogenesis/maturation and transport (Hermansky–Pudlak syndrome; e.g., ref. 12), and regulation of pigment synthesis or melanosome function (albinism; e.g., ref. 13).

Less information is available on the maturation of xanthosomes in xanthophores and iridosomes in iridophores. In the axolotl (*Ambystoma mexicanum*), the presence or absence of fibrous material in xanthosomes seems to be related to their pigment composition rather than their stage of differentiation (14). In the adult African clawed frog (*Xenopus laevis*), most xanthosomes display well-developed concentric lamellar structures on which pigments are probably deposited, whereas others appear empty or contain fibrous material (15). Genes involved in iridophore differentiation and migration have mainly been identified by genetic studies of zebrafish mutants (16), but very little is known about the mechanism of guanine crystal formation. An organellogenesis study of cultured *Xenopus* iridophores showed that spherical vesicles accumulate electron-dense material before they take the shape of the enclosed crystals that eventually fill them up, but the origin of these vesicles has not been elucidated yet (17). Bagnara et al. (18) discussed the possible common origin of color-producing organelles, based on observations of chromatophores simultaneously carrying more than one type of these organelles in several species, snakes included. Given that mutations in *LYST* and *HPS* in humans and mice affect the biogenesis of melanosomes, the latter have been labeled as “lysosome-related organelles” (as summarized in ref. 19). On the contrary, no study focused on the origin and the characterization of xanthosomes and iridosomes (i.e., compartments containing reflecting guanine platelets).

Reptiles exhibit a very large diversity of skin color and color pattern phenotypes that have been scarcely studied. Previous research in snakes and lizards principally aimed at describing the so-called chromatophore unit (i.e., the positional relations of chromatophores in the skin), as well as identifying pigments and the genes involved in their metabolism (20–23). Surprising dynamics of ontogenic color-pattern development, such as the cellular automaton-guided color-flipping scales of the ocellated lizard (24), indicate the remarkable efficiency of the reaction-diffusion system [as described for the zebrafish (5)] in modeling skin color pattern formation in squamates. Yet, little is known of the underlying interactions among chromatophore cells during squamate development.

**Fig. 1.**
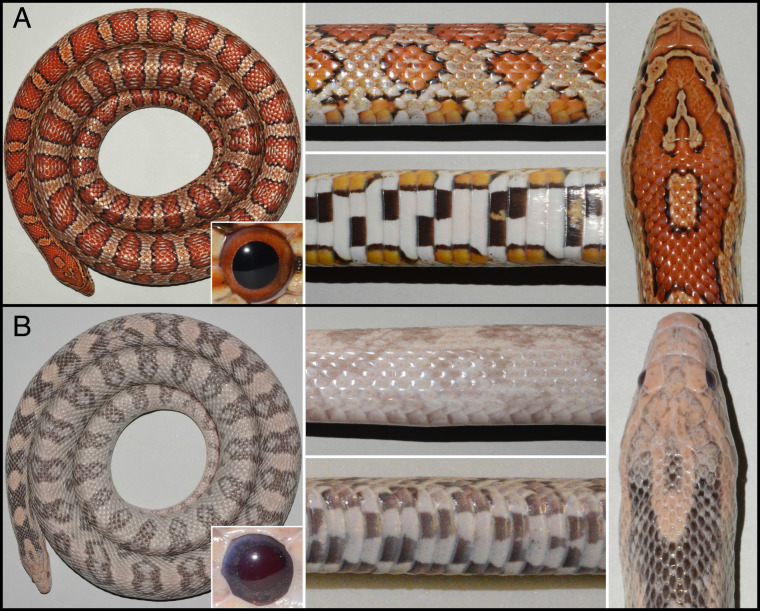
Coloration of (*A*) wild type and (*B*) lavender corn snakes. A dorsal overview (*Left*), close-ups of lateral and ventral views (*Center*), and a head dorsal view (*Right*) are provided. The insets show the iris coloration. The coloration of lavender individuals is heavily affected, but no obvious change in the color pattern is detectable.

To bridge this gap, we propose the corn snake (*Pantherophis guttatus*) as a model for color and color pattern studies in reptiles (25–27) because of 1) the ease of its maintenance and breeding in a laboratory setting; 2) its long lifespan in captivity; 3) its gentle and nonvenomous nature; 4) its relative prolificacy, with 20 to 30 eggs laid per female and per year; and 5) the availability of numerous spontaneously occurring color and color pattern morphs (26). Nevertheless, due to the constraints of long generation time (3 to 4 y) and seasonal breeding, genetic analyses in corn snakes necessitate collecting data for several years. The wild type corn snake is characterized by red dorsal and lateral blotches on an orange background, as well as a ventral checkerboard-like black and white pattern (Fig. 1*A*). Various morphs differ from that phenotype by exhibiting altered colors or a modified pattern. Mapping-by-sequencing analyses (28) can determine a genomic interval within which the causative variant of a phenotype lays. In the event that the causal gene is identified by analyzing candidate genes positioned in the genomic interval, it becomes possible to investigate how the development and function of chromatophore(s) and/or the patterning process are/is altered. Such an approach revealed that amelanism in the corn snake is associated with the insertion of an LTR-retrotransposon in the oculocutaneous albinism II (*OCA2*) gene (25). The corresponding OCA2 protein, localized on the membrane of melanosomes, likely controls the melanosome’s acidic pH required for proper tyrosinase activity (29–33). In parallel, availability of the first draft of the corn snake genome (27) showed that mapping by sequencing is greatly facilitated when based on genomic, rather than transcriptomic (exome), data.

Here, using 10x Genomics linked-reads, which provide long-range information, and optical mapping to correct for misassemblies, we assembled and annotated a nearly chromosome-quality corn snake genome. Its high contiguity greatly facilitated our mapping-by-sequencing analyses to identify the 3.9-Mb interval where the *lavender* variant resides. Lavender individuals are characterized by gray, rather than red, blotches on a pink, instead of orange, background (Fig. 1*B*). The skin color pattern is not noticeably affected in the lavender corn snake phenotype, and we thus consider it a color variant. Our genomic and transcriptomic sequencing analyses reveal a single nucleotide polymorphism introducing a premature stop codon in the coding sequence of one of the main candidate genes in the interval: the lysosomal trafficking regulator (*LYST*). *LYST* is involved in endolysosomal biogenesis, and defects in its coding region can result in enlarged lysosome-related organelles, including melanosomes, such as in the beige (34) and gray (35) phenotypes in mice, the Aleutian coloration in minks (36), and the Chédiak–Higashi syndrome in humans (37). Second, using transmission electron microscopy and histology, we provide an extensive comparative description of the chromatophore unit in wild type vs. *LYST* mutant corn snakes. These analyses reveal that the color-producing endosomes, i.e., melanosomes, xanthosomes, and iridosomes, are substantially affected in the *LYST* mutant, producing the lavender phenotype (Fig. 1*B*). Our work provides evidence characterizing xanthosomes in xanthophores and iridosomes in iridophores as lysosome-related organelles, as had previously been done for melanosomes in melanophores.

## Results

### The Corn Snake Genome.

By comparing the DNA content in corn snake and chicken nucleated erythrocytes using fluorescence-activated cell sorting (FACS), we estimated the corn snake genome size to about 1.68 Gb (*SI Appendix*, Fig. S1), in accordance with the 1.74 to 1.79 Gb value obtained from our draft assembly on the basis of *k*-mer distribution (27). The corn snake karyotype consists of 18 pairs of chromosomes (*SI Appendix*, Fig. S2), of which 10 are microchromosomes (38). To obtain a high-quality genome assembly, we sequenced 425 million paired-end Illumina reads from a 10x Chromium library of an adult male corn snake (male being the homogametic sex) and assembled them with Supernova (39). We combined the Supernova assembly with 3,190 Bionano optical genome maps to correct for misassemblies. The final corn snake genome assembly (GenBank accession number GCA_001185365.2) is 1.71 Gb long, consists of 34,268 sequences >1 kb, and is of high integrity with only 4.7% of gaps. The N50 is 16.8 Mb, with 50% of the genome found in only 24 sequences (L50). Hence, this approach produced an assembly that is considerably more complete and contiguous than the published corn snake draft genomes (27, 40) (*SI Appendix*, Table S1) and represents one of the best snake genomes published to date (Table 1).

**Table 1.** Statistics for the final corn snake genome assembly compared to other published snake genomes

Species	Assembly length in Gb (% of gaps)	Number of sequences	N50, kb	L50
*P. guttatus* (this article)	1.71 (4.7%)	34,268	16,790	24
*Boa constrictor*	1.44 (3.9%)	19,927	4,505	90
*Python bivittatus*	1.44 (3.5%)	39,113	214	1,939
*Deinagkistrodon acutus*	1.51 (5.9%)	162,571	2,075	199
*Pseudonaja textilis*	1.59 (2.5%)	28,550	14,686	31
*Notechis scutatus*	1.67 (4.8%)	52,414	5,997	66
*Crotalus viridis*	1.34 (6.2%)	7,043	103,291	3

**Fig. 2.**
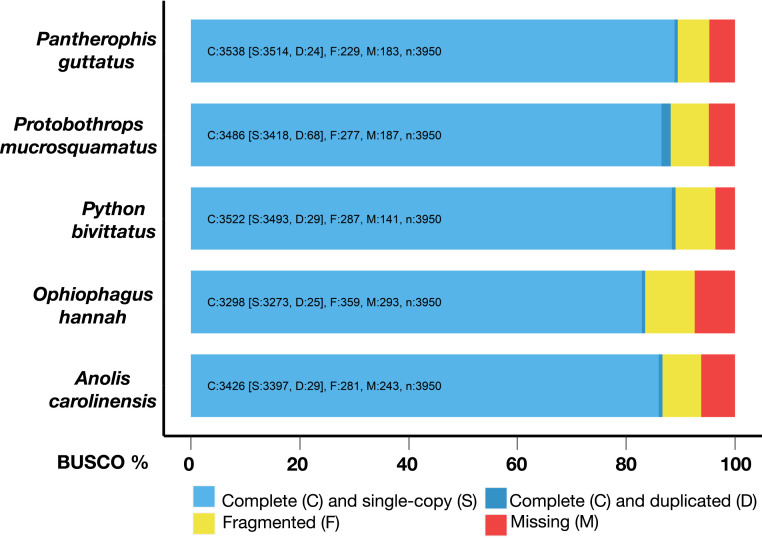
BUSCO results for the corn snake genome sequenced and assembled here as well as for four previously published squamate genomes. The genome completeness assessment was performed using the 3,950 single-copy genes of the Tetrapoda set.

To assess genome completeness, we verified the presence of the 3,950 single-copy genes of the BUSCO Tetrapoda set. We fully retrieved 3,538 of them (89.57%), with only 183 missing (4.63%; Fig. 2 and *SI Appendix*, Fig. S3). The presence and correct ordering of each *HOX* gene cluster in a single scaffold together with the corresponding *DLX* gene pairs (at distances up to 10 Mb, depending on the cluster) illustrates the excellent continuity of our corn snake assembly (*SI Appendix*, Fig. S4). The RepeatModeler library for the corn snake consists of 1,469 repeat families, of which 673 are classified. The repetitive elements accounted for 44.17% of the genome assembly (*SI Appendix*, Table S2 and Fig. S5), presenting a diverse landscape of repetitive elements, as in other squamates (41–44). In the previously published draft version of the corn snake genome, twice as many repeat families were identified; however, repeats were much shorter and fragmented (mean length 315 bp vs. 521 bp in the current assembly). In the nearly chromosome-quality version presented here, we identify a high prevalence of long interspersed elements (16.99% of the genome, L2 and CR1 being the most abundant) and DNA transposons (9.34%). Finally, 4.43% are long terminal repeat (LTR) retrotransposons, of which 0.14% correspond to the repetitive element responsible for the amelanistic phenotype (25).

### Corn Snake Genome Annotation.

The genome annotation analysis with BRAKER predicted 27,642 genes and 32,398 isoforms, whereas the MAKER pipeline modeled 26,182 genes. In both cases, we only retain gene models that either have a Pfam protein domain or are supported by transcriptomic or proteomic data, that is, with an Annotation Edit Distance less than 1 (AED < 1; *SI Appendix*, Fig. S6*A*). The annotation of 25,771 proteins in BRAKER and 21,183 in MAKER is supported by other Squamata or UniProtKB proteins (*SI Appendix*, Fig. S6*B*), with, respectively, 13,792 and 10,443 being fully retrieved. The two annotation datasets complement each other, as some genes retrieved by MAKER are not found by BRAKER and vice versa. Therefore, keeping both annotations decreases the risk of missing the possible causative variant for the lavender phenotype. The genome assembly and its annotation can be publicly accessed via a genome browser (https://www.reptilomics.org/jbrowse/Pantherophis_guttatus).

### Retrieval of the Causative Variant for the Lavender Phenotype.

**Fig. 3.**
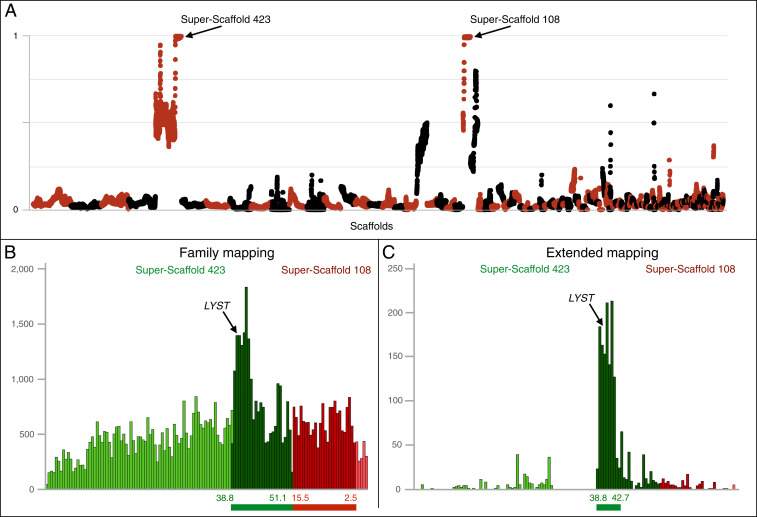
Mapping the *lavender* variant. (*A*) Proportion (*y* axis) of quality-filtered SNP/MNP cosegregating with the *lavender* locus in the four genome libraries compared to informative quality-filtered parental variants (homozygous in the *lavender*/*lavender* father and heterozygous in the *lavender*/^+^ female). Proportions are calculated for scaffolds >1 Mb, with a 1-Mb sliding window and a step of 100 kb. Scaffolds (alternatingly colored black and red) are ordered from longest to shortest, and the two superscaffolds containing the *lavender* interval are indicated. A close-up of these superscaffolds is provided in *SI Appendix*, Fig. S7. (*B*) Number of biallelic variants (SNP/MNP and indels) in 500-kb intervals cosegregating with the *lavender* locus in superscaffolds 423 and 108 based on the family mapping. (*C*) Number of biallelic variants (SNP/MNP and indels) in 500-kb intervals cosegregating with the *lavender* locus in superscaffolds 423 and 108 based on the extended mapping. (*B* and *C*) Scaffolds are ordered and oriented based on their synteny to the *G. gallus* and *A. carolinensis* genomes. Dark green and dark red bins correspond to the 25.3-Mb region with the highest proportion of cosegregating variants in the family mapping. The length/position of the two intervals is shown with a thick line under each graph, and an arrow points to the position of *LYST.*

Crosses in our corn snake colony indicate that the lavender phenotype is caused by a recessive single-locus variant. Using the newly assembled near chromosome-quality corn snake genome presented above and deep sequencing of homozygous and heterozygous lavender corn snakes generated by the same pair of parents, we performed a variant-calling analysis to locate the locus responsible for the lavender morph. More specifically, we sequenced separately four genomic DNA libraries: the wild type mother (*lavender*/^+^), the lavender father (*lavender*/*lavender*), and two pools of *lavender*/*lavender* and *lavender*/^+^ F1 offspring (these two genotypes are readily distinguished by their corresponding lavender vs. wild type phenotypes). We aligned each library to the newly assembled genome and searched for nucleotide polymorphisms (SNPs and MNPs in nonrepetitive elements) cosegregating with the lavender phenotype. This approach identified an interval at the extremities of two long scaffolds (superscaffolds 423 and 108) with a total length of 25.3 Mb (Fig. 3 *A* and *B* and *SI Appendix*, Figs. S7 and S8*A*). It includes 32,088 cosegregating SNPs/MNPs with an average density of 1.26 variants/kb. The interval is syntenic to chromosome 1 (between 180 and 182 Mb and 202 and 225 Mb) and five unplaced scaffolds (GL343259.1, GL343200.1, GL343315.1, GL343358.1, and GL343681.1) of the *Anolis carolinensis* v2 genome. In the *Gallus gallus* v6 genome, the interval corresponds to chromosome 3 from 37.3 to 61.3 Mb. We genotyped 49 snakes of the lavender family for cosegregating SNPs and identified 4 recombinants, thus reducing the interval to 17.2 Mb (*SI Appendix*, Fig. S9).

To further reduce the interval, we performed an analysis, here referred to as extended mapping, combining family mapping with the sequencing of unrelated individuals. To this end, we run the variant calling as described above and included, besides the four family libraries, five additional libraries of unrelated individuals, one of which is *lavender/+* and the other four are *+/+*. This analysis identified 1,869 biallelic cosegregating variants (including indels) in superscaffolds 423 and 108, of which 1,248 are located in a 3.9-Mb interval at the distal end of the superscaffold 423 (Fig. 3*C* and *SI Appendix*, Fig. S8*B*). This interval corresponds to chromosome 3 from 37.3 to 40.8 Mb in *G. gallus* and to chromosome 1 from 218.06 to 223.2 Mb in *A. carolinensis*. Our annotation pipeline and the NCBI RefSeq annotation of our assembly (National Center for Biotechnology Information *P. guttatus* Annotation Release 100) identified 267 genes in the original 25.3-Mb genome interval of the family mapping and only 54 genes in the much reduced 3.9-Mb interval obtained with extended mapping. The corresponding interval contains 55 genes in *G. gallus* and 54 genes in *A. carolinensis* and *Pseudonaja textilis*, another snake species. The synteny differences compared to these species are 1) the IFN regulatory factor 2 binding protein 2 (*IRF2BP2*) is missing in *A. carolinensis* and 2) the geranylgeranyl diphosphate synthase 1 (*GGPS1*) is located on a short corn snake scaffold and is absent from the *P. textilis* genome (*SI Appendix*, Table S3).

To further narrow down the list of candidate genes, we looked for variants (polymorphisms or indels) that both cosegregate with the lavender phenotype and affect the protein sequence of any of the 55 genes in the 3.9-Mb corn snake interval (accounting for *GGPS1*). Most polymorphisms are within introns or in intergenic intervals, and we identified single- and multinucleotide polymorphisms within the coding sequence of 10 genes resulting in amino acid polymorphisms. Only for the *LYST* transcript, as modeled by MAKER, we detected a much more disruptive mutation: a single nucleotide polymorphism (C to T) at position 9,704 of exon 41, corresponding to position 9,508 in the CDS, introducing a stop codon instead of a glutamine (Q). Hence, the translated protein is shortened by 603 amino acids (from 3,772 to 3,169), the evolutionary-conserved BEACH domain is very substantially truncated, and the four WD40 domains are missing (*SI Appendix*, Fig. S10). The length of the protein product of the corn snake lavender variant is similar to that identified in Aleutian minks, where a frameshift at position 9,468 generates a premature STOP codon, shortening the LYST protein from 3,801 to 3,193 amino acids (36). Minks carrying this variant have a gun-metal gray pigmentation instead of brown. No other frameshift or premature STOP codon was identified in the other 54 genes in the interval (*SI Appendix*, Table S3). Hence, although we cannot exclude the involvement of other polymorphisms in the lavender phenotype, the premature STOP codon introduced in the *LYST* transcript is the strongest candidate responsible for this phenotype.

We genotyped, by sequencing genomic DNA, the SNP in *LYST* in 1) 52 individuals of the lavender lineage in our colony (*SI Appendix*, Table S4) and 2) 71 individuals belonging to 35 unrelated lineages (*SI Appendix*, Table S5). The resulting genotypes are compatible with the phenotypes observed in all individuals. Note that, besides the individuals used for the family mapping, we have access to three additional lavender lineages carrying the SNP in *LYST*. A *lavender/+* snake from one of these lineages (EG339), whose genotype was verified by crosses with lavender individuals, was sequenced for the extended mapping as detailed above. The lavender variant transcript of the *LYST* mRNA is unlikely to be substantially decayed, as we easily sequenced it in three lavender individuals and one heterozygous individual (*SI Appendix*: *LYST* sequence).

### The Corn Snake Chromatophore Unit.

**Fig. 4.**
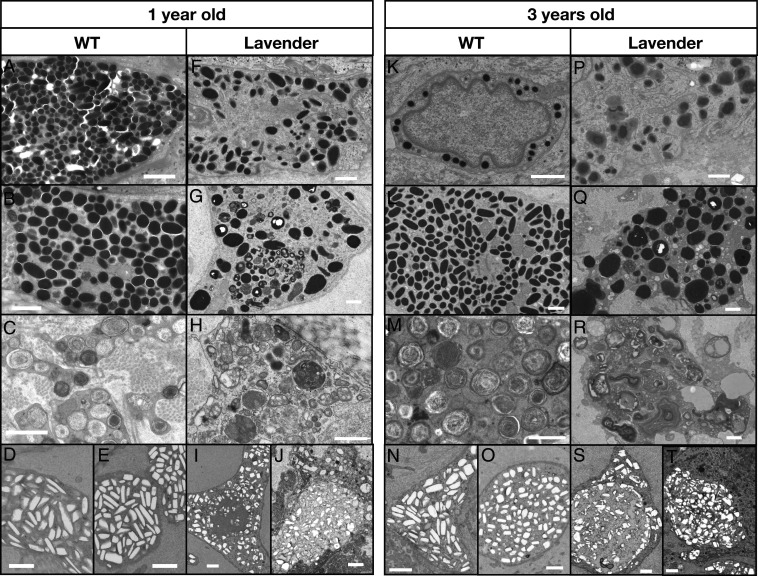
Wild type and lavender corn snake chromatophores of 1- and 3-year-old animals. In the wild type: (*A* and *K*) epidermal melanocytes, (*B* and *L*) dermal melanocytes with ellipsoidal mature melanosomes (a few are at earlier stages of maturation at 1 y old), (*C* and *M*) xanthophores with xanthosomes containing concentric lamellae (at 1 y old, some xanthosomes are at earlier stages of maturation and contain amorphous material), and (*D* and *N*) ventral and (*E* and *O*) dorsal iridophores carrying crystals of guanine. In the lavender: (*F* and *P*) epidermal melanosomes are larger than in the wild type and have irregular shapes; (*G* and *Q*) dermal melanosomes are larger and more irregularly shaped than in the wild type and mature more slowly, as several are not fully melanized after 1 y, and become bigger and irregularly shaped; (*H* and *R*) the amorphous material of the xanthosomes accumulates more slowly and forms irregularly shaped lamellae in the lavender instead of concentric ones as in the wild type; and (*I* and *S*) ventral and (*J* and *T*) dorsal guanine crystals in iridophores are more irregularly shaped and might accumulate in larger compartments than in the wild type. (Scale bars, 1 μm.)

Wild type corn snakes carry red dorsal blotches on an orange background and a black and white checkered pattern on their ventral side (Fig. 1*A*). We collected skin samples from four distinct regions: the blotches and background skin on the dorsum, as well as the black and white part of the ventral scales. We analyzed these samples with light microscopy and transmission electron microscopy (TEM) to identify chromatophores, their spatial organization, and the morphology of their vesicles containing pigments or guanine crystals. Note that we confirmed by Raman spectroscopy that the crystals are indeed made of guanine (*SI Appendix*, Fig. S11). In the orange background skin, a tightly packed layer of xanthophores is located just under the epidermis, large iridophores and a few xanthophores populate the loose dermis underneath, and a handful of expanded melanophores occur near the subcutis (*SI Appendix*, Figs. S12*A* and S13*A*). Even though the melanophores of the corn snake exhibit a branched morphology, cell projections do not seem to reach the top layers of the dermis. This chromatophore combination results in a light orange color. The red skin of dorsal blotches exhibits the same organization as the orange skin of the background except that the former lacks iridophores. Note that epidermal melanophores are observed all across the dorsal skin (Fig. 4*A*), with greater density along the dorsal midline and a monotonously decreasing density on the flanks toward the belly (*SI Appendix*, Fig. S14*A*). The ventral scales of the corn snakes are large, covering the ventrum from one side to the other, and they are white with black patches (Fig. 1*A*). In the dermis of the white portion of ventral scales, we only observe iridophores, and, in the black portion, there are only melanophores. In ventral scales, epidermal melanophores are scarce (*SI Appendix*, Fig. S14).

Melanosomes in the dermal melanophores are fully mature and ellipsoidal with a long axis of up to 1 μm (Fig. 4 *B* and *L*). Xanthosomes have a maximum diameter of 0.8 μm and are filled with concentric lamella (Fig. 4 *C* and *M*). The iridosomes of the ventral (Fig. 4 *D* and *N*) and dorsal (Fig. 4 *E* and *O*) skin iridophores are heterogeneous in size and in their spatial distribution. As such, disorganized guanine crystals are likely to form a broad-band reflector that lightens up skin color (6); their absence in the dorsal blotches might contribute, together with potentially different pigment contents of xanthophores, to the corresponding darker coloration. Note that, for iridophores and xanthophores, the guanine crystals or the lamellae are often lost during TEM sample preparation, with only the enveloping membrane being observed. In the samples and sections we observed, we did not identify any carotenoid vesicles.

### Lavender Corn Snakes.

Lavender individuals present gray, rather than red, dorsal blotches on a pink, rather than orange, background. In addition, the black ventral checkers appear more faded than in the wild type (Fig. 1*B*). The color of the iris also changes from orange/red in the wild type to gray in the lavender (Fig. 1). Note that lavender corn snakes in our colony present variable intensities of pink and black (*SI Appendix*, Fig. S15). Imaging of animals over a period of 24 mo since hatching indicates that the vivid pink coloration, very visible on the background skin (and somewhat in the dorsal blotches) at the beginning of their life, slowly fades over time, and the black outlines of blotches turn to gray (*SI Appendix*, Fig. S16*A*). In wild type individuals, the orange background becomes more intense and the dark red of the dorsal blotches turns to orange as the animal grows (*SI Appendix*, Fig. S16*B*).

Observation of deparaffinized microtome sections shows that all types of visibly pigmented chromatophores are greatly reduced in lavender snakes, in particular the number of pigmented xanthophores, which are very scarce and difficult to locate (*SI Appendix*, Fig. S12*B*). Note that the chromatophores might still be present, but they might have lost their pigments/crystals, so we cannot identify them. A zebrafish *lyst* mutant has been produced with ENU mutagenesis (*7653*^*mu107*^ [45]). The authors mention that the *lyst* mutant showed a pale skin tone; inspection of figures 2 and 3 in ref. 45 suggests a reduced yellow pigmentation in the larvae, but no further details are provided.

Our TEM imaging uncovers a spectacular modification of the subcellular content of all lavender chromatophores (Fig. 4 *F–J* and *P–T* and *SI Appendix*, Fig. S13*B*). Lavender dermal and epidermal melanophores, instead of containing mature melanosomes as in the wild type, contain enlarged nonellipsoidal melanosomes at different stages of maturation (Fig. 4 *P* and *Q* vs. Fig. 4 *K* and *L*), while the rare xanthophores contain xanthosomes that fail to form the concentric lamellae observed in the wild type (Fig. 4*R* vs. Fig. 4*M*). In lavender iridophores, crystals seem grouped within larger compartments and exhibit shapes that are less parallelepipedal than those in wild type iridophores (Fig. 4 *S* and *T* vs. Fig. 4 *N* and *O*). Our measurements of crystal roundness and solidity (i.e., parameters that estimate how circular and how convex an object is, respectively) show that lavender crystals differ in shape from their wild type counterparts, especially when comparing between the two morphs the solidity of the dorsal crystals and the roundness of the ventral ones (*SI Appendix*, Fig. S17). All these vacuolar phenotypes are accentuated with the age of the animal (Fig. 4 *F–J* and *P–T*). These subcellular modifications discovered in all chromatophores of the lavender morph indicate that *LYST* is a key gene for the proper development not only of melanosomes, but also of xanthosomes and iridosomes.

### Lavender LROs Are Enlarged Compared to the Wild Type.

**Fig. 5.**
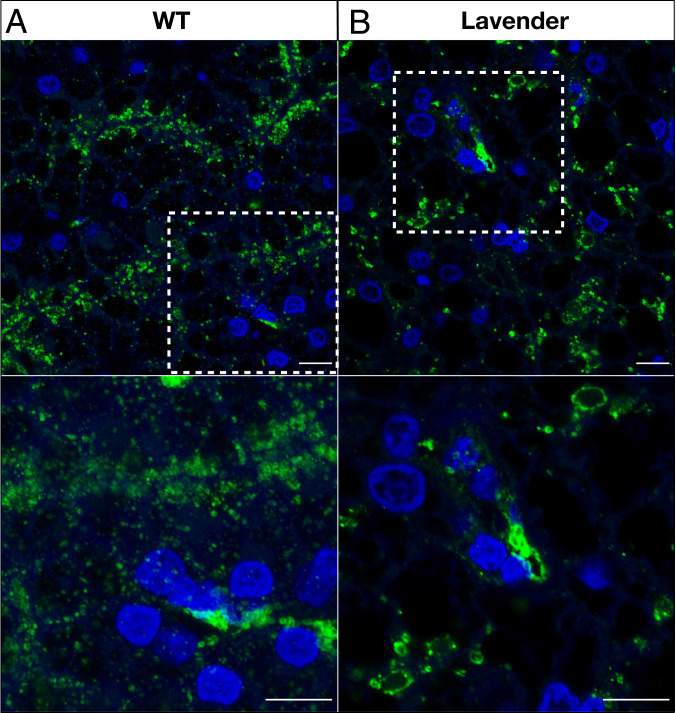
LAMP1 immunostaining of liver cells. (*A*) Lysosomes of the wild type corn snake are small and widely distributed in the hepatocytes, whereas (*B*) lysosomes of the lavender corn snake aggregate and form bigger structures. Dashed squares include the regions shown in greater magnification below. (Green: LAMP1, blue: nuclear DAPI staining; scale bars, 10 μm.)

LYST is involved in the trafficking of lysosomes and lysosome-related organelles (LROs), such as melanosomes, as well as in controlling the size and number of these vesicles (46, 47). In mouse and minks, *LYST* variants affect the biogenesis of melanosomes (35, 36). In addition, mutations affecting *LYST* in humans cause the Chédiak–Higashi syndrome, characterized by oculocutaneous albinism and immunodeficiency (37, 48, 49). It has been suggested that LYST-deficient cells accumulate enlarged LROs because endosomal vesicles gradually assemble and fuse (46). As hepatocytes contain a large number of lysosomes, we performed immunohistochemistry on liver paraffin sections of multiple wild type and lavender corn snakes using a primary antibody against LAMP1 (Lysosomal-associated membrane protein 1) as a lysosomal marker. In the wild type cells, we observe a great number of small lysosomes (Fig. 5*A* and *SI Appendix*, Fig. S18), whereas, in the lavender hepatocytes, lysosomes are larger and form aggregates (Fig. 5*B* and *SI Appendix*, Fig. S18), in agreement with the published phenotype of *LYST* mutants in mouse and human (47, 50, 51).

## Discussion

Previous analyses of reptilian coloration mainly focused on the phenotypic diversity encountered in wild populations of lizards (20, 22, 23) and snakes (21). A recent study on *Ctenophorus decresii*, an agamid lizard, has identified that variations in the pteridine and carotenoid content of xanthophores explain the differences of throat coloration between two genetically distinct lineages, ranging from gray to yellow and orange, and that differentially expressed genes account for these variations (20). Similarly, for the common wall lizard *Podarcis muralis*, high-resolution mapping revealed that coloration differences among populations are due to regulatory variants in key enzymes of the pteridine and carotenoid metabolism (23). We promote the corn snake as a model species to study the development of reptilian coloration because it is easy to maintain and breed in captivity, and numerous color and color pattern morphs exist and are associated to monolocus mutations with Mendelian inheritance. Mapping these variants to a high-quality genome, combined with detailed characterization of the developing pigment cells and the skin, should facilitate extensive study of the development of reptilian chromatophores, their interactions, and their pigment/crystal-producing function in a systematic manner.

Here, by performing mapping-by-sequencing analyses, we identify a 3.9-Mb interval bearing the causative mutation for the lavender morph. We show that a premature stop codon in the lysosomal trafficking regulator *LYST* is likely responsible for the pink coloration with gray blotches of the lavender corn snakes. Our transmission electron microscopy imaging uncovers that the subcellular structure of all chromatophores is affected in these individuals. Lavender melanosomes are enlarged and less ellipsoidal than in the wild type, resembling the melanosomes in the retinal pigment epithelium of the *Lyst*-mutated beige mice (34). Pigmented xanthophores are very scarce in lavender, and their xanthosomes are enlarged and lack the concentric lamellae found in the wild type. We cannot differentiate whether the number of xanthophores is very much reduced or if xanthophores are present in normal numbers but exhibit a greatly reduced ability to produce and/or accumulate pigmented organelles. Guanine crystals in lavender iridophores are strongly deformed, being more irregular and less parallelepipedal, and seem to group in larger cellular compartments. We also observe enlarged lysosomes in the lavender hepatocytes, corroborating the presence of giant organelles in various cell types, as observed in human patients affected by the Chédiak–Higashi syndrome (51). Given that *LYST* mutations strongly affect multiple lysosome-related organelles (LROs), it is likely that this regulator plays a role in the biogenesis of all these cellular compartments from the endolysosomal system (52). Note that a previous study, based on the occasional presence of melanosomes, xanthosomes, and/or guanine crystals in a single cell, already hinted at the potential common origin of all these endosomes from a primordial organelle, which originated from the endoplasmic reticulum (18). Here we provide evidence that characterize xanthosomes and iridosomes as lysosome-related organelles, underlying the insightful suggestion of Bagnara et al. (18) based on cellular morphologies. Our finding suggests that xanthosomes and iridosomes are thus likely to originate from maturing endosomes, as melanosomes do (52).

In addition to reduced pigment in the skin and eyes, the Chédiak–Higashi syndrome in humans is characterized by immune deficiency, bleeding, and bruising at variable levels (52). Besides their modified coloration, lavender corn snakes do not present any other perceivable phenotype, as they feed, grow, and breed normally in captivity. Note that this is also the case for Aleutian minks, which do not display any visible clinical abnormalities (36) even though their neutrophils display giant granules. It has also been suggested that a *lyst* variant is responsible for the reduced melanin pigmentation in the *brass* (or *golden-2*) zebrafish strain (53–55). The *7653^mu107^ lyst* zebrafish mutants (45) carry a mutation that shortens lyst from 2,666 aa to only 336 aa. Thus, the length reduction of the protein is greater than in the lavender LYST. Besides pale-yellow coloration, the *7653^mu107^ lyst* zebrafish mutants show hepatomegaly, liver steatosis, and undetermined kidney defects. Evaluating the impact of the *LYST* mutation on the lavender liver warrants further investigation but will be greatly hindered by the peculiar metabolism of this organ in snakes: the mass and function of several organs, especially the liver, can be greatly affected in snakes upon feeding and during digestion (56, 57). This is confirmed by our analyses showing great variation in liver size and lipid content among individuals independently of their wild type or lavender phenotype. On the contrary, it is remarkable that, despite these variations among the five individuals we investigated, all three lavender and neither of the two wild type exhibit enlarged lysosomes (*SI Appendix*, Fig. S18). These results further support our hypothesis that the *LYST* mutation generates the lavender phenotype as LYST-deficient cells accumulate enlarged LROs (46, 47, 50, 51). A transgenic corn snake carrying the identified SNP in *LYST* would provide the definite proof that this SNP is responsible for the lavender phenotype.

Another important point that remains to be clarified is how a deficient LYST impacts on the development of chromatophores. We suggest three alternative hypotheses. First, the number of chromatophores, xanthophores in particular, could be reduced from the onset of skin development, which would imply that color patterning in snakes and zebrafish differs substantially. Indeed, in zebrafish, it has been shown that the three chromatophore types interact extensively during development and metamorphosis to generate the adult yellow and black stripes; the positions and densities of each chromatophore type, as well as the timing of their interactions, heavily impact on the skin color patterning process (1, 58). Second, the precursors of all chromatophores might differentiate properly until the wild type distribution of the chromatophores (corresponding to the color pattern that will form after pigments have been produced) is established but degenerate as the animals grow, contributing to the fainter lavender coloration. Third, normal densities and distribution of chromatophores might be in place during development and adulthood in lavender corn snakes, but chromatophores might fail to produce a pigmented phenotype. Identifying the correct hypothesis will provide an improved understanding of the plasticity of color and color pattern formation in vertebrate skin.

## Materials and Methods

### Animals.

Corn snakes were housed and bred at the LANE animal facility running under veterinary cantonal permit no. 1008. The individuals were sampled and imaged following Swiss law regulations and under experimentation permit GE/169/17.

### Corn Snake Genome Size.

Blood was collected from a male corn snake and a chicken; 0.5 mL of blood was mixed with 5 mL of 0.7 mM EDTA in HBSS (no. 14175095; Gibco) and stored at 4 °C. The cells were washed with 2% calf serum (no. 12238C; Sigma-Aldrich) in DPBS (D8537; Sigma-Aldrich), resuspended in 1 mL of propidium iodide solution (20 μg/mL PI; no. 81845; Sigma-Aldrich) with 100 μg/mL RNase A in DPBS–2% calf serum, and incubated at 37 °C for 30 min. The samples were stored at 4 °C and analyzed the same day on a BD Accuri C6 flow cytometer.

### Corn Snake Karyotype.

Chromosome spreads were prepared from corn snake fibroblasts established from an E30 embryo. Fibroblasts were grown at 30 °C in MEM (M5650; Sigma-Aldrich) supplemented with 2 mM L-glutamine (no. 25030024; Gibco), 10% calf serum (C8056; Sigma-Aldrich), 100 U/mL penicillin/streptomycin (no. 15140122; Gibco), 2.5 μg/mL amphotericin B (A2942; Sigma-Aldrich), and 50 μg/mL gentamicin (no. 15750037; Gibco). KaryoMAX Colcemid Solution in PBS (no. 15212012; Gibco) was used at 0.1 μg/mL to arrest cells in metaphase. Cells were collected after trypsin digestion and incubated for 10 min at 37 °C in a 0.075 M KCl solution. They were fixed in methanol:acetic acid (3:1), and the cell suspensions were dropped onto clean glass slides and air-dried. Chromosomes were stained with KaryoMAX Giemsa Stain Solution (no. 10092013; Gibco), rinsed with distilled water, air-dried, and mounted with Ultrakitt (no. 3921; JT Baker).

### Whole-Genome Sequencing and Optical Mapping.

High molecular weight (HMW) genomic DNA (greater than 50 kb) was extracted with the Qiagen Genomic-tip 20/G kit (no. 10223; Qiagen) from nucleated erythrocytes of an adult male individual. These long molecules were used for the 10x Chromium library preparation (Novogen) with the Chromium Genome HT Library Kit and Gel Bead Kit v2 (59). We sequenced the resulting barcoded fragments with a HiSeq X instrument, obtaining 425 million 150-bp paired-end reads. We also isolated megabase-long genomic DNA from the blood of the same individual for Bionano optical mapping (60) as explained in ref. 40. The DNA was digested with the restriction enzyme BsPQI, fluorescently labeled on the cutting sites, and imaged on an Irys instrument at the VIB Nucleomics Core (Leuven, Belgium). We obtained 1.7 million molecules with an average length of 243.87 kb after filtering out those shorter than 150 kb.

### Genome Assembly.

We de novo assembled all reads of the 10x Chromium library with Supernova v2.0.1 (39) on a server node with 28 cores and 512 GB of RAM. The sequencing data corresponded to a 75× coverage, i.e., above the recommended depth of 56× (39). Downstream analyses were based on the pseudohaplotype Supernova output. A BUSCO v3.0.2 (61) gene completeness analysis revealed the presence of duplicated scaffolds, i.e., scaffolds with similar lengths that presented the same set of genes and hence corresponded to the two haplotypes in the diploid genome. We only kept the longest scaffold of each duplicated pair based on the BUSCO results. We identified additional duplicated scaffolds by looking for sequences greater than 50 kb with similar lengths and sharing a vertex in the Supernova assembly graph. We ran a megablast (62) search on those sequences against themselves and filtered out self-matches. High coverage and great percentage of identity were used as the criteria to confirm duplication, and the shortest sequence of the pair was then removed. In addition, we removed identical scaffolds with CD-HIT-EST (parameters: -aS 1 -g 1 -s 1 -c 1). Finally, we removed mitochondrial DNA scaffolds and looked for the presence of contaminants running blastn searches against the *P. guttatus* mtDNA (GenBank accession number: AM236349.1), UniVec Build 10, *Saccharomyces cerevisiae*, *Escherichia coli*, and reptilian ferlaviruses databases with the NCBI VecScreen search parameters.

We assembled de novo the Bionano Irys molecules into genome maps on a server with an IrysSolve installation and with the optArgumets_human.xml configuration file. We ran five extension-and-merge iterations considering the filtered Supernova assembly for autonoise parameter estimation. Hybrid scaffolds were produced with Bionano Solve v3.2.1 combining the genome maps and the final Supernova scaffolds. We considered the configuration file hybridScaffold_config_aggressive.xml to resolve any conflicts between the Supernova and Bionano genome maps (parameters: -N 2 -B 2). The final genome assembly includes the hybrid scaffolds and the nonscaffolded sequences of the Supernova assembly.

We assessed the gene completeness of the assembly with BUSCO v3.0.2, focusing on the 3,950 single-copy genes in the Tetrapoda set. As a means to assess genome continuity, we performed blastx searches against other Squamata HOX and DLX proteins to confirm the presence of each cluster in a single scaffold and verify its conserved synteny.

We also modeled repeat families for the corn snake with RepeatModeler v1.0.11 (63). We masked the assembly with RepeatMasker v4.0.7 (64) in sensitive search mode with rmblastn v2.2.27+ in three sequential steps, taking the masked output genome of each run as input for the following. First, we considered all of the Tetrapoda repeats from the RepeatMasker database (Repbase release 20170127). Second, we looked for classified corn snake repetitive elements from RepeatModeler (including the LTR-Retrotransposon from ref. 25), and, finally, we retrieved the repeats with an unknown classification.

### RNA Extraction and Sequencing.

We collected samples from brain, testis, ovary, cerebellum, kidney, liver, heart, and skin from either male or female samples (*SI Appendix*, Table S6). RNA was extracted using the RNeasy protocol from Qiagen. RNeasy Micro columns (no. 74004: Qiagen) and RNeasy Mini columns (no. 74104; Qiagen) were used to extract RNA from small (<5 mg) and larger (>5 mg) samples, respectively. The tissues were homogenized in RLT buffer supplemented with 40 mM dithiothreitol (DTT) or QIAzol. RNA quality was assessed using the Fragment Analyzer (Advanced Analytical); RQN values were greater than 6.7 for all samples. The RNA-seq libraries were prepared using the TruSeq Stranded mRNA LT Sample Prep Kit (Illumina) and sequenced on the HiSeq 2500 platform. We obtained 23 to 62 million 100-bp strand-specific single-end reads per library. We filtered the data with Trimmomatic v0.32 (65), removing TruSeq adapters, quality-trimming reads using LEADING:3 TRAILING:3 MAXINFO:50:0.9 parameters, and filtering out sequences shorter than 50 bp. Then, we removed any reads with unknown nucleotides with sickle v1.33 (66). The same Trimmomatic filtering was used for the Illumina paired-end reads from ref. 67.

### Genome Annotation.

We first performed a transcriptome template assembly using 1) Illumina paired-end corn snake libraries from ref. 67 originating from adult tissues (brain, kidney, and testis) and different developmental stages (embryonic day 10, 30, and 47), 2) the filtered Illumina single-end VNO libraries from ref. 68; 3) 14 Illumina single-end libraries from different corn snake organs sequenced here, and 4) five paired-end libraries from the NCBI SRA repository from corn snake skin, as well as scent and salivary glands (69) (*SI Appendix*, Table S6). Each library was aligned to the unmasked genome assembly with HISAT2 v2.1.0 (70), and the transcripts were assembled and merged with StringTie v1.3.4d (71). We input the merged HISAT2 alignments and the soft masked genome into the BRAKER v2.1 gene annotation pipeline (72). The GeneMark-ET v4.33 (73) and Augustus v3.3 (74) gene predictors were trained automatically.

In addition, we ran the MAKER v3 gene annotation pipeline (75) using as evidence the transcriptome template assembly, the *Ophiophagus hannah* proteins, and the Squamata proteins from GigaDB and RefSeq (accessed May 31, 2018). We ran the first annotation round using EVidenceModeler (EVM) v1.1.1 (76), SNAP v2013-11-29 (77), and GeneMark-ES as gene predictors. We trained the SNAP hidden Markov models (HMM) with the Tetrapoda BUSCO output and took the optimized GeneMark parameters from the BRAKER annotation. We masked the genome with the repetitive families identified by RepeatModeler during the annotation process. This allows to distinguish simple repeats, soft masked in MAKER (i.e., converted into lowercase), from the others that are hard masked (i.e., replaced by Ns). We only kept gene models supported by evidence data alignments (proteins or transcripts) and not solely produced by a gene predictor. We then retrained the SNAP HMM using the first iteration output and also included Augustus v3.3 as a gene predictor for the second run considering the optimized parameters obtained from the Tetrapoda BUSCO analysis. For this second round, we input the repeat masking information from the previous step, did not allow for gene models coming solely from protein or transcript alignments, disregarded alternative splicing isoforms, and kept predicted gene models with no evidence data support (Annotation Edit Distance [AED] equal to 1). Then, we ran InterProScan v5.29–68.0 (78) to look for Pfam protein domains. The final gene set comprises the gene models with an AED <1 and those with an AED of 1 and a protein domain predicted. We calculated the AED of the BRAKER gene models by inputting them into the MAKER pipeline in GFF format together with the protein and transcriptome databases and the repetitive elements information. No gene predictors were considered in this case. Then, as for MAKER, we ran InterProScan and kept only those models that had an AED <1 or an identified Pfam protein domain. We performed blastp searches against UniProtKB (accessed May 8, 2018) and Squamata proteins to annotate the BRAKER and MAKER gene sets. A protein was considered complete if it covered at least 95% of the best blastp hit length.

### Crosses and Deep Sequencing of Lavender Individuals.

We crossed a lavender male (*lavender*/*lavender* genotype) and a wild type (WT) female with an heterozygous (*lavender*/*+*) genotype to obtain homozygous and heterozygous offspring. Genomic DNA was extracted from blood samples of the parents and six offspring individuals using the QIAamp DNA Blood mini kit (no. 511004; Qiagen) and from scale clips of 34 hatchlings using the DNeasy Blood and Tissue kit (no. 69504; Qiagen). We pooled separately DNA samples of 20 *lavender*/*lavender* offspring and 20 *lavender*/+ ones in equimolar concentrations. We constructed genomic DNA libraries of 350-bp fragment size for each of the two parents and one library for each of the two offspring pools with the TruSeq DNA PCR Free kit. We separately sequenced (Macrogen) the four libraries using an Illumina HiSeq X instrument, producing 151-bp paired-end reads. We obtained 169 to 244 million paired-end reads per library and checked data quality and for the absence of adapters with FASTQC. We filtered the data with sickle v1.33 (66), removing flanking bases of quality lower than 20 and discarding reads with unknown bases or shorter than 50 bp after trimming. We retained between 155 and 232 million reads, which corresponds to a 27.5 to 41× average coverage of a 1.7-Gb genome (*SI Appendix*, Table S7). Genomic DNA from five additional individuals was sequenced in the same way, besides the PG7 library, which comes from ref. 27. We performed the same quality filtering as for the lavender family libraries, including an initial trimming step of the 10x Genomics barcodes from the genome individual library using LongRanger basic v2.2.2 and then by removing reads that did not have a whitelisted barcode.

### Variant Calling and Confirmation.

To retrieve the genomic interval bearing the *lavender* locus, we aligned the four genomic libraries of the parents and offspring against the corn snake genome using bwa v0.7.16 (79) and default parameters in mem mode. We converted the output SAM files into BAM and sorted them out by their leftmost coordinates with SAMtools v1.5 (80). PCR duplicates were removed with SAMtools v0.1.19. Variants were identified with Platypus v0.8.1 (81) in callVariants mode, keeping insertions/deletions (indels) alongside nucleotide polymorphisms and allowing for a minimum read mapping quality of 30 and for a minimum base-calling quality of 20 (parameters: –genIndels = 1, –minMapQual = 30, and –minBaseQual = 20).

To locate the genomic interval, we extracted from the VCF file the biallelic single nucleotide polymorphisms (SNPs) and multinucleotide polymorphisms (MNPs) that 1) had a Platypus-estimated quality >100, 2) passed all of the variant caller filters, 3) were present in nonrepetitive elements, 4) had a coverage greater than eight, and 5) had a minimum genotype quality of 20 for each library. We further selected those variants that were segregating according to the expected genotypes for the causal locus in each library (homozygous for the *lavender*/*lavender* libraries and heterozygous for the WT *lavender*/^+^ libraries). More specifically, we queried the parental libraries on one hand and all four libraries on the other hand for polymorphisms cosegregating with the *lavender* variant. We compared these two sets of filtered SNPs/MNPs, looking for intervals that have at least three consecutive variants cosegregating with the *lavender* genotype and allowing for a maximum of two successive mismatches between the two sets of filtered SNPs/MNPs. To complement, we used a sliding window approach, with a 1-Mb window size and a 100-kb step, to calculate the proportion of cosegregating variants by comparing the two sets of filtered SNPs/MNPs in scaffolds longer than 100 kb.

We compared the selected scaffolds against the masked *A. carolinensis* v2.0 (GCF_000090745.1) and *G. gallus* v6.0 (GCF_000002315.6) genomes to locate, order, and orient them. We used megablast with default parameters, keeping the best hit for each position in the sequence if it had a bitscore >100. Using in-house Python scripts, we 1) extracted all of the BRAKER and MAKER genes in the interval, 2) located any cosegregating variants (SNP/MNP or indels) within these genes or in the flanking 3,000 bp (upstream and downstream), and 3) verified the presence of any modifications, such as frameshifts and premature stop codons, in the coding sequence or in the intron/exon boundaries. The primers for the *LYST* variant verification on cDNA and gDNA samples are provided in *SI Appendix*, Table S8.

To further reduce the genomic interval, we performed extended mapping analyses. We repeated the variant calling process as described above with the four family libraries and five additional individuals: 1) an unrelated *lavender/+* individual, 2) the genome individual (+/+), and 3) three unrelated +/+ individuals, one of which is a wild-caught animal. We looked in the genomic interval defined by the family mapping for variants, including biallelic indels, cosegregating with the *lavender* genotype.

### Histology.

Dorsal and ventral skin was fixed in 4% PFA and dehydrated in ethanol before embedding in paraffin blocks. Seven-microgram microtome sections were deparaffinized and directly mounted with Ultrakitt (no. 3921; JT Baker) to image the chromatophores with a Pannoramic MIDI slide scanner. Liver cryosections were stained with Oil Red O as described previously (82).

### Transmission Electron Microscopy.

Skin pieces of 1 mm^2^ were fixed in 2.5% glutaraldehyde solution (EMS) in phosphate buffer (PB 0.1 M, pH 7.4; Sigma-Aldrich) for 2 h at room temperature (RT). They were then rinsed three times for 5 min in PB buffer and postfixed by a fresh mixture of osmium tetroxide 1% (EMS) with 1.5% potassium ferrocyanide (Sigma-Aldrich) in PB buffer for 2 h at RT. The samples were washed three times in distilled water and dehydrated in acetone solutions (Sigma-Aldrich) at graded concentrations (30% for 40 min; 70% for 40 min; 100% for 1 h; 100% for 2 h). This was followed by infiltration in Epon (Sigma-Aldrich) at graded concentrations (Epon:acetone 1:3 for 2 h; Epon:acetone 3:1 for 2 h, Epon for 4 h; Epon for 12 h) and finally polymerization for 48 h at 60 °C. Ultrathin sections of 50 nm were cut transversally on a Leica Ultracut (Leica Mikrosysteme) and picked up on copper slot grids 2 × 1 mm (EMS) coated with a polystyrene film (Sigma). Sections were poststained with 2% uranyl acetate (Sigma) in H_2_O for 10 min, rinsed several times with H_2_O followed by Reynolds lead citrate for 10 min, and rinsed several times with H_2_O. Micrographs were taken with a transmission electron microscope (Philips CM100; Thermo Fisher Scientific) at an acceleration voltage of 80 kV with a TVIPS TemCam-F416 digital camera (TVIPS). Large montage alignments were performed using Blendmont command-line from the IMOD software (83).

To assess differences in the shape of the iridophore crystals in wild type and lavender individuals, we used “analyze particles” of ImageJ to compute their roundness and solidity. The former compares the crystal’s area with that of a circle having a diameter equal to the major axis of a fitted ellipse [4*(area)/(pi*[major axis]^2^)], whereas the latter is the ratio of the crystal’s area to the area of the smallest convex object surrounding the crystal. The first shape descriptor quantifies how elongated versus round an object is (roundness = 1 corresponding to a circle), and the second describes how ragged is its contour (solidity = 1 corresponding to a fully convex polygonal shape). Crystals were manually delineated on TEM images.

### LAMP1 Immunostaining.

Immunohistochemistry was performed on 7-μm paraffin sections of wild type and lavender liver samples using LAMP1 (Lysosomal-associated membrane protein 1; ab24170; Abcam) primary antibody as a lysosome membrane marker, with a citrate pH 6 antigen retrieval. We used Alexa Fluor 488 (A-11008; Thermo Fisher Scientific) as secondary antibody, and nuclei were stained with ProLong Gold antifade reagent with DAPI (P36935; Invitrogen). Images were acquired with a Zeiss LSM800 confocal microscope.

## Supplementary Material

Supplementary File

## Data Availability

Genome assembly data have been deposited to the National Center for Biotechnology Information database (https://www.ncbi.nlm.nih.gov/assembly/GCF_001185365.1).
