# Supplementary material for: Genome mapping of a LYST mutation in corn snakes indicates that vertebrate chromatophore vesicles are lysosome-related organelles
Source: Proc Natl Acad Sci U S A. 2020 Oct 5;117(42):26307–17. doi: 10.1073/pnas.2003724117 (PMC7584913; doi:10.1073/pnas.2003724117)
Supplement: Supplementary File [file pnas.2003724117.sapp.pdf]

PNAS
www.pnas.org

Supplementary Information for

Genome mapping of a *LYST* mutation in corn snakes indicates that vertebrate chromatophore vesicles are lysosome-related organelles

Asier Ullate-Agote [a,b,c], Ingrid Burgelin [a], Adrien Debry [a], Carine Langrez [a], Florent Montange [a], Rodrigue Peraldi [a], Jean Daraspe [d], Henrik Kaessmann [e], Michel C. Milinkovitch [a,b,c], Athanasia C. Tzika [a,b,c,1]

[a] Laboratory of Artificial & Natural Evolution (LANE), Department of Genetics & Evolution, University of Geneva, CH-1211 Geneva, Switzerland
[b] SIB Swiss Institute of Bioinformatics, Switzerland
[c] Institute of Genetics and Genomics of Geneva (iGE3), University of Geneva, Geneva, Switzerland
[d] University of Lausanne, Faculté de biologie et de médecine, Electron Microscopy Facility, CH-1015 Lausanne, Switzerland
[e] Center for Molecular Biology of Heidelberg University (ZMBH), DKFZ-ZMBH Alliance, D-69120, Heidelberg, Germany

[1] Athanasia C. Tzika
Laboratory of Artificial & Natural Evolution (LANE), Department of Genetics & Evolution, University of Geneva, CH-1211 Geneva, Switzerland
Email: athanasia.tzika@unige.ch

Asier Ullate-Agote https://orcid.org/0000-0002-8595-7703
Henrik Kaessmann https://orcid.org/0000-0001-7563-839X
Michel Milinkovitch https://orcid.org/0000-0002-2553-0724
Athanasia C. Tzika https://orcid.org/0000-0002-0831-5221

Classification
BIOLOGICAL SCIENCES: Genetics

Supplementary Text

Origin of the Lavender morph

Since the 1960s, the corn snake is a popular species among reptile breeders. The usual practice is for an owner to have a few snakes that he breeds and, due to inbreeding, individuals homozygous for recessive mutations are produced. In this manner, more than twenty spontaneously-occurring color and color pattern morphs have appeared in the pet trade and are now selected and often mixed to produce spectacular phenotypes. Animals from the wild are also collected from time to time enriching the diversity of the captive population. This is the case of the Lavender morph described in this article.

Originally, a wild-caught animal from the Sarasota-Punta Gorda area of Florida's lower western coast was crossed with a captive animal; a Lavender offspring appeared in the F2 generation. Already for the first individual, they noticed that "its slightly unusual chocolate brown appearance as a hatchling gradually changed as the animal grew and it lost the reddish tones." Descendants of the original Lavender have since been crossed with different morphs to produce mutants with more pale pigmentation. The founders of our colony were three related Lavender individuals, but not necessarily siblings. We also have a few individuals of other morphs that are heterozygous for the Lavender allele (Table S4).

Reference

Love K. & Love B. (2005) Corn Snakes: The comprehensive owner's guide. *Editions Advanced Vivarium Systems*

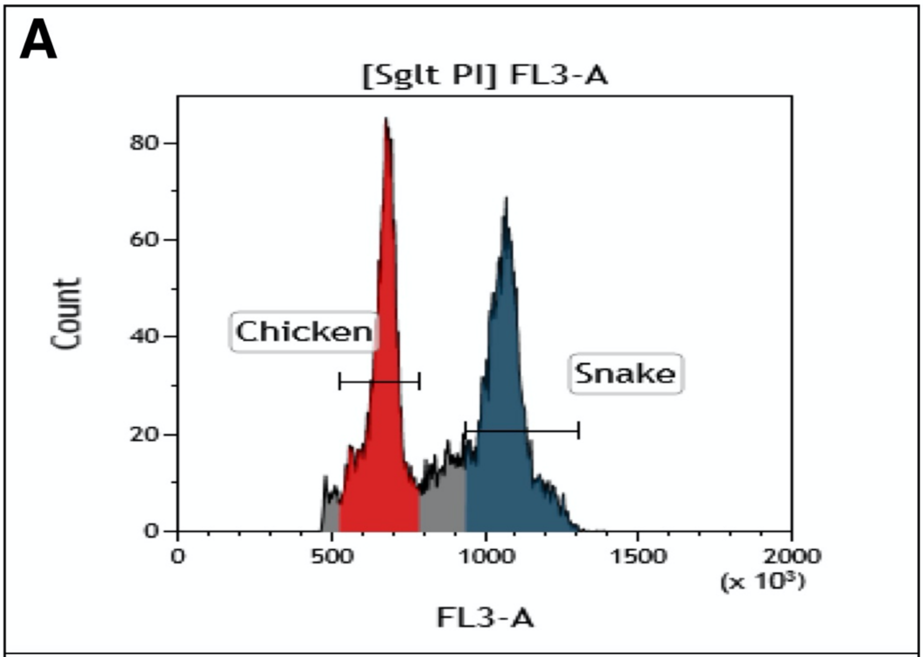

B

Gate	Number	%Total	%Gated	X-Median	Genome size (Gb)
All	9'680	47.34	100	889'360.56	
Chicken	3'941	19.27	40.71	675'183.69	1.065
Corn snake	4'472	21.87	46.2	1'065'714.75	1.68

Figure S1. FACS analyses of chicken and corn snake nucleated erythrocytes. The chicken genome size is based on the latest v6.0 assembly (GCF_000002315.6).

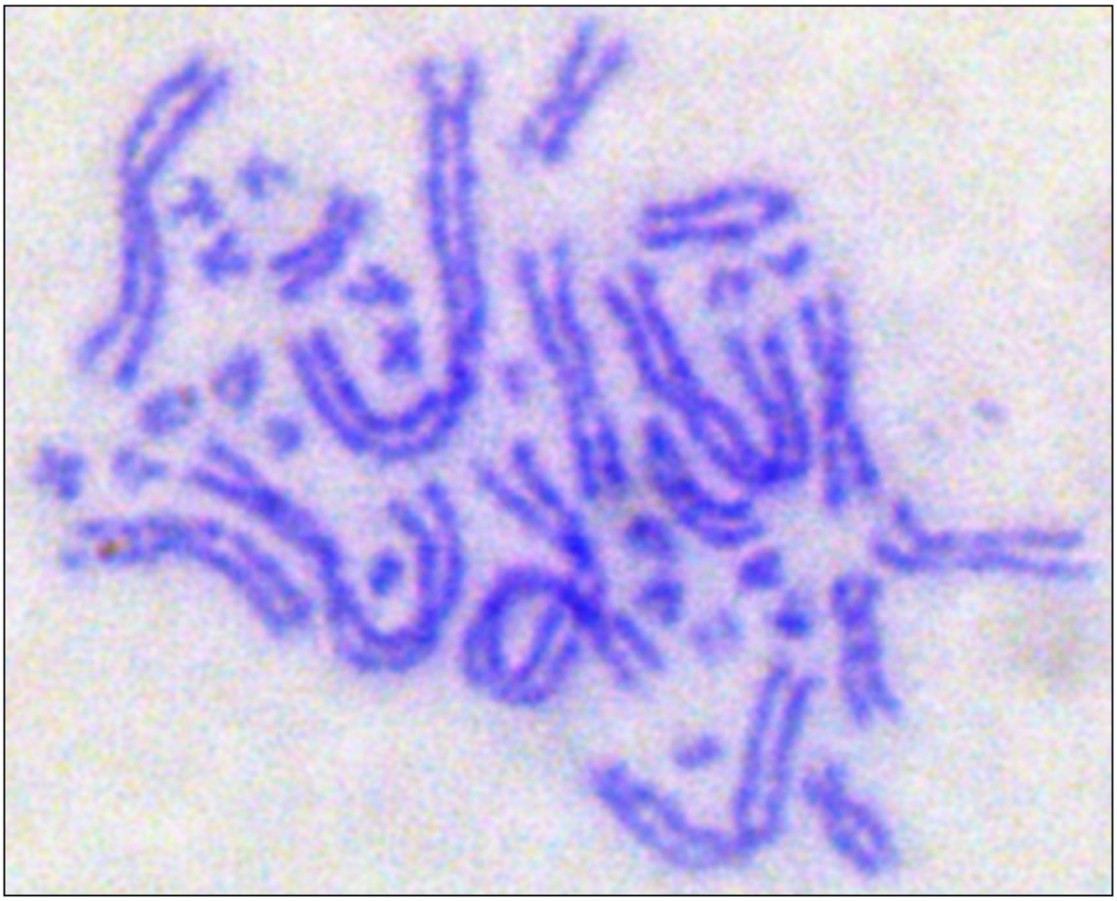

Figure S2. Corn snake karyotype. Chromosomes from a corn snake fibroblast are Giemsa-stained.

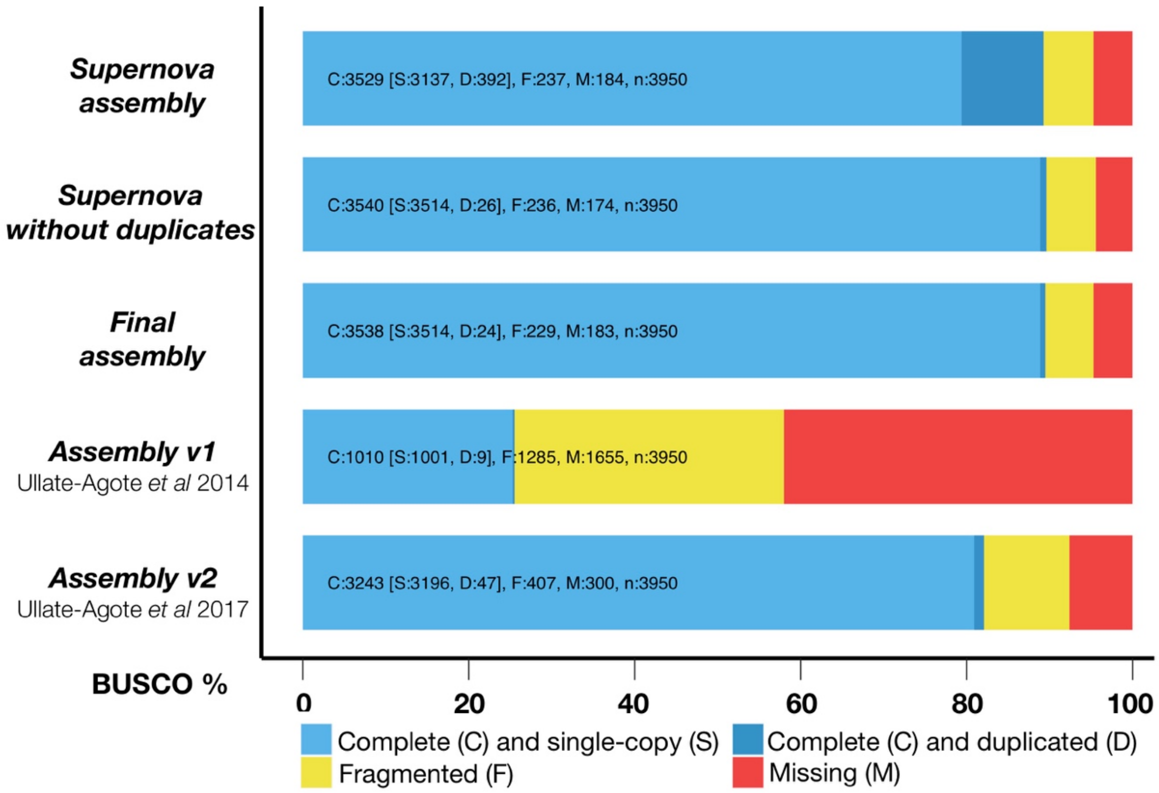

Figure S3. BUSCO results for the three assembly steps performed here and the previously published corn snake genomes. The genome completeness assessment was performed using the 3,950 single-copy genes of the Tetrapoda set.

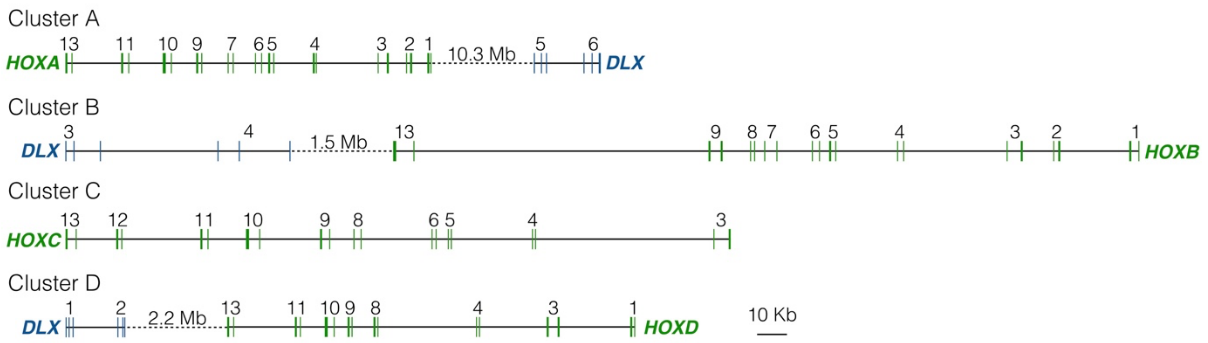

Figure S4. Organization of the *HOX* and *DLX* clusters in our corn snake genome assembly. Each vertical line corresponds to an exon. *HOX* genes are represented in green, *DLX* genes in blue. The distance between the *HOX* and *DLX* clusters is represented with a dashed line and is not proportional to scale. Numbers above each vertical line correspond to the gene number within each cluster.

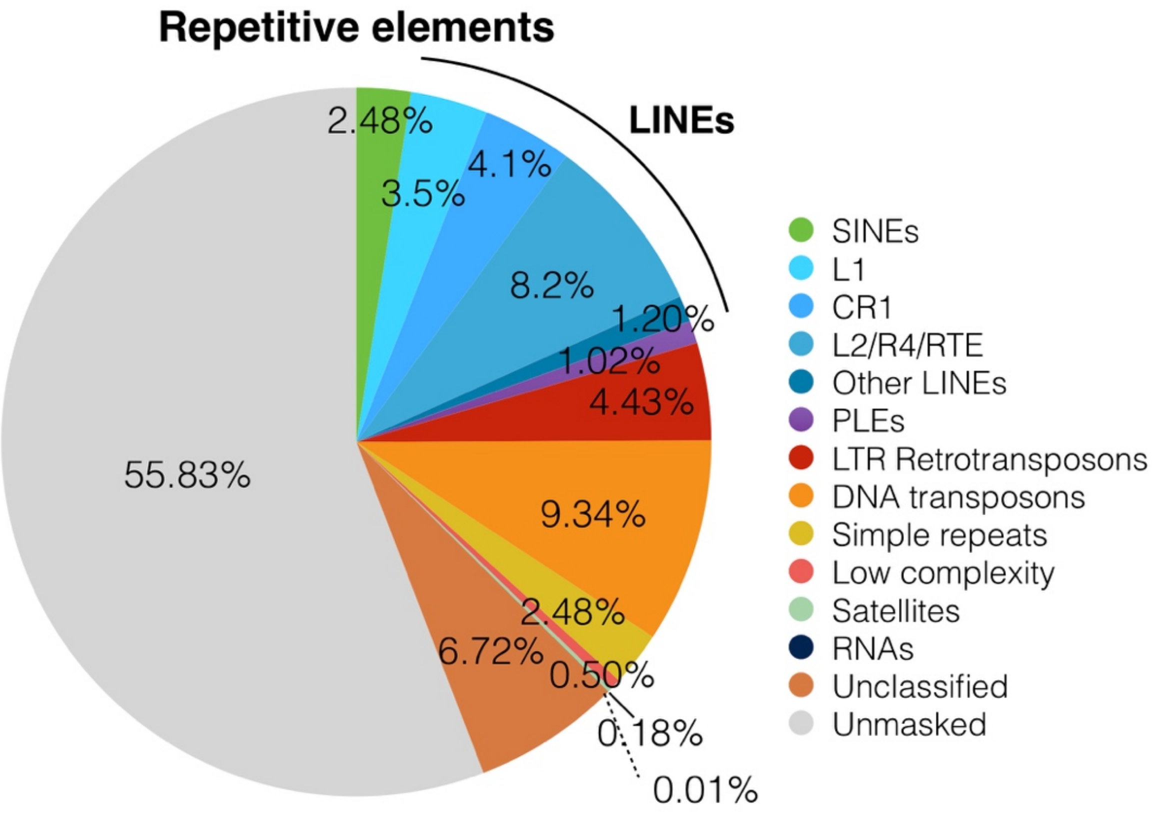

Figure S5. Proportions of repetitive element families in the corn snake genome assembly identified by RepeatMasker analyses.

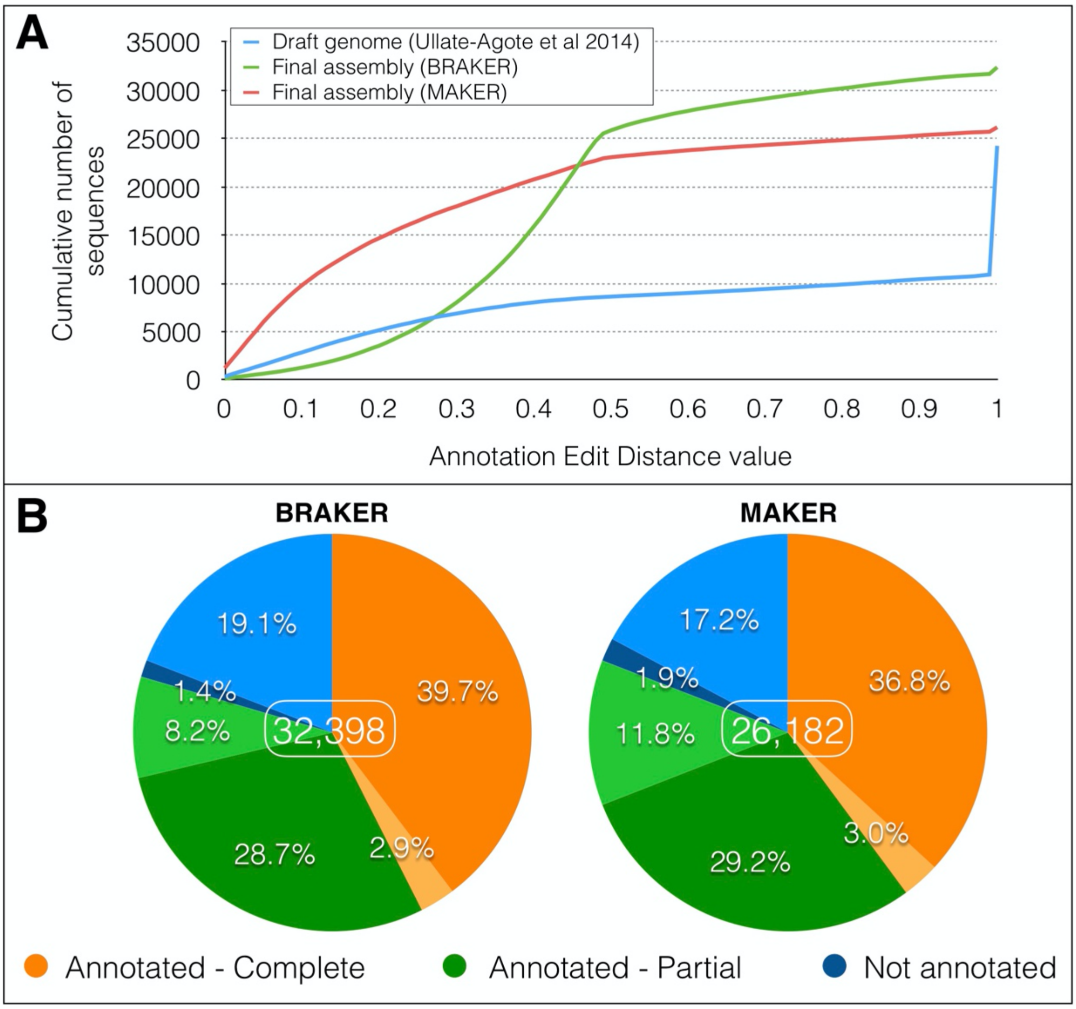

Figure S6. Overview of MAKER and BRAKER annotations. *(A)* Cumulative number of sequences below a certain annotation edit distance (AED). AED equal to 1 means that there is no support by evidence data. Blue line: MAKER annotation of the draft corn snake genome; red and green: the MAKER and BRAKER annotations, respectively, of the high-quality assembly presented here. *(B)* Proportions of modelled BRAKER and MAKER proteins annotated with data from other squamates. The total number of proteins is shown at the center of each pie chart. A protein is considered to be complete if it covers at least 95% of the best blastp hit length. The darker shades of orange, green and blue represent proteins with a Pfam domain.

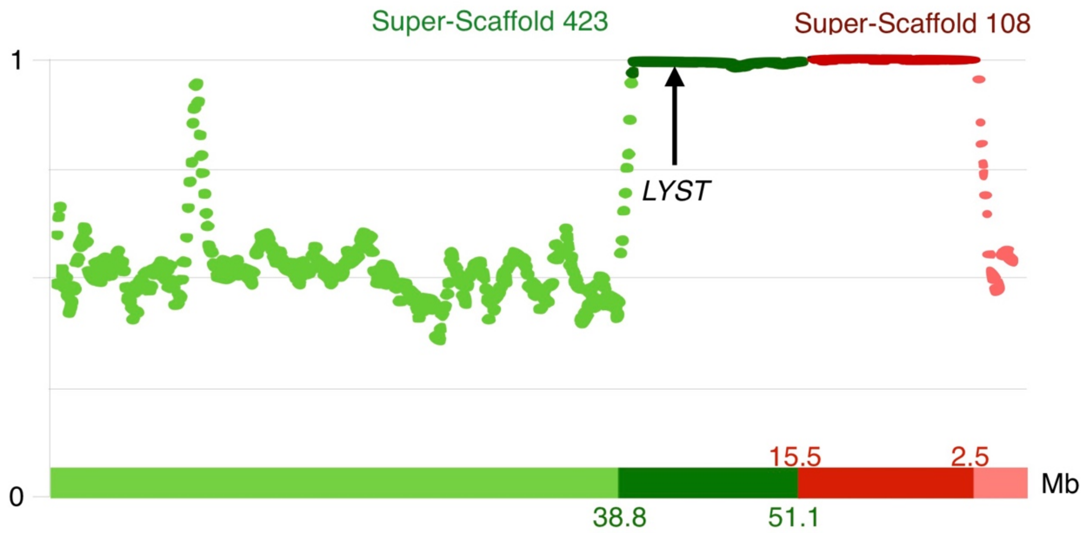

Figure S7. Close up of the genome interval harboring the *lavender* locus. Proportion (y-axis) of quality-filtered SNP/MNP co-segregating with the *lavender* locus in the four genome libraries compared to informative quality-filtered parental variants (homozygous in the *lavender*/*lavender* father and heterozygous in the *lavender*/+ female). The proportion was calculated in overlapping windows of 1 Mb with a step of 100 Kb. Scaffolds are ordered and oriented based on their synteny to the *G. gallus* and *A. carolinensis* genomes. Dark green and dark red dots correspond to the 25.3 Mb region with the highest proportion of co-segregating variants. *LYST* is located in this interval.

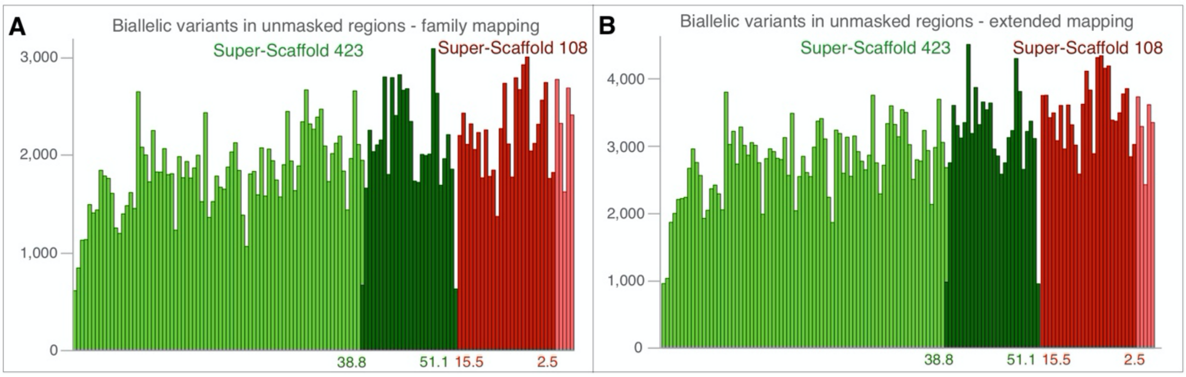

Figure S8. Total (co-segregating and not co-segregating) biallelic variants (SNP/MNP and indels) in Super-scaffolds 423 and 108 based on the family (*A*) and the extended mapping (*B*). Note that, for the two mapping analyses, the distribution of biallelic SNPs on the two scaffolds is homogeneous, compared to the peak of biallelic SNPs that co-segregate with the Lavender phenotype (see main Figure 3) in the same intervals. Dark green and dark red bins correspond to the 25.3 Mb region with the highest proportion of co-segregating variants based on the family mapping. Each bin in the histograms represents a 500 Kb interval.

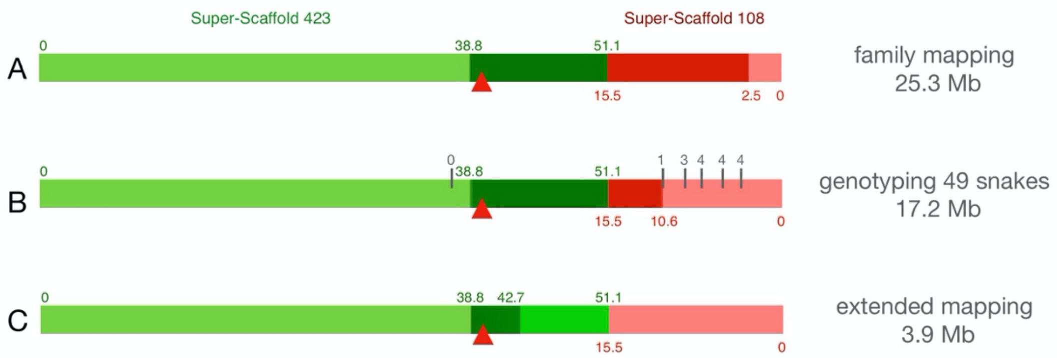

Figure S9. Mapping of the Lavender interval. The original interval of 25.3 Mb obtained with the family mapping (*A*) was reduced to 17.2 Mb after genotyping 49 snakes of the Lavender lineage (*B*). Vertical lines indicate the position of the genotyped SNPs and the number of recombinants found at each position is given above. (*C*) The final interval of 3.9 Mb generated from the extended mapping after adding individuals of other lineages. Super-Scaffolds 423 and 108 are represented in green and red, respectively. Darker areas correspond to the resulting interval at each step of the mapping process. The red arrow head shows the position of *LYST*.

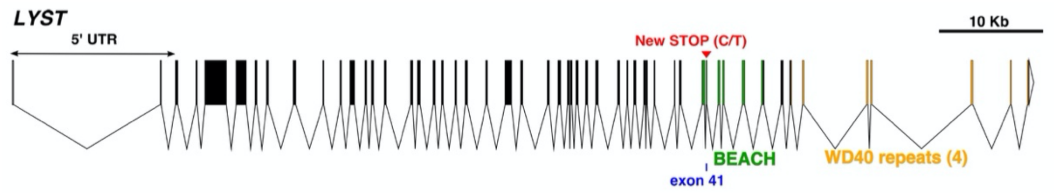

Figure S10. Exon-intron structure of *LYST* based on the MAKER gene model. The first two exons and part of the third correspond to the 5’ UTR. A single base substitution in exon 41 introduces an early STOP codon, affecting the BEACH domain and the four WD40 repeats downstream.

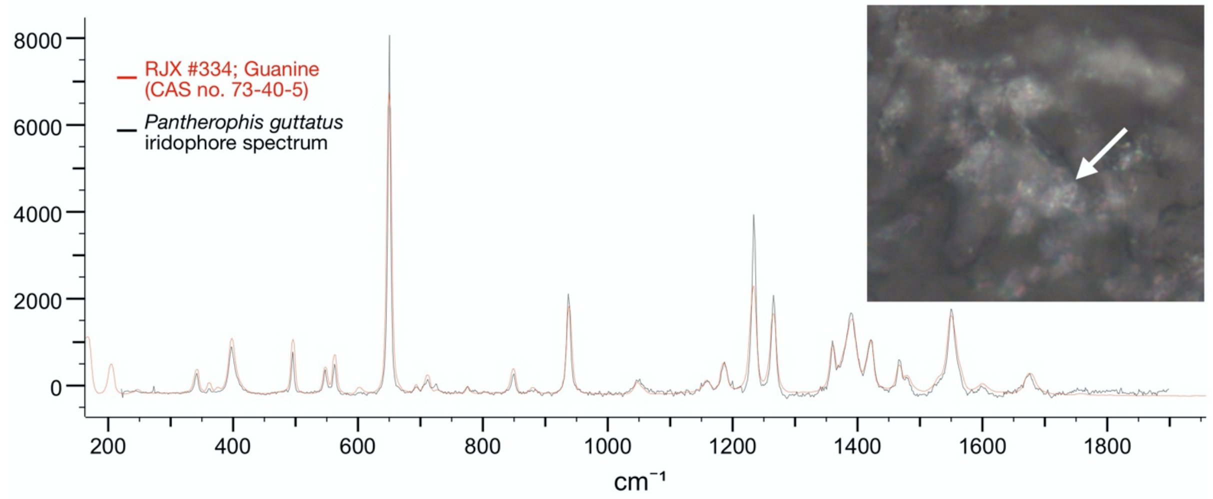

Figure S11. Raman spectroscopy was performed on a 10 μm cryosection of ventral white skin from a wild type that only contains iridophores. The chemical structure of a target molecule is determined by measuring the energy shift of an incident laser light after its interaction with the molecule. The spectrum was collected on a Horiba Labram HR evolution spectrometer. The excitation source was a solid-state laser at 532 nm. The spectrum (black line) was matched against the KnowItAll database (Bio-Rad) of confirmed spectra and clearly supports the presence of guanine (red line) in the reflecting platelets of the iridophores. The white arrow in the inset points to the iridescent iridophore on which the spectrum was collected.

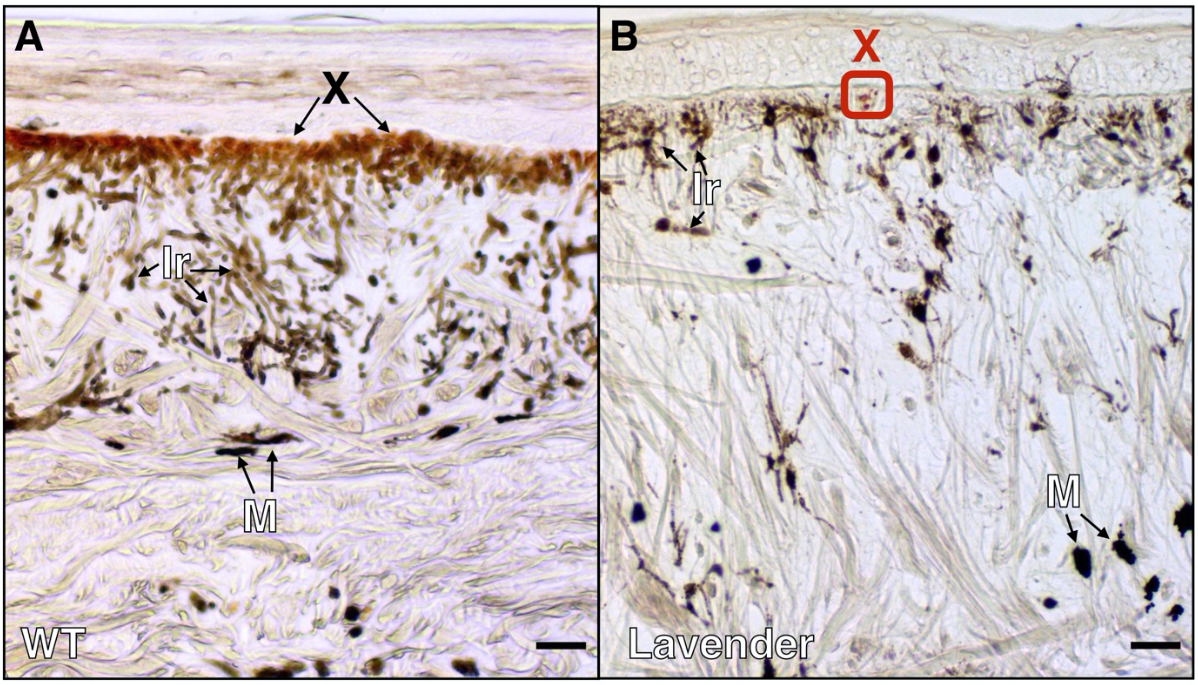

Figure S12. Histological sections of wild type and Lavender dorsal skin. *(A)* In the wild type background skin, we observe a compact layer of xanthophores at the top of the dermis, scarce melanocytes at the bottom, and dispersed iridophores in between. Collagen fibers appear as semi-transparent. *(B)* In the Lavender background skin, the number of pigmented chromatophores is reduced, especially xanthophores (cf. red frame). No obvious effect of the lavender mutation on other skin cell types is detectable from these histological sections. X: xanthophores, Ir: iridophores, M: melanophores. Scale bars: 50 µm.

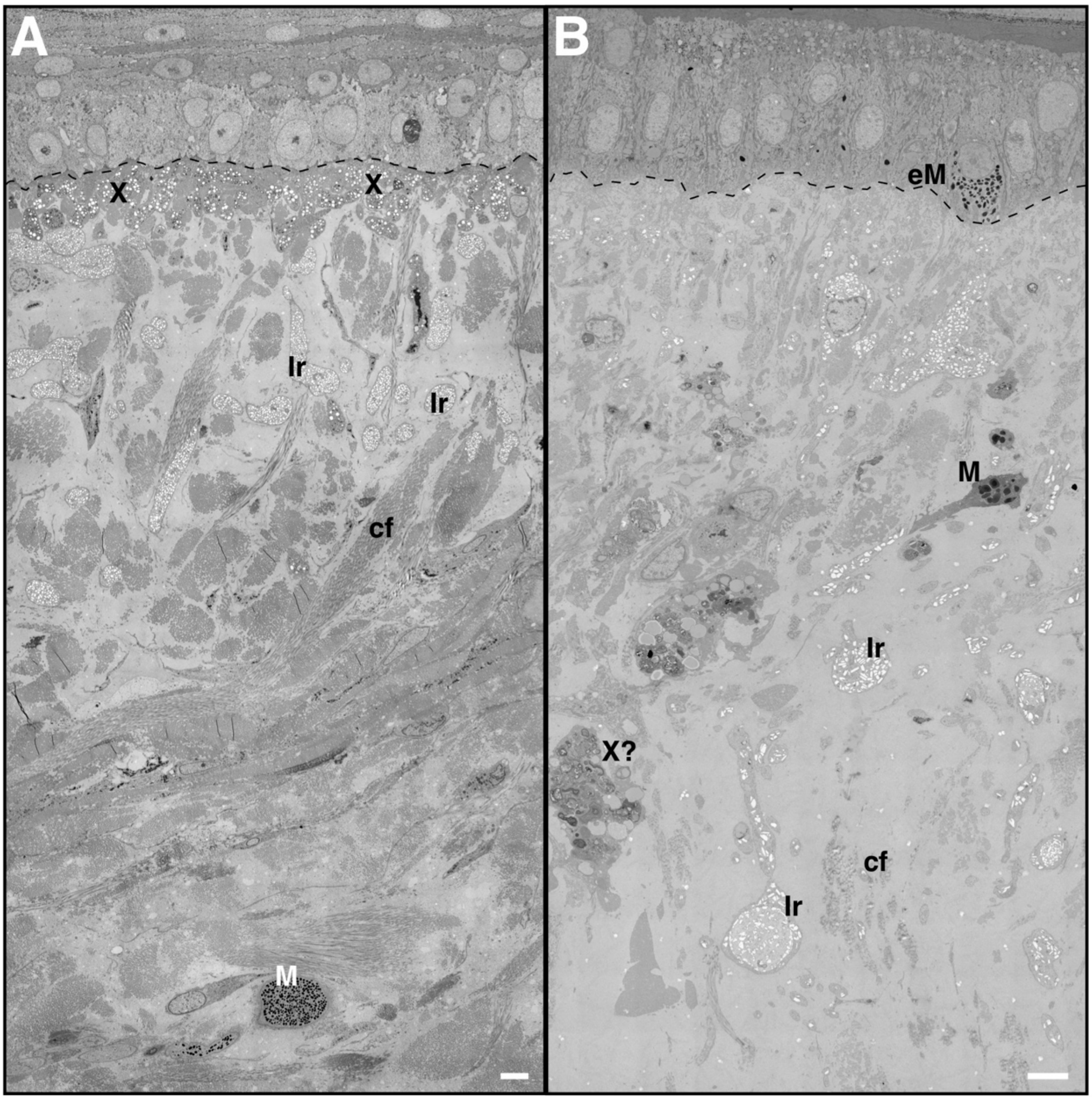

Figure S13. Images of transmission electron microscopy semi-thin sections of the background dorsal skin of a wild type and a Lavender. *(A)* In the wild type corn snake, the xanthophores (X) are tightly packed just under the epidermis (dashed line: basal membrane), the iridophores (Ir) are spread in the loose dermis among collagen fibers (cf), and the melanophores (M) are situated deeper in the dermis. Scarce xanthophores are also seen in the loose dermis. *(B)* In the Lavender, iridophores and melanocytes are less numerous and their internal structure is affected. Xanthophores, if present, are difficult to identify. They are either situated near the basal membrane (dashed line) and lack xanthosomes, or they are deeper in the dermis and contain xanthosomes with malformed lamellae. X: xanthophores, Ir: iridophores, eM: epidermal melanophore, M: dermal melanophores, cf: collagen fibers. Scale bars: 5 µm.

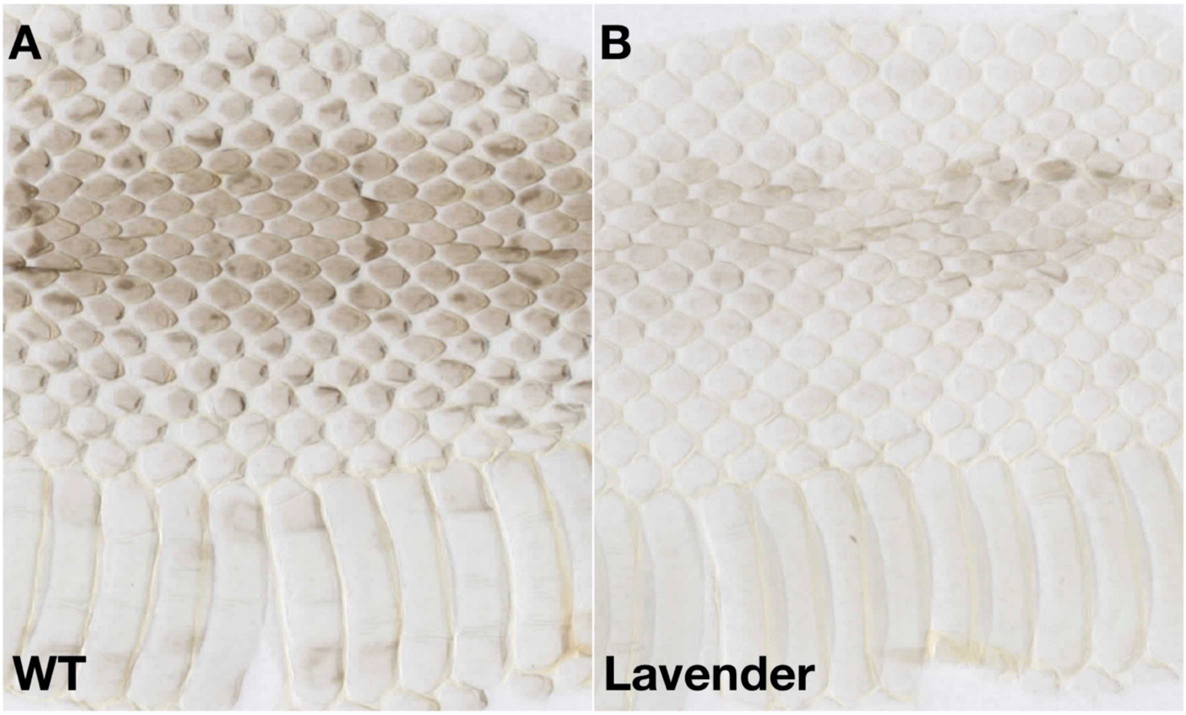

Figure S14. Shed skin of *(A)* wild type and *(B)* Lavender individuals. The sheds were collected when both animals were three-years old. Sheds are epidermal, hence, they maintain the pigmentation due to the epidermal melanophores only. Note the faint checkered pattern on the ventral large scales indicating the presence of epidermal melanophores.

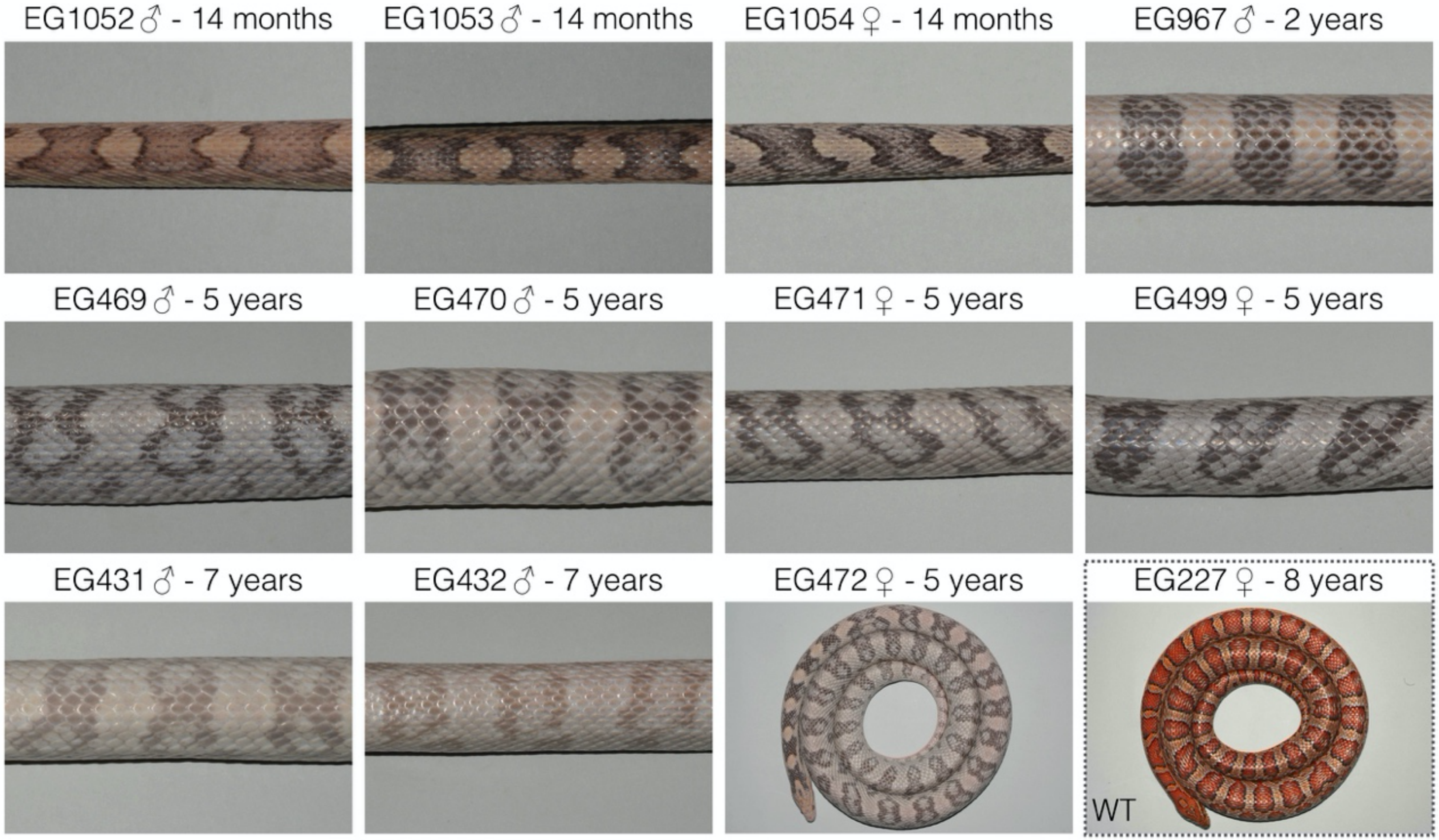

Figure S15. Variability in the coloration of the Lavender individuals in our colony. The intensity of the pink and black coloration is variable among the individuals. A wild type individual is shown for comparison at the lower right corner.

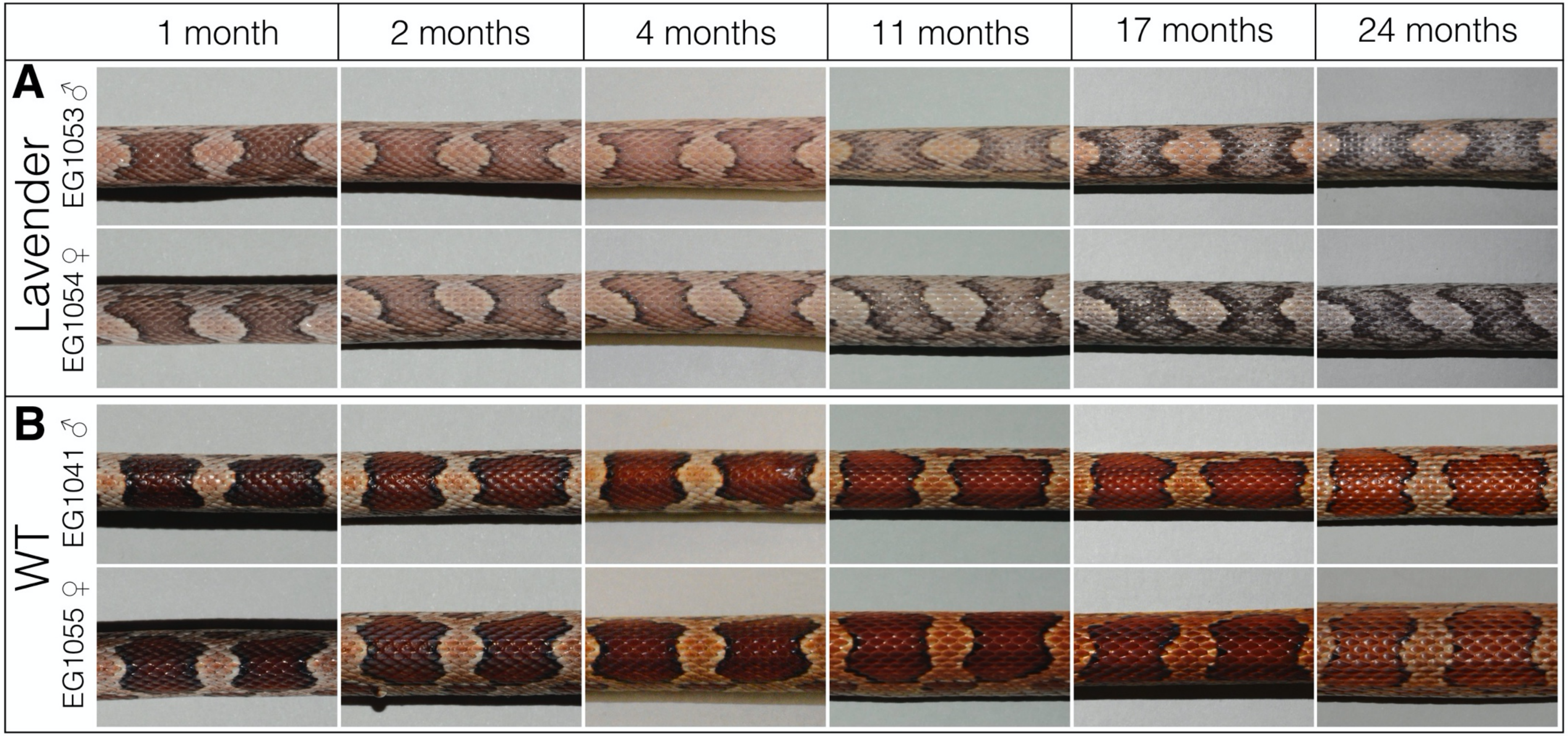

Figure S16. The coloration of wild type and Lavender individuals changes during their first two years of post-hatching development. *(A)* In Lavender individuals, the pink coloration slowly fades as the animal grows. *(B)* In wild type animals, the orange background coloration becomes more intense and the blotches become more vivid red.

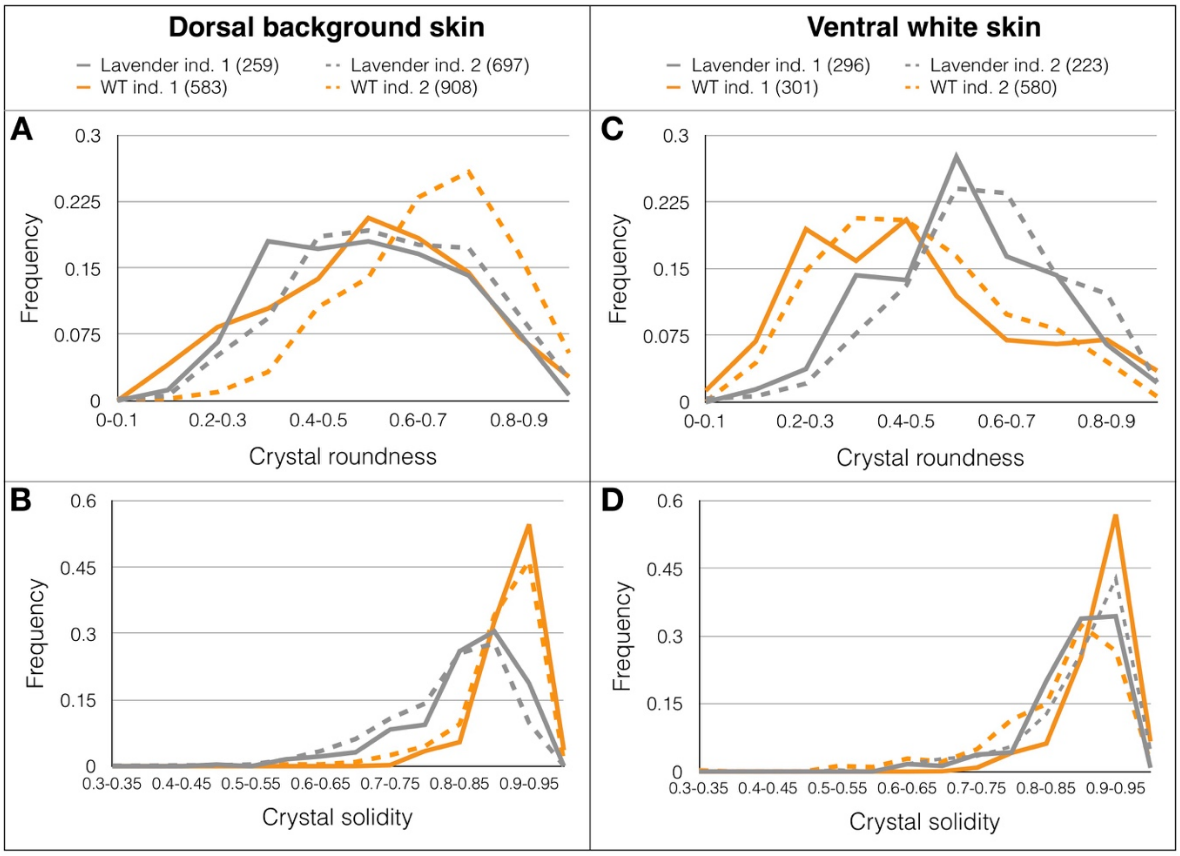

Figure S17. Distributions of shape descriptors (roundness and solidity) of the iridophore guanine crystals in the dorsal (*A,B*) and ventral (*C,D*) skin of two wild type individuals (orange dotted and plain lines) and two Lavender individuals (grey lines). The number of crystals is given in parenthesis for each individual (cf. graph legends). Categories are at 0.1 intervals for roundness and 0.05 intervals for solidity. Roundness (how much a crystal is close to a disc) and solidity (how convex the outline of the crystal is) are computed as indicated in the Methods section.

	WT EG1074	WT EG1075	Lavender EG1052	Lavender EG1053	Lavender EG1054
**Liver**	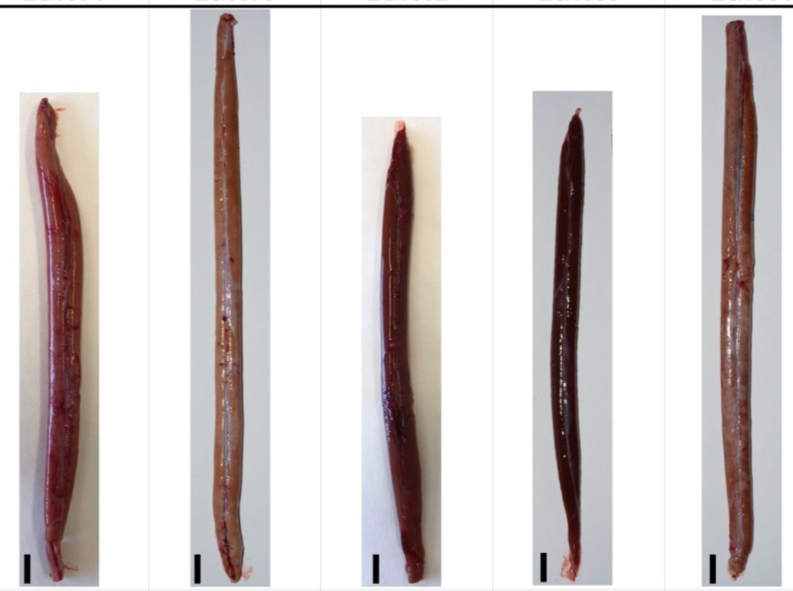				
**Sex**	female	female	male	male	female
**Fasting**	2 weeks	3 weeks	2 weeks	3 weeks	3 weeks
**Weight**	240	220	280	280	243
**Liver weight**	9.252	6.282	6.404	4.877	8.369
**Liver length**	16.31	19.2	15.34	15.51	18.79
**Heart weight**	0.406	0.318	0.391	0.357	0.343
**Oil Red O**	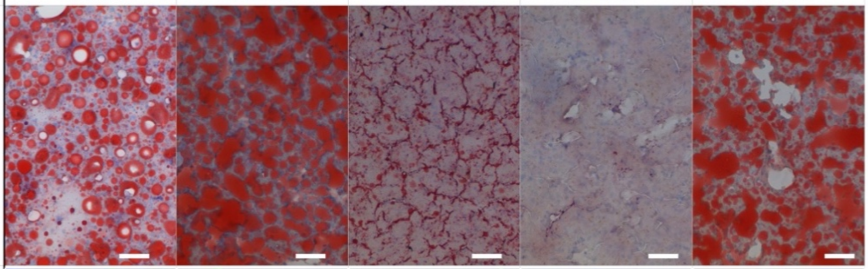				
**LAMP1**	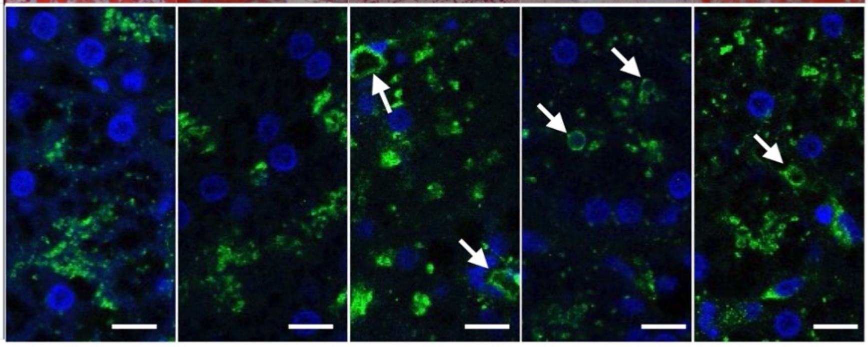				

Figure S18. Two wild type and three Lavender individuals (all animals were three-years old at sampling) were fasted for two or three weeks and weighed (g) before their liver was collected, measured and weighed (g). We weighed the heart as a comparison. Liver cryosections of each individual were stained with Oil Red O and liver paraffin sections were immunostained for LAMP1. The size (liver panels) and the lipid content (red stain with Oil Red O) of the liver varies greatly among individuals independently of the fasting period. On the other hand, all three Lavender individuals, and none of the two wild type, exhibit enlarged lysosomes (white arrows in bottom panels). Scale bars: liver 1 cm, Oil Red O 50 µm, LAMP1 100 µm.

Table S1. Corn snake genome statistics of the current assembly (for each assembly step) and of the previously published draft assemblies.

Genome assembly	Assembly length in Gb (% of gaps)	Number of sequences	N50 (Kb)	L50
***Pantherophis guttatus* v3**				
Supernova assembly	1.85 (3.2%)	41,047	30,502	18
Supernova duplicates filtering	1.69 (3.4%)	34,155	30,502	17
BioNano optical maps	2.18	3,190	881	-
BioNano hybrid scaffolding (v3)	1.71 (4.7%)	34,268	16,790	24
**Draft assemblies**				
Genome assembly v1 [1]	1.53 (3.2%)	1,781,284	3.67	94,091
Genome assembly v2 [2]	1.94 (12.5%)	114,644	1,378	279

116 **Table S2.** Types (classes and families) of repeats found in the corn snake genome assembly.

Class	Family	Masked bp	% masked
**DNA**		279,471	0.02%
	Academ-1	1,832	0.00%
	CMC-Chapaev-3	504,981	0.03%
	CMC-EnSpm	876,379	0.05%
	Crypton	11,054	0.00%
	Crypton-A	5,817	0.00%
	Crypton-I	3,400,923	0.20%
	Ginger	527,309	0.03%
	Kolobok	4,907	0.00%
	Kolobok-T2	164,027	0.01%
	MULE-MuDR	456,367	0.03%
	Maverick	139,111	0.01%
	Merlin	7,597	0.00%
	PIF-Harbinger	672,414	0.04%
	PiggyBac	4,370	0.00%
	Sola	649,214	0.04%
	TcMar	8,301	0.00%
	TcMar-Mariner	320,091	0.02%
	TcMar-Pogo	4,126	0.00%
	TcMar-Tc1	7,593,655	0.44%
	TcMar-Tc2	12,976,070	0.76%
	TcMar-Tigger	43,859,366	2.57%
	Zisupton	1,312	0.00%
	hAT	33,381	0.00%
	hAT-Ac	14,467,719	0.85%
	hAT-Blackjack	4,277,120	0.25%
	hAT-Charlie	41,804,936	2.45%
	hAT-Tag1	2,260,802	0.13%
	hAT-Tip100	22,789,321	1.34%
	hAT-hAT19	364	0.00%
	hAT-hAT6	1,554	0.00%

**LINE**		3,801,340	0.22%
	CR1	69,767,304	4.09%
	Dong-R4	10,599,957	0.62%
	I	311	0.00%
	I-Jockey	680,598	0.04%
	Jockey	857,190	0.05%
	L1	59,7813,96	3.50%
	L1-Tx1	13,095	0.00%
	L2	95,421,887	5.59%
	Penelope	17,434,423	1.02%
	R2	210	0.00%
	R2-NeSL	459	0.00%
	RTE-BovB	28,840,817	1.69%
	RTE-RTE	606,938	0.04%
	RTE-X	4,434,950	0.26%
	Rex-Babar	15,142,052	0.89%
**LTR**		3,873	0.00%
	Copia	24,439,351	1.43%
	DIRS	12,167,254	0.71%
	ERV	125	0.00%
	ERV-Foamy	133	0.00%
	ERV-Lenti	48	0.00%
	ERV1	2,286,938	0.13%
	ERV4	13,572	0.00%
	ERVK	1,339,836	0.08%
	ERVL	1,651,421	0.10%
	ERVL-MaLR	1,069	0.00%
	Gypsy	33,584,795	1.97%
	Ngaro	3,096	0.00%
	Pao	194,466	0.01%
**Other**		197	0.00%
**RC**			
	Helitron	1359132	0.08%
**Retroposon**		115	0.00%

	SVA	13,301	0.00%
**SINE**		258,085	0.02%
	5S	15,180	0.00%
	5S-Core-RTE	3,061,238	0.18%
	5S-Deu-L2	13,148	0.00%
	5S-RTE	2,643	0.00%
	5S-Sauria-RTE	41,737	0.00%
	7SL	3,219	0.00%
	Alu	846	0.00%
	B2	327	0.00%
	B4	6,401	0.00%
	Ceph	442	0.00%
	Core	892	0.00%
	Core-RTE	7,441	0.00%
	ID	10,056	0.00%
	L2	626,134	0.04%
	MIR	7,390,690	0.43%
	RTE-BovB	7,400	0.00%
	U	529	0.00%
	tRNA	89,009	0.01%
	tRNA-5S	4,884	0.00%
	tRNA-7SL	1,134	0.00%
	tRNA-CR1	186,992	0.01%
	tRNA-Ceph-RTE	17	0.00%
	tRNA-Core	6,897	0.00%
	tRNA-Core-L2	4,965	0.00%
	tRNA-Core-RTE	4,644,558	0.27%
	tRNA-Deu	11,325,979	0.66%
	tRNA-L1	7,532	0.00%
	tRNA-Meta	871,141	0.05%
	tRNA-RTE	31,074	0.00%
	tRNA-Sauria	1,073,825	0.06%
	tRNA-Sauria-L2	550,499	0.03%
	tRNA-Sauria-RTE	12,055,642	0.71%

	tRNA-V-Core-L2	101	0.00%
**Unknown**		114,766,405	6.72%
	Y-chromosome	1,836	0.00%
**Low complexity**		8,556,280	0.50%
**RNA**		60,882	0.00%
**Satellite**		3,127,955	0.18%
	Y-chromosome	251	0.00%
	acro	17,775	0.00%
	centr	143	0.00%
	macro	9,084	0.00%
**Simple_repeat**		42,401,447	2.48%
**rRNA**		35,211	0.00%
**scRNA**		936	0.00%
**snRNA**		43,172	0.00%
**srpRNA**		712	0.00%
**tRNA**		28,956	0.00%
**Total**		**753,897,242**	**44.17%**

Table S3. List of genes in the Lavender interval based on the extended mapping.

NCBI accession number scaffold	Assembly	Start	End	Gene name	BRAKER	MAKER	NCBI	Gallus NCBI	Anolis NCBI	Pseudonaja textilis	protein modification
**NW_023010793.1**	Super-Scaffold 423	39040004	39331950	RYR2	OK	OK	OK	OK	OK	OK	NO
**NW_023010793.1**	Super-Scaffold 423	39349044	39437718	MTR	OK	OK	OK	OK	OK	OK	NO
**NW_023010793.1**	Super-Scaffold 423	39441718	39503731	ACTN2	OK	OK	OK	OK	OK	OK	NO
**NW_023010793.1**	Super-Scaffold 423	39509048	39550760	HEATR1	OK	OK	OK	OK	OK	OK	YES
**NW_023010793.1**	Super-Scaffold 423	39551054	39566971	LGALS8	OK	OK	OK	OK	OK	OK	YES
**NW_023010793.1**	Super-Scaffold 423	39571552	39586995	EDARADD	NO	OK	OK	OK	OK	OK	NO
**NW_023010793.1**	Super-Scaffold 423	39605847	39625781	ERO1B	OK	OK	OK	OK	OK	OK	NO
**NW_023010793.1**	Super-Scaffold 423	39629653	39649583	GPR137B	OK	OK	OK	OK	OK	OK	YES
**NW_023010793.1**	Super-Scaffold 423	39667674	39719723	NID1	OK	OK	OK	OK	OK	OK	YES
**NW_023010793.1**	**Super-Scaffold 423**	**39748226**	**39846789**	**LYST**	**OK**	**OK**	**OK**	**OK**	**OK**	**OK**	**YES**
**NW_023010793.1**	Super-Scaffold 423	39850693	39869855	GNG4	OK	NO	OK	OK	OK	OK	NO
**NW_023010793.1**	Super-Scaffold 423	39879548	39908730	B3GALNT2	OK	OK	OK	OK	OK	OK	YES
**NW_023010793.1**	Super-Scaffold 423	39909404	39942332	TBCE	OK	OK	OK	OK	OK	OK	NO
**NW_023014704.1**	Scaffold 338387	7	6526	GGPS1	OK	OK	OK	OK	OK	NO	NO
**NW_023010793.1**	Super-Scaffold 423	39942420	40030457	ARID4B	OK	OK	OK	OK	OK	OK	NO
**NW_023010793.1**	Super-Scaffold 423	40034903	40046201	RBM34	NO	NO	OK	OK	OK	OK	NO
**NW_023010793.1**	Super-Scaffold 423	40047563	40056268	TOMM20	NO	OK	OK	OK	OK	OK	NO
**NW_023010793.1**	Super-Scaffold 423	40304249	40310164	IRF2BP2	OK	OK	OK	OK	NO	OK	NO
**NW_023010793.1**	Super-Scaffold 423	40386059	40423309	TARBP1	OK	OK	OK	OK	OK	OK	YES

**NW_023010793.1**	Super-Scaffold 423	40424006	40429205	COA6	OK	OK	OK	OK	OK	OK	NO
**NW_023010793.1**	Super-Scaffold 423	40434348	40559410	SLC35F3	OK	OK	OK	OK	OK	OK	NO
**NW_023010793.1**	Super-Scaffold 423	40628804	40646502	KCNK1	OK	OK	OK	OK	OK	OK	NO
**NW_023010793.1**	Super-Scaffold 423	40759303	40777868	MAP3K21	OK	OK	OK	OK	OK	OK	NO
**NW_023010793.1**	Super-Scaffold 423	40786191	40887401	PCNX2	OK	OK	OK	OK	OK	OK	NO
**NW_023010793.1**	Super-Scaffold 423	40902926	40911620	NTPCR	OK	OK	OK	OK	OK	OK	NO
**NW_023010793.1**	Super-Scaffold 423	40950281	40955793	MAP10	OK	OK	OK	OK	OK	OK	NO
**NW_023010793.1**	Super-Scaffold 423	41022028	41123363	SIPA1L2	OK	OK	OK	OK	OK	OK	YES
**NW_023010793.1**	Super-Scaffold 423	41346770	41507939	DISC1	OK	OK	OK	OK	OK	OK	YES
**NW_023010793.1**	Super-Scaffold 423	41514828	41531886	TSNAX	OK	OK	OK	OK	OK	OK	NO
**NW_023010793.1**	Super-Scaffold 423	41539689	41558567	EGLN1	OK	OK	OK	OK	OK	OK	NO
**NW_023010793.1**	Super-Scaffold 423	41559383	41568560	SPRTN	OK	OK	OK	OK	OK	OK	NO
**NW_023010793.1**	Super-Scaffold 423	41568647	41572380	EXOC8	OK	OK	OK	OK	OK	OK	NO
**NW_023010793.1**	Super-Scaffold 423	41590545	41603681	RHOU	OK	OK	OK	OK	OK	OK	NO
**NW_023010793.1**	Super-Scaffold 423	41809187	41825247	RAB4A	OK	OK	OK	OK	OK	OK	NO
**NW_023010793.1**	Super-Scaffold 423	41831792	41844573	CCSAP	OK	OK	OK	OK	OK	OK	NO
**NW_023010793.1**	Super-Scaffold 423	41860419	41866950	ACTA1	OK	OK	OK	OK	OK	OK	NO
**NW_023010793.1**	Super-Scaffold 423	41873368	41902725	NUP133	OK	OK	OK	OK	OK	OK	NO
**NW_023010793.1**	Super-Scaffold 423	41906208	41929592	ABCB10	OK	OK	OK	OK	OK	OK	NO
**NW_023010793.1**	Super-Scaffold 423	41934382	41945689	TAF5L	OK	OK	OK	OK	OK	OK	YES

**NW_023010793.1**	Super-Scaffold 423	41945765	41972012	URB2	OK	OK	OK	OK	OK	OK	NO
**NW_023010793.1**	Super-Scaffold 423	42120440	42194765	GALNT2	OK	OK	OK	OK	OK	OK	NO
**NW_023010793.1**	Super-Scaffold 423	42205831	42287679	PGBD5	OK	OK	OK	OK	OK	OK	NO
**NW_023010793.1**	Super-Scaffold 423	42332414	42363457	COG2	OK	OK	OK	OK	OK	OK	NO
**NW_023010793.1**	Super-Scaffold 423	42365441	42377138	AGT	OK	OK	OK	OK	OK	OK	NO
**NW_023010793.1**	Super-Scaffold 423	42380998	42425382	CAPN9	OK	OK	OK	OK	OK	OK	NO
**NW_023010793.1**	Super-Scaffold 423	42428786	42451220	CUNH1orf198	OK	NO	OK	OK	OK	OK	NO
**NW_023010793.1**	Super-Scaffold 423	42453454	42487709	TTC13	OK	OK	OK	OK	OK	OK	NO
**NW_023010793.1**	Super-Scaffold 423	42487868	42503717	ARV1	OK	OK	OK	OK	OK	OK	NO
**NW_023010793.1**	Super-Scaffold 423	42509931	42524174	FAM89A	OK	OK	OK	OK	OK	OK	NO
**NW_023010793.1**	Super-Scaffold 423	42566562	42601658	TRIM67	OK	OK	OK	OK	OK	OK	NO
**NW_023010793.1**	Super-Scaffold 423	42611074	42624890	CUNH1orf131	OK	OK	OK	OK	OK	OK	YES
**NW_023010793.1**	Super-Scaffold 423	42624904	42659910	GNPAT	OK	OK	OK	OK	OK	OK	NO
**NW_023010793.1**	Super-Scaffold 423	42664993	42670981	PDCD2	OK	OK	OK	OK	OK	OK	NO
**NW_023010793.1**	Super-Scaffold 423	42672594	42686008	TBP	OK	OK	OK	OK	OK	OK	NO
**NW_023010793.1**	Super-Scaffold 423	42688685	42697379	PSMB1	OK	OK	OK	OK	OK	OK	NO

Table S4. Genotyping for the Lavender SNP of the Lavender family individuals.

Individual	Sir	Dam	Lavender Allele	Lavender SNP
EG019			lavender/lavender	unavailable
EG036			+/+	C/C
EG284	EG019	EG036	lavender/+	C/T
EG285	EG019	EG036	lavender/+	C/T
EG286	EG019	EG036	lavender/+	C/T
EG288	EG019	EG036	lavender/+	C/T
EG431			lavender/lavender	T/T
EG432			lavender/lavender	T/T
EG469	EG431	EG284	lavender/lavender	T/T
EG470	EG431	EG284	lavender/lavender	T/T
EG471	EG431	EG284	lavender/lavender	T/T
EG472	EG431	EG284	lavender/lavender	T/T
EG473	EG431	EG284	lavender/lavender	T/T
EG499	EG431	EG284	lavender/lavender	T/T
EG807	EG431	EG284	lavender/+	C/T
EG967	EG431	EG284	lavender/lavender	T/T
EG968	EG431	EG284	lavender/+	C/T
EG969	EG431	EG284	lavender/+	C/T
EG970	EG431	EG284	lavender/lavender	T/T
EG971	EG431	EG284	lavender/+	C/T
EG972	EG431	EG284	lavender/+	C/T
EG973	EG431	EG284	lavender/+	C/T
EG1052	EG431	EG284	lavender/lavender	T/T
EG1053	EG431	EG284	lavender/lavender	T/T
EG1054	EG431	EG284	lavender/lavender	T/T
EG1055	EG431	EG284	lavender/+	C/T
EG431.284.2015.1	EG431	EG284	lavender/lavender	T/T
EG431.284.2015.2	EG431	EG284	lavender/lavender	T/T
EG431.284.2015.3	EG431	EG284	lavender/lavender	T/T
EG431.284.2015.4	EG431	EG284	lavender/+	C/T
EG431.284.2015.5	EG431	EG284	lavender/+	C/T
EG431.284.2015.6	EG431	EG284	lavender/+	C/T
EG431.284.2015.7	EG431	EG284	lavender/+	C/T
EG431.284.2015.8	EG431	EG284	lavender/+	C/T
EG431.284.2015.9	EG431	EG284	lavender/+	C/T
EG431.284.2015.10	EG431	EG284	lavender/+	C/T
EG431.284.2015.11	EG431	EG284	lavender/+	C/T
EG431.284.2015.12	EG431	EG284	lavender/+	C/T
EG431.284.2015.13	EG431	EG284	lavender/+	C/T
EG.431.284.2016.1	EG431	EG284	lavender/+	C/T
EG.431.284.2016.2	EG431	EG284	lavender/+	C/T
EG.431.284.2016.3	EG431	EG284	lavender/+	C/T
EG.431.284.2016.4	EG431	EG284	lavender/+	C/T
EG.431.284.2016.5	EG431	EG284	lavender/+	C/T
EG.431.284.2016.6	EG431	EG284	lavender/+	C/T
EG.431.284.2016.7	EG431	EG284	lavender/lavender	T/T
EG.431.284.2016.8	EG431	EG284	lavender/lavender	T/T
EG.431.284.2016.9	EG431	EG284	lavender/lavender	T/T
EG.431.284.2016.10	EG431	EG284	lavender/lavender	T/T
EG.431.284.2016.14	EG431	EG284	lavender/lavender	T/T
EG.431.284.2016.15	EG431	EG284	lavender/lavender	T/T
EG.431.284.2016.18	EG431	EG284	lavender/lavender	T/T
EG.431.284.2016.19	EG431	EG284	lavender/lavender	T/T

Table S5. Genotyping for the Lavender SNP of 71 individuals in our colony originating from 35 unrelated lineages (obtained to study different morphs and from different breeders). Lineage 35 represents 8 wild-caught individuals whose kinship is unknown. Three lineages (in bold) carry the lavender allele and individual EG339 was sequenced for the extended mapping.

Individual	Sir	Dam	Lineage	Lavender Allele	Lavender SNP
EG001			1	+/+	C/C
EG002			2	+/+	C/C
EG009			3	+/+	C/C
EG014			4	+/+	C/C
EG015			5	+/+	C/C
EG016			5	+/+	C/C
EG017			6	+/+	C/C
EG018			6	+/+	C/C
EG029			7	+/+	C/C
EG030			8	+/+	C/C
EG054	EG002	EG039	2/9	+/+	C/C
EG056			10	+/+	C/C
EG057			10	+/+	C/C
EG061			11	+/+	C/C
EG062			11	+/+	C/C
EG063			11	+/+	C/C
EG093	EG034	EG038	9	+/+	C/C
EG129			12	+/+	C/C
EG131			12	+/+	C/C
EG132			12	+/+	C/C
EG134			13	+/+	C/C
EG136			14	+/+	C/C
EG138			15	+/+	C/C
EG146			16	+/+	C/C
EG147			16	+/+	C/C
EG153			17	+/+	C/C
EG154			17	+/+	C/C
EG155			17	+/+	C/C
EG162			18	+/+	C/C
EG163			18	+/+	C/C
**EG167**			**19**	**+/+**	**C/C**
**EG196**			**19**	**lavender/+**	**C/T**
EG197			20	+/+	C/C
EG212	EG027	EG012	21	+/+	C/C
EG217	EG169	EG016	5/22	+/+	C/C
EG236	EG017	EG026	23	+/+	C/C
EG237	EG017	EG026	23	+/+	C/C
EG238	EG017	EG026	23	+/+	C/C
EG263	EG061	EG037	24	+/+	C/C
EG266	EG061	EG037	24	+/+	C/C
EG267	EG061	EG037	24	+/+	C/C
EG271			25	+/+	C/C
EG272			25	+/+	C/C
EG273			25	+/+	C/C
**EG274**			**26**	**lavender/+**	**C/T**
EG290			27	+/+	C/C
EG291			28	+/+	C/C
EG306	EG129	EG026	23	+/+	C/C
EG307	EG129	EG026	23	+/+	C/C
EG330			29	+/+	C/C
EG332			30	+/+	C/C
EG337			31	+/+	C/C
EG338			32	+/+	C/C
**EG339**			**32**	**lavender/+**	**C/T**
**EG340**			**32**	**lavender/+**	**C/T**
EG343			33	+/+	C/C

EG355	EG219	EG058	22/34	+/+	C/C
EG433			20	+/+	C/C
EG434			20	+/+	C/C
EG435			20	+/+	C/C
EG543			20	+/+	C/C
EG544			20	+/+	C/C
EG545			20	+/+	C/C
EG975			35	+/+	C/C
EG978			35	+/+	C/C
EG979			35	+/+	C/C
EG980			35	+/+	C/C
EG981			35	+/+	C/C
EG982			35	+/+	C/C
EG983			35	+/+	C/C
EG984			35	+/+	C/C

Table S6. RNA-Seq libraries used for the template transcriptome assembly. The first 14 libraries are newly sequenced for this study, while the following six come from [3, 4] and the last five were downloaded from the SRA database (accession number in parentheses). dph: days post hatching, SE: Single-end reads, PE: Paired-end reads, F: Female, M: Male, E: embryonic day.

Organ/Tissue	Sex	Stage	Library type	Total reads	Filtered reads
Brain	F	Adult	100 bp Illumina SE	34,281,246	33,457,354
Brain	M	Adult	100 bp Illumina SE	43,609,769	42,455,565
Testis	M	Adult	100 bp Illumina SE	31,078,841	30,097,637
Testis	M	0 dph	100 bp Illumina SE	28,045,260	27,382,110
Ovary	F	Adult	100 bp Illumina SE	28,480,444	27,727,494
Ovary	F	0 dph	100 bp Illumina SE	31,748,499	31,084,240
Cerebellum	F	Adult	100 bp Illumina SE	22,608,408	22,072,941
Cerebellum	M	Adult	100 bp Illumina SE	28,084,557	27,525,797
Liver	F	Adult	100 bp Illumina SE	51,573,530	49,806,445
Liver	M	Adult	100 bp Illumina SE	25,407,734	24,779,659
Kidney	F	Adult	100 bp Illumina SE	23,396,418	22,853,103
Heart	F	Adult	100 bp Illumina SE	62,410,596	61,071,345
Dorsal skin	M	Adult	100 bp Illumina SE	40,346,524	39,122,549
Ventral skin	M	Adult	100 bp Illumina SE	43,827,058	41,012,041
Testis, brain, kidney	M	Adult	100 bp Illumina PE	38,025,030	34,084,687
Testis, brain, kidney	M	Adult	100 bp Illumina PE	34,479,730	30,716,282
Embryo	-	E10/30/47	100 bp Illumina PE	33,926,691	29,734,623
Embryos	-	E10/30/47	100 bp Illumina PE	30,948,726	26,874,480
VNO	F	Adult	114 bp Illumina SE	33,472,734	29,161,853
VNO	M	Adult	114 bp Illumina SE	32,910,339	25,233,055
Skin (ERR216298)	-	Adult	100 bp Illumina PE	7,831,992	-
Salivary gland (ERR216307)	-	Adult	100 bp Illumina PE	11,857,441	-
Salivary gland (ERR216308)	-	Adult	100 bp Illumina PE	13,699,251	-
Scent gland (ERR216317)	-	Adult	100 bp Illumina PE	12,779,878	-
Scent gland (ERR216323)	-	Adult	100 bp Illumina PE	13,078,364	-

Table S7. Genomic DNA libraries used for mapping the *lavender* causative variant. In parentheses, we provide the total number of individuals in each library (column 'Source') and the average coverage for a 1.7 Gb genome (column 'Filtered reads'). PE: Paired-end reads.

Source	Genotype	Library type	Total reads	Filtered reads
Lavender male (1)	*lavender*/*lavender*	151 bp Illumina PE	235,513,391	225,147,576 (39.7x)
WT female (1)	*lavender*/+	151 bp Illumina PE	204,269,269	190,666,681 (33.7x)
Lavender offspring (20)	*lavender*/*lavender*	151 bp Illumina PE	169,455,058	155,603,453 (27.5x)
WT offspring (20)	*lavender*/+	151 bp Illumina PE	244,505,065	232,498,987 (41x)
EG266 (Genome, 1)	+/+	150 bp Illumina PE	425,172,973	359,440,755 (63.4x)
EG339 (1)	*lavender*/+	151 bp Illumina PE	175,079,918	167,770,181 (29.6x)
Wild-caught (1)	+/+	151 bp Illumina PE	178,292,101	174,411,308 (30.8x)
PG7 (1)	+/+	100 bp Illumina PE	143,620,259	127,040,500 (15x)
EG332 (1)	+/+	151 bp Illumina PE	214,263,455	200,910,331 (35.5x)

Table S8. Primers used to identify the *LYST* variant from cDNA and gDNA samples.

Primer name	Sequence	Material
LYST_8205F	CACAAAGGTGCGGGATGATG	cDNA
LYST_8851R	CACCATTCTGACGTACCCCA	cDNA
LYST_ F2	CCTTCAACGATCTCATGCAGT	gDNA
LYST_ R3	TGAATAATCAGGCAGGCAGTT	gDNA
LYST_R6	TCTCTCTTTCTCTCTCATACCTGTT	gDNA

Sequence of the corn snake wild type *LYST* mRNA. The wild type *LYST* mRNA is modelled with MAKER. The UTRs are shown in lowercase and the coding sequence is in uppercase together with the protein translation. The starting site is highlighted in green, while the stop codon is in red.

```
cggtagttggccgccgccggctccgctgtcaaagggaggaggaggaggacgaggctggcg
gaaggccgggctgagcaaggcggtttttccgcgggcggcgggagcaggtcagttgctagc
agacatactgagaacatatccatttaacgcttgataattactatcaatgagagaggaagt
ataatctgctcagacATGAGCAGTGCGAGTAACTCTCTGGCACGAGAGTTTTTGTCCGAT
                M  S  S  A  S  N  S  L  A  R  E  F  L  S  D
GTCAGCCATCTTTGCAATGCAGTGGACCAAAGGGTGGAAGCCAGGGAGGAAGAGGAAGAG
 V  S  H  L  C  N  A  V  D  Q  R  V  E  A  R  E  E  E  E  E
AAAACACATATAGCTGCCCTTGCACAATATCTTATTCAAGGTCATGGATTCATTTTGCTT
 K  T  H  I  A  A  L  A  Q  Y  L  I  Q  G  H  G  F  I  L  L
ACTAAACTTAATTCCATCATTGACCAGGAGCTTACCTGTCGAGAAGACCTCCTTACTCTC
 T  K  L  N  S  I  I  D  Q  E  L  T  C  R  E  D  L  L  T  L
CTCCTATCACTTCTGCCGCTAGTATGGAAAATACCTGTGGAGAAAGAAAAGGCCATAGAT
 L  L  S  L  L  P  L  V  W  K  I  P  V  E  K  E  K  A  I  D
TTTAATCTGCCCTTCTCTGTTGAAATATGTTTGACCAAAGGGACAAGTTCTCTGAAACCT
 F  N  L  P  F  S  V  E  I  C  L  T  K  G  T  S  S  L  K  P
ACTCAGGAAAAACAAAATATAGGAGAAAATGTTCATCTGCCTGCTCAGGCTTCTGGAAAG
 T  Q  E  K  Q  N  I  G  E  N  V  H  L  P  A  Q  A  S  G  K
CTAAATTCTTCTTGGAAGAACAGACGCCAGCGCAAAACTACTCATCGATATTCTGTGAGG
 L  N  S  S  W  K  N  R  R  Q  R  K  T  T  H  R  Y  S  V  R
GATGCAAGGAAATCCCAAATCTCCACCTCTGATTCAGAAGGCAATTCAGATGAGAAGACT
 D  A  R  K  S  Q  I  S  T  S  D  S  E  G  N  S  D  E  K  T
AGCGCTATGATAAGGCACAGGAGACCGCAGCTTCTGCAGCCTTTTCCAGCAAACCAGTCT
 S  A  M  I  R  H  R  R  P  Q  L  L  Q  P  F  P  A  N  Q  S
AAAGAGCAATCTGTAACAAGTGGATGTAACTTTCTAGAATCTGAAATGGTCCAGAGCTGT
 K  E  Q  S  V  T  S  G  C  N  F  L  E  S  E  M  V  Q  S  C
AATTATGGAAGGGAGAACTCTCAACAGTTCATTCCAGCTCAAGAAAATATTGTTCAGGCT
 N  Y  G  R  E  N  S  Q  Q  F  I  P  A  Q  E  N  I  V  Q  A
TCAGATGAGTCAGTTACACCATCTGCCAGTTATCTAGACAACTCTCCTTTTGATTTGTGT
```

S D E S V T P S A S Y L D N S P F D L C
CATGTCTTACTGTCCTTGCTGGAAAAAATATGTAAGTTTGACATTGCATTGAACCACAAG
H V L L S L L E K I C K F D I A L N H K
TCTGCATTGGCAGCCAGTGTGATAGGCACTCTAACTGAATTCTTGTCTAAACTTGGAGAT
S A L A A S V I G T L T E F L S K L G D
TGTTACAATATAAATAGTGCTGCTGAAAATGAAGCTGTTTCATCAAGTTGGACAGAAGAA
C Y N I N S A A E N E A V S S S W T E E
TCAGTTGCTCTGGTTCAGAGAATGCTCTTTAGGACGGTGCTGCATCTTATGGCAGTGGAT
S V A L V Q R M L F R T V L H L M A V D
GTTAATAATGCTGATACTATGCCCGGAAATTTGAGAAAAAATCTTATTGATTTACTTAAA
V N N A D T M P G N L R K N L I D L L K
GCAGCCTTAAAAGTGAAATGTATTCTAGATGCACTATTTAGTCCTTTTTCTTCAAGATCA
A A L K V K C I L D A L F S P F S S R S
CAAGGAACTTTACAAAACGTATTGCAGGTTAATCTTTTCTCACAGAATCGCCACAGATTC
Q G T L Q N V L Q V N L F S Q N R H R F
CTTCTTTTGCCCGAGTTCATAGAAGGAGTTCTGCAAATTCTAATCTGCTGCCTTCAGAGT
L L L P E F I E G V L Q I L I C C L Q S
GCAGCCTCTAATCCAATTTATTTTAGCCAAGCAATGGATCTGGTTCACGAATTCATACAG
A A S N P I Y F S Q A M D L V H E F I Q
CAGCAGGGTTTCAAACTGTTTGAAACCACAGCACTCCAGATGGAAGGATTATGCGCCAGA
Q Q G F K L F E T T A L Q M E G L C A R
GGCCCAGAGGTCAACACAGAAGCGTCAGAGTATCTCAAAGCCCTCATCAACAGCATTATG
G P E V N T E A S E Y L K A L I N S I M
AAAATAATCAGCACTATCAAAAAAGTGAAATCGGAACAACTTCATCAGTCTGTATGTACG
K I I S T I K K V K S E Q L H Q S V C T
AGAAAACGGCACAGGCGATGTGAGTATTCTCACTTCATGCATCACCACAGAGATCTCTCA
R K R H R R C E Y S H F M H H H R D L S
GGATTGCCAGTTTCTACTTTTAAAAATCAAGCTTCTAAGAGTCCTTTTGAAGAAACAGCT
G L P V S T F K N Q A S K S P F E E T A
GATGGGGAAGTTCATTATCCTGACCGGTGTTGCTGCGTTGCTGTTTGTGCTCACCAGTGT
D G E V H Y P D R C C C V A V C A H Q C

CTGAGGTTGCTGCAAAAACTTTCTCTGAACAGTACATGTTTTCAGATCTTAACTGGAATC
L R L L Q K L S L N S T C F Q I L T G I
CATAATGTAGGCATATGTTGTTGCATGGATCCTAAATCAGTAATTAGCCCCTTGCTTCAT
H N V G I C C C M D P K S V I S P L L H
TCCTTCAGATCTCAAACATTCAAGAATTTTCAGCCACATATATTAAGCATCCTTAATAAG
S F R S Q T F K N F Q P H I L S I L N K
TTTATTTTAGAACAACTTGGAGGATTACAAATTTCTCAGAGAGTGACACATGCCTCTTGC
F I L E Q L G G L Q I S Q R V T H A S C
AACATCTGTAGCATTGACTGTGATCAACTTGCAGAACTTGATGACTTCCTACATGGGAAT
N I C S I D C D Q L A E L D D F L H G N
GCTGCTGAAGTATCAGTGTCAAGTTCATCAGCTCCATACAGGTTTCAAGGAATTTTGCCT
A A E V S V S S S S A P Y R F Q G I L P
AACAGAGGATCTGAAAATATGTTTCTGAAATGGGATGCCTTGGAGGCATATCAGAGCCTT
N R G S E N M F L K W D A L E A Y Q S L
GTTTTTGAGGATGATGACAAACTGCGTTGCATGCAGATTGCTAGTCATATTTGCAGTTTG
V F E D D D K L R C M Q I A S H I C S L
ATCCAAAAAGGCAATGAGATTATTCAGTGGAAACTATACAATTATATATTCAGCCCAGTG
I Q K G N E I I Q W K L Y N Y I F S P V
CTCCAGAGAGGTGTTGAGTTGGCTCATTACTCCCAGCAAGCTGGTGCAACTACTGCTTTT
L Q R G V E L A H Y S Q Q A G A T T A F
AGTCAAACAAGTGGCTATCAAAAAAGATGTCTGCCTCAGGAAGTCCTTCAGATCTATTTA
S Q T S G Y Q K R C L P Q E V L Q I Y L
CAGACTCTACCAATACTGTTTAAATCCAGAATAATACAAGAATTATTTTTAAGCTGTAAT
Q T L P I L F K S R I I Q E L F L S C N
GGAATAAATCAAATAACTGAATTAAATTACTTGGACAGTGTGAGAGCATACTCATTGAAA
G I N Q I T E L N Y L D S V R A Y S L K
GTATTTGAAACATTAATATTTTCCCTTGGAGATCAGCAGACAGATCAATTGATGCCAGAT
V F E T L I F S L G D Q Q T D Q L M P D
GTGGATGGTTTGGACAGTGAAGAAAAAGTGCCTGCCTTAGACTTGGATGTTTCTGTTCAC
V D G L D S E E K V P A L D L D V S V H
AGACAGCAAATGGTTTCCGATGTGCCTCAGAGCCTAAGTAAATTTTATGCCGGGCTCAAA

R Q Q M V S D V P Q S L S K F Y A G L K

GAAGTGAATCCGAAAAGGAAGAAATCAGTCAGTCAAGATGTTCACCTCAATATGATAAAT

E V N P K R K K S V S Q D V H L N M I N

CTATTTCTCTGTGTTACTTTTTTATGCGTAAGTAAGGAAGCAGAGGCTGACCGAGATTCA

L F L C V T F L C V S K E A E A D R D S

ACAAATGATTCTGAAGATACTTCAGGTTATGATAGTACTGCTAGTGAACCTTTCAGCCAT

T N D S E D T S G Y D S T A S E P F S H

AAGCTTCCATGTCTGTCATTTGAAAGTTTGACTCTACCATCCTTGGAACATATACACAGG

K L P C L S F E S L T L P S L E H I H R

GCAGCAGATACTTGGTCAATGTGTCGTTCGATCTATTTGTGTAATTCAATATTCCAAAGA

A A D T W S M C R S I Y L C N S I F Q R

CAATTCCATAGACTTGGCGGTTTTGAGGTATGTCACAGATTAATAGCCATGATTATTGAG

Q F H R L G G F E V C H R L I A M I I E

AAACTTGGCGTGAAAGAAGAAAAGGAAGAAGGTATGCAGGAGAAACCTACTGAATTTCTT

K L G V K E E K E E G M Q E K P T E F L

TTCAAAAATGGCTCATCGCAGTTGATAATAAACAATGGGAAAGGGCAGTCGGACAATAAT

F K N G S S Q L I I N N G K G Q S D N N

ACTGATGGAAATCTTAACAATATAGATCAACAGAAATCAATCTCTTATCAGCATTCAGAT

T D G N L N N I D Q Q K S I S Y Q H S D

GCAGAGCAAGCTTTGACTGATGGCACTGCAAAATCAAAACATGTCTTAATGCAAGAAAAT

A E Q A L T D G T A K S K H V L M Q E N

GACTGGTCGCTGCAAGGTATAAGACTCTTGGAAGCTCTGCTGGCAATTTGTTTACACAGT

D W S L Q G I R L L E A L L A I C L H S

GCAACTAATTTCCAGCAGAAGGCTGAAATGGAGTCTCTTCAGAACATTTGTGTGGAAGAT

A T N F Q Q K A E M E S L Q N I C V E D

ATATTACTTGAAATGAGGGAACAGCTTACTAAAGCTAGAGTAATGGAAACAGAGTTGGTG

I L L E M R E Q L T K A R V M E T E L V

AAGCCTTTATTTGATTCTTTGCTTCGAATTGCATTGGGAACATATTTTACTGATCTGGAT

K P L F D S L L R I A L G T Y F T D L D

CGTTCAGAAGAAAAGAAAGAAAAGACCCATCAGTCTGAAAAGGAGCCTGTGTCTCGGCCT

R S E E K K E K T H Q S E K E P V S R P

```
GATGAAATATCTGAAGAAGCTGATGAATCACAATGCTATTGCTTTAAATTTTTTGCTGAA
 D  E  I  S  E  E  A  D  E  S  Q  C  Y  C  F  K  F  F  A  E
GAAAAAGGCTATGATGCTGATAGTGAAAGTAATTCAGATGACAATGAAAGCAAGGATGAA
 E  K  G  Y  D  A  D  S  E  S  N  S  D  D  N  E  S  K  D  E
GGAACAGACATTAAAAGAGAGCAAGTATGTTTTGGCTATTGCAGTGTTCCTAGTGATCTT
 G  T  D  I  K  R  E  Q  V  C  F  G  Y  C  S  V  P  S  D  L
GCTGAAAATATTATTCAAGGAGAATTGATATATCCTGAACTATGTATGCTGGAATTACAT
 A  E  N  I  I  Q  G  E  L  I  Y  P  E  L  C  M  L  E  L  H
TTACTTTCCGTTGGCAGTCCGAGCCTAGATGTGCTCACTCATGTTTTCCACAGCTTCTTG
 L  L  S  V  G  S  P  S  L  D  V  L  T  H  V  F  H  S  F  L
AAAGTAGTCAGGCTGAAAGAAAAAAATGCAACATTACTAATGGAACAGGGAGCTGTAAAG
 K  V  V  R  L  K  E  K  N  A  T  L  L  M  E  Q  G  A  V  K
AGCCTTCTGGGAGGATTCCTCCATGTATTGACACAAACAGATTCCTGTTTTCATGAATGT
 S  L  L  G  G  F  L  H  V  L  T  Q  T  D  S  C  F  H  E  C
CAGAAAATATTAGTGGATCTGCTGGTTTCTCTGATGAGCTCAAGCACATCTTCAGAAGAG
 Q  K  I  L  V  D  L  L  V  S  L  M  S  S  S  T  S  S  E  E
CTCACACTTCTTCTAAGGATCTTTTTAGAAAAAACACCTGCTACAGGTATTCTCCTTAAA
 L  T  L  L  L  R  I  F  L  E  K  T  P  A  T  G  I  L  L  K
GGAATCCTAAAGATTTTGGAGGCAAATACCAGGATGAGGCCTTTACAGCATATCGTATTT
 G  I  L  K  I  L  E  A  N  T  R  M  R  P  L  Q  H  I  V  F
CCTTTGCGCCAAATTCCAAGTTTAGGAAGCAGCCCTTGCCAAAAATCAGCCCCTACATTC
 P  L  R  Q  I  P  S  L  G  S  S  P  C  Q  K  S  A  P  T  F
GGCAGCAAAACTGTAGCATTACTAAGGCGAGCAAAATTAGTCCAGTCAAAGAAAGAACCA
 G  S  K  T  V  A  L  L  R  R  A  K  L  V  Q  S  K  K  E  P
GAGGGTGAGAGTTTGCCACACCATCTTCTCTCTCCATGGCATGTGGCTCCAATCCATTTA
 E  G  E  S  L  P  H  H  L  L  S  P  W  H  V  A  P  I  H  L
CCTCTGGTGGGACAAAACTGCTGGCCACATATGTCTGAAGGATTCAGTATCTCGTTGTGG
 P  L  V  G  Q  N  C  W  P  H  M  S  E  G  F  S  I  S  L  W
TTTAGTGCTGAATGTATTCTCAGAGGAAGCAGTTCCTTGGAGAAAGGCAAAAAAATGAAA
 F  S  A  E  C  I  L  R  G  S  S  S  L  E  K  G  K  K  M  K
AGAAGGAGCAAGTCACTAATATCACATGAAAGCAGTTTTGATGAAGCAGAAGAAAGCAAA
```

R R S K S L I S H E S S F D E A E E S K
CCTGGACTTGATTACCTCAATTTGGGAGACAAACTTCTTGAAGATGGCAACCTTCATATA
P G L D Y L N L G D K L L E D G N L H I
TTTTCACTGGGCTCCAAAGCATTGATGATACAAGTCTGGGCTAATGTGAACTCAGGAGCT
F S L G S K A L M I Q V W A N V N S G A
TTCACATTTCGTATGTGTATGGATCCAAATGATGAAATGAAAGCTGGATTATTAGCACAA
F T F R M C M D P N D E M K A G L L A Q
GCTGAAACACCAGAGAATACCTTCCTTGTAGGCAAATGGCAGCATTTGGCCCTAACCTAC
A E T P E N T F L V G K W Q H L A L T Y
ACTGAACAACCAGAAAACAAGAAAAATATCCATGGAACTCTCTCTTTATGGATTTGTGGT
T E Q P E N K K N I H G T L S L W I C G
CAGAGAAAACCTGACATTTGTTTAGATTATACACTTCCAAGAAAAATGAGTTTGTCATCC
Q R K P D I C L D Y T L P R K M S L S S
GACTCTAATAAGACCTTCTGCATGACAGGCCATTATACATCATCCCAAGATGATTTTTTG
D S N K T F C M T G H Y T S S Q D D F L
CAATTGACTGGGAGGTGGAGCCTTGGAAATTTGCTTCTCTTCAATGGTGCAACAGTGGGT
Q L T G R W S L G N L L L F N G A T V G
CCTGAAGAGGCATTTTATCTGTATGCTTGTGGGCCAGATTTCACGTCTGTAATGCCATGT
P E E A F Y L Y A C G P D F T S V M P C
AAATATGGCAAGTCAATACCTAATTACTCCAAATATATAGATAAAGAGATTCTACAGTGT
K Y G K S I P N Y S K Y I D K E I L Q C
GAACAAATCAAAGAACTTCTGTTGACAAAAAAAGAAGTAGACACTGGGCCTTTAATTGAA
E Q I K E L L L T K K E V D T G P L I E
AACCTTGCTACAGTATACACCACTTGCTGCCCTGGTTACTACACAATCTATGAGCCAGTA
N L A T V Y T T C C P G Y Y T I Y E P V
ATCAGACTTAAAGGTCAAGCGAAAACCATTCCTTCTCAACGACCTTTCAGTTCAAAAGAA
I R L K G Q A K T I P S Q R P F S S K E
GTACAAAGCAATTTATCAGATCCTCATTATCTGAAAATGCTGCAGCCTGCCAAATACAAA
V Q S N L S D P H Y L K M L Q P A K Y K
GGTCTCCAAGGCATTCTTCATGAGATTGGTGGAATTGGGTCATTTGTCTTCCTTTTTGCC
G L Q G I L H E I G G I G S F V F L F A

AGAGTTGTAGAGATCAGCACTTGTGAAGAAACACAAGCTTTAGCTCTGAAAATTATCCTT

R V V E I S T C E E T Q A L A L K I I L

GTTTTAACAAAACACAATCAGCAGAGAATACATGAGTTAGAAAACTGCAATGGTTATTCA

V L T K H N Q Q R I H E L E N C N G Y S

ATGATTCATCAGGTGTTGATAAAGTCAAAGTGTATTGTTGGATTTCACATTTTGCAGACT

M I H Q V L I K S K C I V G F H I L Q T

CTTCTCGAAGGATGCTGTAATGAGAAAATGATTCATCAAGATGACAATGGACAATTCATG

L L E G C C N E K M I H Q D D N G Q F M

CTTGATGTGGAATCCAGAGCAATAATCCAGGATGTAAAATTGTTGGAAGAGCTACTGTTT

L D V E S R A I I Q D V K L L E E L L F

GATTGGAAGATATGGTCTAAAGCCCAGCAAGGTGTGTGGAAAACACTGTTATCAGCTTTA

D W K I W S K A Q Q G V W K T L L S A L

GAAACACTTATCCAAGATAATCACTGGCAACAGCAATTTAACATTAAACAACTTCTTAAA

E T L I Q D N H W Q Q Q F N I K Q L L K

GCTCGAGTTGTGCATCATTTCTTGAGGACATGTCAAGTTCTGCAGGAGCACAAAGAAGAA

A R V V H H F L R T C Q V L Q E H K E E

CATTTCACATCTATTCCTTCAGAAGTTTCTACTTCATTTGTGAAAATAATTGAAGAAGTG

H F T S I P S E V S T S F V K I I E E V

CTTGGATCTCCACCAGATCTGGAACTGCTGACCCTGATCTTCAATTTCCTTGTAGCAGTT

L G S P P D L E L L T L I F N F L V A V

CACCCTGCTACTAATACATATGTTTGTCACAATCCCAGAGATTTCTATTTTTCCTTGCAT

H P A T N T Y V C H N P R D F Y F S L H

ATAGATGGCAATATTTTTCAAGAGAAGGTTCAATCCATCATGTATATGAGAAATTCCAGT

I D G N I F Q E K V Q S I M Y M R N S S

AGTGGTGGAAAATCTGTTGACAGTCCAGTTTTAGCTATGATGAGCCCAGCTAGATTTTCT

S G G K S V D S P V L A M M S P A R F S

GCTGCCCAGCAAGGAAATGATTATTCCGAAGCAAGCAACTCTCAAAACACAGTTCATCAA

A A Q Q G N D Y S E A S N S Q N T V H Q

GCACCCGAAAGGCTCTGCAAAAGCTTACCATCATCTCCTATATCGTCCCCAGGAATGCAT

A P E R L C K S L P S S P I S S P G M H

CGGGGTTTTTTTAGGAATATAGGCTGGAGTGTTGAGAATCTTCATGCAGTTACACAGCTT

R G F F R N I G W S V E N L H A V T Q L
AAAAGAGTTGAGCCACTCCAGAAAAACAATTTGATAAAGCATACAGAAGCCTTCAAAAGG
K R V E P L Q K N N L I K H T E A F K R
AATGAGGAAGAACATTTCATTAGTAGTTGTGAATCTGCAAAAACAATTGTAGGATTGGGA
N E E E H F I S S C E S A K T I V G L G
GAAGTAATCCCTGCAGAAACACTGGGTATTTCAAGAGAAGAGAGAGAATCATTAGATAAA
E V I P A E T L G I S R E E R E S L D K
ACCTCTGAGAATCATGAAACAGTCCCAGGAGTGAAACCCAGTGAATATAATGTTATTCCT
T S E N H E T V P G V K P S E Y N V I P
GAGTTATCTCTGAAGCGGCCTGATAATCTGAAGGGGTTGTCTCCTATACAGAAAAGCCAT
E L S L K R P D N L K G L S P I Q K S H
GGCAATTTTGCAGGCTTGGGGTTAGCCTTCTCCATTCACACTGGAGCTACTGTACATCGC
G N F A G L G L A F S I H T G A T V H R
TGGCCAAGCTATAAGAATGTTCCTTCAGAAGATTGGGAACATCTCACCTTGTCTTCAGCT
W P S Y K N V P S E D W E H L T L S S A
AACGAATGGGCTCCACAAAAATTTGAAAGCATGTCTAATAGATCAACAGAAGATTGTCTC
N E W A P Q K F E S M S N R S T E D C L
GTACTCATATGCCATGGTCTTTATCATCTTCTGCGAAGAATTCTTTTAATTTTGCCAGAT
V L I C H G L Y H L L R R I L L I L P D
GTGATGCTCCCAGATGTATTGGACAAGCTGATTCAGCCAGACATTCTCATAGTTCTTGTG
V M L P D V L D K L I Q P D I L I V L V
AACCATCCATCTACTCTCATTCAGCAAGGAGTCATTAAACTTTTGAACACCTATTTCAGC
N H P S T L I Q Q G V I K L L N T Y F S
AGAGCAACTAGAGAACAAAAAGAGAAGTTTTTGAAGAACCGAGGTTTCTCCTTGTTAGCT
R A T R E Q K E K F L K N R G F S L L A
AATCAGCTGTATCTTCACCAAGGAACACAGGAAGTTATGGAATGCTTTCTAGAAATGCTT
N Q L Y L H Q G T Q E V M E C F L E M L
TTTGGTCGATCGGTTGGCCTGGATGAAGACTTTGATCTAGAAGATGTGAAAAATGCTGGG
F G R S V G L D E D F D L E D V K N A G
CATTTTCAAAAGTGGTGTGTCATTCCTGTTCTGGGCCTTATTGAGAATTCCCTCCATGAT
H F Q K W C V I P V L G L I E N S L H D

TGCATCCTGTTACATAACTCCTTTTGTTTCCTGCTGCAAATCATAAATTCTTGTCCTAAA
C I L L H N S F C F L L Q I I N S C P K
ATGGCAGACATGCTGTTGGATAATGGCTTGTTGTATGTGCTCTGTAATACTTTAGCAACA
M A D M L L D N G L L Y V L C N T L A T
CTTAATGGACTTGAAGCCAACATTCTCTCAAATGACTCCAGATTACTTGTGTATGATATT
L N G L E A N I L S N D S R L L V Y D I
CAGCAGTTACTTGCAGCAGTGACAATCCATGCTTGCAGCTGTTCAGGAACACAATATTTT
Q Q L L A A V T I H A C S C S G T Q Y F
CGGATTATTGAAGATCTTATGGTGTTACTTGGACATCTGCAAATGAGCAAGAATGACAGA
R I I E D L M V L L G H L Q M S K N D R
ACACAGAAAATGGCAGTTAATTTGCAATTCACAGTCTTGCAATCAGCTGTAAAGTATATA
T Q K M A V N L Q F T V L Q S A V K Y I
AAAACTGTAGGAAATCCAGATTCAGAGCATTTAAGCCATTCACTCCCTTTATCATTTGAA
K T V G N P D S E H L S H S L P L S F E
GATCATCATGCTATATTTCAAAAACATAAAAGCATCGCTGCACCAAGAAAATTTTCTATT
D H H A I F Q K H K S I A A P R K F S I
GCTCAGCCTGAGACATTCCTAATGAAAATGCATACAATTGCAAACGAGGCACTGGATTTT
A Q P E T F L M K M H T I A N E A L D F
ATGATGCTGCGTAGAATGAATCAAGACAGTTCTCTTTGTGCCACTGAAGCAGAGCTTATA
M M L R R M N Q D S S L C A T E A E L I
CAGAGGTTACAGAGGATTACTGTACTAACCACCAATAGATTCATGTATCAAGACCTTTCT
Q R L Q R I T V L T T N R F M Y Q D L S
GAAAATTGTCCTGAAATGATTGAGATCCCATTCCAGAATAGATCTTCCAGAATAGTTTCC
E N C P E M I E I P F Q N R S S R I V S
CAACAGGACAAGACTGAAAAGGAGGATGAACAGAAAGAACTTATCCCAAGAAACACTTTC
Q Q D K T E K E D E Q K E L I P R N T F
TTGAGAGAAATGTTTAAAATAATGATTGAAGGAATCGGATTATCTATTGGCACTTCCAGA
L R E M F K I M I E G I G L S I G T S R
TTAAGCACCCCCAAGCAACAGTGGAAGAAAATTCTCTTGTCTTGTAAGGATACTTTTCGA
L S T P K Q Q W K K I L L S C K D T F R
GTGCAGCTTGGGCGACTGCTTGTGCATGCTTTGTCTCCAACACGCTCTTTGCAAGAACGA

V Q L G R L L V H A L S P T R S L Q E R
AAGCACACTTTGGAATTCTTACATGAAATTAACCACCAGGAGATTTTGCGAGAGTGTTTA
K H T L E F L H E I N H Q E I L R E C L
AGCACAACTTTGCAGCATGGGCCTAAGTTGTCACTGTATTTATTTGAATTAATGCATGAT
S T T L Q H G P K L S L Y L F E L M H D
CATAAAGATGAATTAACCAAAGAAGAACAGCTATCGGTCGGAGCATTCATGAACACATTA
H K D E L T K E E Q L S V G A F M N T L
AAGCTTTGTGGGTACAAATACATTCCTCTCAATGCTCCACCGAAACCTGACCTCATAAAA
K L C G Y K Y I P L N A P P K P D L I K
GCAATGGAAGAGGACCATAAGAAATACCTGGGCGAAGAAGAGGTGAATAAAGCTGCTTGG
A M E E D H K K Y L G E E E V N K A A W
GAAAAGATAATGGCCAACAACTGGCAAACTCTATTTCAGCGCTTGGATGCAAAGTCAAAG
E K I M A N N W Q T L F Q R L D A K S K
GAGATTTCTAAAATTGCAGGGGACATCACTCAGGCTGCCTCTCTGTCCCAGGGAATGGAA
E I S K I A G D I T Q A A S L S Q G M E
AGAAAGAAGGTGATTCAGCACATCAGGGGGATGTATAAAACAGACTTAAGTGCCAGCAGA
R K K V I Q H I R G M Y K T D L S A S R
CACTGGCAGGAGCTCATTCAGCAGTTTACACATGATAGGGCTCTTTGGTATGACCCTGCC
H W Q E L I Q Q F T H D R A L W Y D P A
TCCTACCCAACATCTTGGCAGCTGGACCCAACCGAAGGACCTAACAGAGAGAGACGCCGC
S Y P T S W Q L D P T E G P N R E R R R
TTGCAGAGGTGCTACTTAACAATTCCAAATAAATACCTCCTCAAAGACAGGCAAAAATCG
L Q R C Y L T I P N K Y L L K D R Q K S
GAAGAGGTTCTTAAGCCCCCTCTGGCCTATCTGTTTGAAGACAAAACACATTCATCTCAC
E E V L K P P L A Y L F E D K T H S S H
TCTTCTACAGTGAAAGACAAAGCTGCCAGTGAGCATATAAGAGTTAATCGAAGATGTATA
S S T V K D K A A S E H I R V N R R C I
AATGTGGCCCCCATCTAGAGAGACCCCTGGTGAATTGTTACTGGGCAAATGTGGAATGTAT
N V A P S R E T P G E L L L G K C G M Y
TTTGTGGAAGACAATGCTTCTGAAATTATTGAAAATTCAAGTTTTCAGGGAGAAACTGAG
F V E D N A S E I I E N S S F Q G E T E

CCGGCATCATTTTCTTGGATATTTGAAGAAATCAAAGAAGTGCACAAGCGTTGGTGGCAG

P A S F S W I F E E I K E V H K R W W Q

TTAAGAGATAATGCTGTAGAAATATTTTTAACAAATGGCAGAACGTTACTATTAGCATTT

L R D N A V E I F L T N G R T L L L A F

GACAACACAAAGGTGCGGGATGATGTGTACCACAATATCCTAACAAACAATCTTCCTAAT

D N T K V R D D V Y H N I L T N N L P N

CTCTTGGAGTATGGCAACATTACTGCATTAACCCATCTGTGGTATACTGGACAGATCACT

L L E Y G N I T A L T H L W Y T G Q I T

AATTTTGAATACCTCACTCATTTAAATAAACATGCTGGCCGTTCCTTCAACGATCTCATG

N F E Y L T H L N K H A G R S F N D L M

CAGTATCCAGTATTTCCCTTTATACTTTCCGACTATACTAGTGAAACATTGGACTTAAAT

Q Y P V F P F I L S D Y T S E T L D L N

GACCCATCTGTTTATAGAAATTTGATCAAGCCAATAGCTGTACAGTCAAAAGAAAAGGAA

D P S V Y R N L I K P I A V Q S K E K E

GATCGTTATGTGGACACTTACAAGTACCTAGAAGAAGAATATCGTAAAGGAGCTAGAGAA

D R Y V D T Y K Y L E E E Y R K G A R E

GATGATCCAATGCCTCCTGTACAACCATATCATTATGGTTCTCATTATTCAAATAGTGGA

D D P M P P V Q P Y H Y G S H Y S N S G

ACAGTTCTTCACTTTCTGGTTAGAATGCCTCCTTTCACAAAGATGTTTTTAGCTTATCAA

T V L H F L V R M P P F T K M F L A Y Q

GATCAGAGTTTTGATATTCCAGACAGAACTTTCCATTCTACTAACACAACCTGGCGCCTT

D Q S F D I P D R T F H S T N T T W R L

TCATCATACGAATCCATGACTGATGTAAAGGAACTCATCCCAGAATTTTTTTATCTTCCT

S S Y E S M T D V K E L I P E F F Y L P

GAATTTTTAGTTAACAGGGAAGGTTTTGATTTTGGGGTACGTCAGAATGGTGAAAGAGTA

E F L V N R E G F D F G V R Q N G E R V

AACCACGTGAACCTCCCACCCTGGGCTCGAAATGATCCAAGATTATTTATCCTGATCCAT

N H V N L P P W A R N D P R L F I L I H

CGTCAAGCTTTAGAGTCTGACCATGTTTCCCAAACCATCTGCCATTGGATTGATTTAGTA

R Q A L E S D H V S Q T I C H W I D L V

TTTGGGTATAAACAAAAAGGCAAGGCCTCCGTTCAAGCTATTAATGTCTTTCATCCTGCT

F G Y K Q K G K A S V Q A I N V F H P A

ACATATTTTGGAATGGACGTGTCAGCTGTTGAAGATCCAGTCCAAAGAAGAGCTCTGGAG

T Y F G M D V S A V E D P V Q R R A L E

ACCATGATAAAAACATATGGGCAGACTCCTCGACAGCTGTTCCACACTGCCCACGTCAGC

T M I K T Y G Q T P R Q L F H T A H V S

AGATCGAAACCTATGGAAGGAGAACTCCCTGCTGCAATGGGATTATTAGTGCAGTTTGCG

R S K P M E G E L P A A M G L L V Q F A

TTCAGAGAAACAAGGGAGCATACGAAAGAGATTGTCTATCCAAGTCCTTTACCTTGGATC

F R E T R E H T K E I V Y P S P L P W I

AGAGGCTTAAAATGGGGGGAGTACGTAGGTTCTCCCAGTGCCCCTGATCCACTTGTCTGC

R G L K W G E Y V G S P S A P D P L V C

TTTAGCCAGCCACATGGAGAAAGATTTGGCTCCTTGCAGGCTTTACCAACTAGAGCAATT

F S Q P H G E R F G S L Q A L P T R A I

TGTGGTTTGTCCCGAAAGTTCTGCCTTCTGATGATCTATAGCAAAGAGCAAGGTGTTAGA

C G L S R K F C L L M I Y S K E Q G V R

AGCATGCATAGCACAGATATCCAGTGGTCAGCCATCCTGAGTTGGGGATATGCAGATAAT

S M H S T D I Q W S A I L S W G Y A D N

ATTTTAAGATTAAAAAGCAAGCAAAGTGAACCTCCAGTAAATTTTATACAGAGTTCACAG

I L R L K S K Q S E P P V N F I Q S S Q

TTTCACCAGGTAACAAGTTGTGCTTGGGTGCCAGACAGCTGCCAACTTTTTACTGGAAGT

F H Q V T S C A W V P D S C Q L F T G S

AAATGTGGAGTTATCACAGCGTATATCAACAGATTCACTAGTAGCACTCCTTCTGAAATC

K C G V I T A Y I N R F T S S T P S E I

GACATGGAGTCTCAGATGCATCTATATGGACACACTGCAGAGATCACCAGCTTGTTCGTC

D M E S Q M H L Y G H T A E I T S L F V

TGCAAACCCTATAGCATCATGATAAGTGTGAGCAAAGATGGAACCTGTATCATATGGGAT

C K P Y S I M I S V S K D G T C I I W D

TTGAACAGGCTCTGCTATGTGCAAAGTCTTGCTGGGCACAAAAGTCCTGTTACGGCAGTG

L N R L C Y V Q S L A G H K S P V T A V

TCTGCTAGTGAAACAACAGGGGACATTGCAACTGTTTGCGATTCAGTTGGTGGTGGTAGT

S A S E T T G D I A T V C D S V G G G S

```
GACCTCAGACTCTGGACAGTCAATGGTGACCTGGTGGGACATGTGCATTGCAGAGAAATC
 D  L  R  L  W  T  V  N  G  D  L  V  G  H  V  H  C  R  E  I
ATATGCTCTGTTGCTTTCTCCAACCAGCCTGAAGGAATCTCTATCAATGTGATTGCTGGA
 I  C  S  V  A  F  S  N  Q  P  E  G  I  S  I  N  V  I  A  G
GGGCTAGAAAATGGAGTTGTGAGATTGTGGAGCACATGGGATTTGAAACCTGTCCGAGAA
 G  L  E  N  G  V  V  R  L  W  S  T  W  D  L  K  P  V  R  E
ATTACATTTTCTAAATCAAATAAACCAATTGTCAGCCTTACCTTTTCGTGTGATGGCCAT
 I  T  F  S  K  S  N  K  P  I  V  S  L  T  F  S  C  D  G  H
CATTTGTACACCGCAAATAACGAAGGAACGGTGATCTCTTGGTGTCGCAAGGACCAACAG
 H  L  Y  T  A  N  N  E  G  T  V  I  S  W  C  R  K  D  Q  Q
CGCTTGAAGCTTCCTATGTTCTACTCATTCCTCAGCAGCTATGCAGCTGGTTGAcagctc
 R  L  K  L  P  M  F  Y  S  F  L  S  S  Y  A  A  G  *
tgtcggcttctcagctttggtaaattatgaagcactttcagtacagagtggactgtgaag
cctctttggttttgctagattttgaagtacatgtatgactttatcatcagcaagtagacc
tttgatgacgtcaacgaatggctgctacgtatgctcatgcaccaaatttatgaaccatat
cacagtgataagaaaattctggcttgccttttttgggtaccaaaaggacactaagggcgct
aattcagaagtatatatgctgcaatatgttatgtaacataaacatattttatggatact
ttcctggacatacatattctaaatcttcaagcattagtgataacttttgctcatctgtta
tgtaaaaatataattaaaatatttcaaaggtttatataaaaaccagtgtttgtgtttaat
tcattattagattataatccaccttgttcttctatacatataaccatctcaaat
```

Sequence of the Lavender corn snake *LYST* mRNA. The UTRs are shown in lowercase and the coding sequence is in uppercase together with the protein translation. The starting site is highlighted in green, the stop codon in the wild type is in red and the single nucleotide substitution introducing an early stop codon is highlighted.

```
cggtagttggccgccgccggctccgctgtcaaagggaggaggaggaggacgaggctggcg
gaaggccgggctgagcaaggcggtttttccgcgggcggcgggagcaggtcagttgctagc
agacatactgagaacatatccatttaacgcttgataattactatcaatgagagaggaagt
ataatctgctcagacATGAGCAGTGCGAGTAACTCTCTGGCACGAGAGTTTTTGTCCGAT
                M  S  S  A  S  N  S  L  A  R  E  F  L  S  D
GTCAGCCATCTTTGCAATGCAGTGGACCAAAGGGTGGAAGCCAGGGAGGAAGAGGAAGAG
 V  S  H  L  C  N  A  V  D  Q  R  V  E  A  R  E  E  E  E  E
AAAACACATATAGCTGCCCTTGCACAATATCTTATTCAAGGTCATGGATTCATTTTGCTT
 K  T  H  I  A  A  L  A  Q  Y  L  I  Q  G  H  G  F  I  L  L
ACTAAACTTAATTCCATCATTGACCAGGAGCTTACCTGTCGAGAAGACCTCCTTACTCTC
 T  K  L  N  S  I  I  D  Q  E  L  T  C  R  E  D  L  L  T  L
CTCCTATCACTTCTGCCGCTAGTATGGAAAATACCTGTGGAGAAAGAAAAGGCCATAGAT
 L  L  S  L  L  P  L  V  W  K  I  P  V  E  K  E  K  A  I  D
TTTAATCTGCCCTTCTCTGTTGAAATATGTTTGACCAAAGGGACAAGTTCTCTGAAACCT
 F  N  L  P  F  S  V  E  I  C  L  T  K  G  T  S  S  L  K  P
ACTCAGGAAAAACAAAATATAGGAGAAAATGTTCATCTGCCTGCTCAGGCTTCTGGAAAG
 T  Q  E  K  Q  N  I  G  E  N  V  H  L  P  A  Q  A  S  G  K
CTAAATTCTTCTTGGAAGAACAGACGCCAGCGCAAAACTACTCATCGATATTCTGTGAGG
 L  N  S  S  W  K  N  R  R  Q  R  K  T  T  H  R  Y  S  V  R
GATGCAAGGAAATCCCAAATCTCCACCTCTGATTCAGAAGGCAATTCAGATGAGAAGACT
 D  A  R  K  S  Q  I  S  T  S  D  S  E  G  N  S  D  E  K  T
AGCGCTATGATAAGGCACAGGAGACCGCAGCTTCTGCAGCCTTTTCCAGCAAACCAGTCT
 S  A  M  I  R  H  R  R  P  Q  L  L  Q  P  F  P  A  N  Q  S
AAAGAGCAATCTGTAACAAGTGGATGTAACTTTCTAGAATCTGAAATGGTCCAGAGCTGT
 K  E  Q  S  V  T  S  G  C  N  F  L  E  S  E  M  V  Q  S  C
AATTATGGAAGGGAGAACTCTCAACAGTTCATTCCAGCTCAAGAAAATATTGTTCAGGCT
 N  Y  G  R  E  N  S  Q  Q  F  I  P  A  Q  E  N  I  V  Q  A
TCAGATGAGTCAGTTACACCATCTGCCAGTTATCTAGACAACTCTCCTTTTGATTTGTGT
 S  D  E  S  V  T  P  S  A  S  Y  L  D  N  S  P  F  D  L  C
CATGTCTTACTGTCCTTGCTGGAAAAAATATGTAAGTTTGACATTGCATTGAACCACAAG
 H  V  L  L  S  L  L  E  K  I  C  K  F  D  I  A  L  N  H  K
TCTGCATTGGCAGCCAGTGTGATAGGCACTCTAACTGAATTCTTGTCTAAACTTGGAGAT
 S  A  L  A  A  S  V  I  G  T  L  T  E  F  L  S  K  L  G  D
TGTTACAATATAAATAGTGCTGCTGAAAATGAAGCTGTTTCATCAAGTTGGACAGAAGAA
```

C Y N I N S A A E N E A V S S S W T E E
TCAGTTGCTCTGGTTCAGAGAATGCTCTTTAGGACGGTGCTGCATCTTATGGCAGTGGAT
S V A L V Q R M L F R T V L H L M A V D
GTTAATAATGCTGATACTATGCCCGGAAATTTGAGAAAAAATCTTATTGATTTACTTAAA
V N N A D T M P G N L R K N L I D L L K
GCAGCCTTAAAAGTGAAATGTATTCTAGATGCACTATTTAGTCCTTTTTCTTCAAGATCA
A A L K V K C I L D A L F S P F S S R S
CAAGGAACTTTACAAAACGTATTGCAGGTTAATCTTTTCTCACAGAATCGCCACAGATTC
Q G T L Q N V L Q V N L F S Q N R H R F
CTTCTTTTGCCCGAGTTCATAGAAGGAGTTCTGCAAATTCTAATCTGCTGCCTTCAGAGT
L L L P E F I E G V L Q I L I C C L Q S
GCAGCCTCTAATCCAATTTATTTTAGCCAAGCAATGGATCTGGTTCACGAATTCATACAG
A A S N P I Y F S Q A M D L V H E F I Q
CAGCAGGGTTTCAAACTGTTTGAAACCACAGCACTCCAGATGGAAGGATTATGCGCCAGA
Q Q G F K L F E T T A L Q M E G L C A R
GGCCCAGAGGTCAACACAGAAGCGTCAGAGTATCTCAAAGCCCTCATCAACAGCATTATG
G P E V N T E A S E Y L K A L I N S I M
AAAATAATCAGCACTATCAAAAAAGTGAAATCGGAACAACTTCATCAGTCTGTATGTACG
K I I S T I K K V K S E Q L H Q S V C T
AGAAAACGGCACAGGCGATGTGAGTATTCTCACTTCATGCATCACCACAGAGATCTCTCA
R K R H R R C E Y S H F M H H H R D L S
GGATTGCCAGTTTCTACTTTTAAAAATCAAGCTTCTAAGAGTCCTTTTGAAGAAACAGCT
G L P V S T F K N Q A S K S P F E E T A
GATGGGGAAGTTCATTATCCTGACCGGTGTTGCTGCGTTGCTGTTTGTGCTCACCAGTGT
D G E V H Y P D R C C C V A V C A H Q C
CTGAGGTTGCTGCAAAAACTTTCTCTGAACAGTACATGTTTTCAGATCTTAACTGGAATC
L R L L Q K L S L N S T C F Q I L T G I
CATAATGTAGGCATATGTTGTTGCATGGATCCTAAATCAGTAATTAGCCCCTTGCTTCAT
H N V G I C C C M D P K S V I S P L L H
TCCTTCAGATCTCAAACATTCAAGAATTTTCAGCCACATATATTAAGCATCCTTAATAAG
S F R S Q T F K N F Q P H I L S I L N K
TTTATTTTAGAACAACTTGGAGGATTACAAATTTCTCAGAGAGTGACACATGCCTCTTGC
F I L E Q L G G L Q I S Q R V T H A S C
AACATCTGTAGCATTGACTGTGATCAACTTGCAGAACTTGATGACTTCCTACATGGGAAT
N I C S I D C D Q L A E L D D F L H G N
GCTGCTGAAGTATCAGTGTCAAGTTCATCAGCTCCATACAGGTTTCAAGGAATTTTGCCT
A A E V S V S S S S A P Y R F Q G I L P
AACAGAGGATCTGAAAATATGTTTCTGAAATGGGATGCCTTGGAGGCATATCAGAGCCTT
N R G S E N M F L K W D A L E A Y Q S L

```
GTTTTTGAGGATGATGACAAACTGCGTTGCATGCAGATTGCTAGTCATATTTGCAGTTTG
 V  F  E  D  D  D  K  L  R  C  M  Q  I  A  S  H  I  C  S  L
ATCCAAAAAGGCAATGAGATTATTCAGTGGAAACTATACAATTATATATTCAGCCCAGTG
 I  Q  K  G  N  E  I  I  Q  W  K  L  Y  N  Y  I  F  S  P  V
CTCCAGAGAGGTGTTGAGTTGGCTCATTACTCCCAGCAAGCTGGTGCAACTACTGCTTTT
 L  Q  R  G  V  E  L  A  H  Y  S  Q  Q  A  G  A  T  T  A  F
AGTCAAACAAGTGGCTATCAAAAAAGATGTCTGCCTCAGGAAGTCCTTCAGATCTATTTA
 S  Q  T  S  G  Y  Q  K  R  C  L  P  Q  E  V  L  Q  I  Y  L
CAGACTCTACCAATACTGTTTAAATCCAGAATAATACAAGAATTATTTTTAAGCTGTAAT
 Q  T  L  P  I  L  F  K  S  R  I  I  Q  E  L  F  L  S  C  N
GGAATAAATCAAATAACTGAATTAAATTACTTGGACAGTGTGAGAGCATACTCATTGAAA
 G  I  N  Q  I  T  E  L  N  Y  L  D  S  V  R  A  Y  S  L  K
GTATTTGAAACATTAATATTTTCCCTTGGAGATCAGCAGACAGATCAATTGATGCCAGAT
 V  F  E  T  L  I  F  S  L  G  D  Q  Q  T  D  Q  L  M  P  D
GTGGATGGTTTGGACAGTGAAGAAAAAGTGCCTGCCTTAGACTTGGATGTTTCTGTTCAC
 V  D  G  L  D  S  E  E  K  V  P  A  L  D  L  D  V  S  V  H
AGACAGCAAATGGTTTCCGATGTGCCTCAGAGCCTAAGTAAATTTTATGCCGGGCTCAAA
 R  Q  Q  M  V  S  D  V  P  Q  S  L  S  K  F  Y  A  G  L  K
GAAGTGAATCCGAAAAGGAAGAAATCAGTCAGTCAAGATGTTCACCTCAATATGATAAAT
 E  V  N  P  K  R  K  K  S  V  S  Q  D  V  H  L  N  M  I  N
CTATTTCTCTGTGTTACTTTTTTATGCGTAAGTAAGGAAGCAGAGGCTGACCGAGATTCA
 L  F  L  C  V  T  F  L  C  V  S  K  E  A  E  A  D  R  D  S
ACAAATGATTCTGAAGATACTTCAGGTTATGATAGTACTGCTAGTGAACCTTTCAGCCAT
 T  N  D  S  E  D  T  S  G  Y  D  S  T  A  S  E  P  F  S  H
AAGCTTCCATGTCTGTCATTTGAAAGTTTGACTCTACCATCCTTGGAACATATACACAGG
 K  L  P  C  L  S  F  E  S  L  T  L  P  S  L  E  H  I  H  R
GCAGCAGATACTTGGTCAATGTGTCGTTCGATCTATTTGTGTAATTCAATATTCCAAAGA
 A  A  D  T  W  S  M  C  R  S  I  Y  L  C  N  S  I  F  Q  R
CAATTCCATAGACTTGGCGGTTTTGAGGTATGTCACAGATTAATAGCCATGATTATTGAG
 Q  F  H  R  L  G  G  F  E  V  C  H  R  L  I  A  M  I  I  E
AAACTTGGCGTGAAAGAAGAAAAGGAAGAAGGTATGCAGGAGAAACCTACTGAATTTCTT
 K  L  G  V  K  E  E  K  E  E  G  M  Q  E  K  P  T  E  F  L
TTCAAAAATGGCTCATCGCAGTTGATAATAAACAATGGGAAAGGGCAGTCGGACAATAAT
 F  K  N  G  S  S  Q  L  I  I  N  N  G  K  G  Q  S  D  N  N
ACTGATGGAAATCTTAACAATATAGATCAACAGAAATCAATCTCTTATCAGCATTCAGAT
 T  D  G  N  L  N  N  I  D  Q  Q  K  S  I  S  Y  Q  H  S  D
GCAGAGCAAGCTTTGACTGATGGCACTGCAAAATCAAAACATGTCTTAATGCAAGAAAAT
 A  E  Q  A  L  T  D  G  T  A  K  S  K  H  V  L  M  Q  E  N
GACTGGTCGCTGCAAGGTATAAGACTCTTGGAAGCTCTGCTGGCAATTTGTTTACACAGT
```

D W S L Q G I R L L E A L L A I C L H S
GCAACTAATTTCCAGCAGAAGGCTGAAATGGAGTCTCTTCAGAACATTTGTGTGGAAGAT
A T N F Q Q K A E M E S L Q N I C V E D
ATATTACTTGAAATGAGGGAACAGCTTACTAAAGCTAGAGTAATGGAAACAGAGTTGGTG
I L L E M R E Q L T K A R V M E T E L V
AAGCCTTTATTTGATTCTTTGCTTCGAATTGCATTGGGAACATATTTTACTGATCTGGAT
K P L F D S L L R I A L G T Y F T D L D
CGTTCAGAAGAAAAGAAAGAAAAGACCCATCAGTCTGAAAAGGAGCCTGTGTCTCGGCCT
R S E E K K E K T H Q S E K E P V S R P
GATGAAATATCTGAAGAAGCTGATGAATCACAATGCTATTGCTTTAAATTTTTTGCTGAA
D E I S E E A D E S Q C Y C F K F F A E
GAAAAAGGCTATGATGCTGATAGTGAAAGTAATTCAGATGACAATGAAAGCAAGGATGAA
E K G Y D A D S E S N S D D N E S K D E
GGAACAGACATTAAAAGAGAGCAAGTATGTTTTGGCTATTGCAGTGTTCCTAGTGATCTT
G T D I K R E Q V C F G Y C S V P S D L
GCTGAAAATATTATTCAAGGAGAATTGATATATCCTGAACTATGTATGCTGGAATTACAT
A E N I I Q G E L I Y P E L C M L E L H
TTACTTTCCGTTGGCAGTCCGAGCCTAGATGTGCTCACTCATGTTTTCCACAGCTTCTTG
L L S V G S P S L D V L T H V F H S F L
AAAGTAGTCAGGCTGAAAGAAAAAAATGCAACATTACTAATGGAACAGGGAGCTGTAAAG
K V V R L K E K N A T L L M E Q G A V K
AGCCTTCTGGGAGGATTCCTCCATGTATTGACACAAACAGATTCCTGTTTTCATGAATGT
S L L G G F L H V L T Q T D S C F H E C
CAGAAAATATTAGTGGATCTGCTGGTTTCTCTGATGAGCTCAAGCACATCTTCAGAAGAG
Q K I L V D L L V S L M S S S T S S E E
CTCACACTTCTTCTAAGGATCTTTTTAGAAAAAACACCTGCTACAGGTATTCTCCTTAAA
L T L L L R I F L E K T P A T G I L L K
GGAATCCTAAAGATTTTGGAGGCAAATACCAGGATGAGGCCTTTACAGCATATCGTATTT
G I L K I L E A N T R M R P L Q H I V F
CCTTTGCGCCAAATTCCAAGTTTAGGAAGCAGCCCTTGCCAAAAATCAGCCCCTACATTC
P L R Q I P S L G S S P C Q K S A P T F
GGCAGCAAAACTGTAGCATTACTAAGGCGAGCAAAATTAGTCCAGTCAAAGAAAGAACCA
G S K T V A L L R R A K L V Q S K K E P
GAGGGTGAGAGTTTGCCACACCATCTTCTCTCTCCATGGCATGTGGCTCCAATCCATTTA
E G E S L P H H L L S P W H V A P I H L
CCTCTGGTGGGACAAAACTGCTGGCCACATATGTCTGAAGGATTCAGTATCTCGTTGTGG
P L V G Q N C W P H M S E G F S I S L W
TTTAGTGCTGAATGTATTCTCAGAGGAAGCAGTTCCTTGGAGAAAGGCAAAAAAATGAAA
F S A E C I L R G S S S L E K G K K M K

```
AGAAGGAGCAAGTCACTAATATCACATGAAAGCAGTTTTGATGAAGCAGAAGAAAGCAAA
 R  R  S  K  S  L  I  S  H  E  S  S  F  D  E  A  E  E  S  K
CCTGGACTTGATTACCTCAATTTGGGAGACAAACTTCTTGAAGATGGCAACCTTCATATA
 P  G  L  D  Y  L  N  L  G  D  K  L  L  E  D  G  N  L  H  I
TTTTCACTGGGCTCCAAAGCATTGATGATACAAGTCTGGGCTAATGTGAACTCAGGAGCT
 F  S  L  G  S  K  A  L  M  I  Q  V  W  A  N  V  N  S  G  A
TTCACATTTCGTATGTGTATGGATCCAAATGATGAAATGAAAGCTGGATTATTAGCACAA
 F  T  F  R  M  C  M  D  P  N  D  E  M  K  A  G  L  L  A  Q
GCTGAAACACCAGAGAATACCTTCCTTGTAGGCAAATGGCAGCATTTGGCCCTAACCTAC
 A  E  T  P  E  N  T  F  L  V  G  K  W  Q  H  L  A  L  T  Y
ACTGAACAACCAGAAAACAAGAAAAATATCCATGGAACTCTCTCTTTATGGATTTGTGGT
 T  E  Q  P  E  N  K  K  N  I  H  G  T  L  S  L  W  I  C  G
CAGAGAAAACCTGACATTTGTTTAGATTATACACTTCCAAGAAAAATGAGTTTGTCATCC
 Q  R  K  P  D  I  C  L  D  Y  T  L  P  R  K  M  S  L  S  S
GACTCTAATAAGACCTTCTGCATGACAGGCCATTATACATCATCCCAAGATGATTTTTTG
 D  S  N  K  T  F  C  M  T  G  H  Y  T  S  S  Q  D  D  F  L
CAATTGACTGGGAGGTGGAGCCTTGGAAATTTGCTTCTCTTCAATGGTGCAACAGTGGGT
 Q  L  T  G  R  W  S  L  G  N  L  L  L  F  N  G  A  T  V  G
CCTGAAGAGGCATTTTATCTGTATGCTTGTGGGCCAGATTTCACGTCTGTAATGCCATGT
 P  E  E  A  F  Y  L  Y  A  C  G  P  D  F  T  S  V  M  P  C
AAATATGGCAAGTCAATACCTAATTACTCCAAATATATAGATAAAGAGATTCTACAGTGT
 K  Y  G  K  S  I  P  N  Y  S  K  Y  I  D  K  E  I  L  Q  C
GAACAAATCAAAGAACTTCTGTTGACAAAAAAAGAAGTAGACACTGGGCCTTTAATTGAA
 E  Q  I  K  E  L  L  L  T  K  K  E  V  D  T  G  P  L  I  E
AACCTTGCTACAGTATACACCACTTGCTGCCCTGGTTACTACACAATCTATGAGCCAGTA
 N  L  A  T  V  Y  T  T  C  C  P  G  Y  Y  T  I  Y  E  P  V
ATCAGACTTAAAGGTCAAGCGAAAACCATTCCTTCTCAACGACCTTTCAGTTCAAAAGAA
 I  R  L  K  G  Q  A  K  T  I  P  S  Q  R  P  F  S  S  K  E
GTACAAAGCAATTTATCAGATCCTCATTATCTGAAAATGCTGCAGCCTGCCAAATACAAA
 V  Q  S  N  L  S  D  P  H  Y  L  K  M  L  Q  P  A  K  Y  K
GGTCTCCAAGGCATTCTTCATGAGATTGGTGGAATTGGGTCATTTGTCTTCCTTTTTGCC
 G  L  Q  G  I  L  H  E  I  G  G  I  G  S  F  V  F  L  F  A
AGAGTTGTAGAGATCAGCACTTGTGAAGAAACACAAGCTTTAGCTCTGAAAATTATCCTT
 R  V  V  E  I  S  T  C  E  E  T  Q  A  L  A  L  K  I  I  L
GTTTTAACAAAACACAATCAGCAGAGAATACATGAGTTAGAAAACTGCAATGGTTATTCA
 V  L  T  K  H  N  Q  Q  R  I  H  E  L  E  N  C  N  G  Y  S
ATGATTCATCAGGTGTTGATAAAGTCAAAGTGTATTGTTGGATTTCACATTTTGCAGACT
 M  I  H  Q  V  L  I  K  S  K  C  I  V  G  F  H  I  L  Q  T
CTTCTCGAAGGATGCTGTAATGAGAAAATGATTCATCAAGATGACAATGGACAATTCATG
```

L L E G C C N E K M I H Q D D N G Q F M
CTTGATGTGGAATCCAGAGCAATAATCCAGGATGTAAAATTGTTGGAAGAGCTACTGTTT
L D V E S R A I I Q D V K L L E E L L F
GATTGGAAGATATGGTCTAAAGCCCAGCAAGGTGTGTGGAAAACACTGTTATCAGCTTTA
D W K I W S K A Q Q G V W K T L L S A L
GAAACACTTATCCAAGATAATCACTGGCAACAGCAATTTAACATTAAACAACTTCTTAAA
E T L I Q D N H W Q Q Q F N I K Q L L K
GCTCGAGTTGTGCATCATTTCTTGAGGACATGTCAAGTTCTGCAGGAGCACAAAGAAGAA
A R V V H H F L R T C Q V L Q E H K E E
CATTTCACATCTATTCCTTCAGAAGTTTCTACTTCATTTGTGAAAATAATTGAAGAAGTG
H F T S I P S E V S T S F V K I I E E V
CTTGGATCTCCACCAGATCTGGAACTGCTGACCCTGATCTTCAATTTCCTTGTAGCAGTT
L G S P P D L E L L T L I F N F L V A V
CACCCTGCTACTAATACATATGTTTGTCACAATCCCAGAGATTTCTATTTTTCCTTGCAT
H P A T N T Y V C H N P R D F Y F S L H
ATAGATGGCAATATTTTTCAAGAGAAGGTTCAATCCATCATGTATATGAGAAATTCCAGT
I D G N I F Q E K V Q S I M Y M R N S S
AGTGGTGGAAAATCTGTTGACAGTCCAGTTTTAGCTATGATGAGCCCAGCTAGATTTTCT
S G G K S V D S P V L A M M S P A R F S
GCTGCCCAGCAAGGAAATGATTATTCCGAAGCAAGCAACTCTCAAAACACAGTTCATCAA
A A Q Q G N D Y S E A S N S Q N T V H Q
GCACCCGAAAGGCTCTGCAAAAGCTTACCATCATCTCCTATATCGTCCCCAGGAATGCAT
A P E R L C K S L P S S P I S S P G M H
CGGGGTTTTTTTAGGAATATAGGCTGGAGTGTTGAGAATCTTCATGCAGTTACACAGCTT
R G F F R N I G W S V E N L H A V T Q L
AAAAGAGTTGAGCCACTCCAGAAAAACAATTTGATAAAGCATACAGAAGCCTTCAAAAGG
K R V E P L Q K N N L I K H T E A F K R
AATGAGGAAGAACATTTCATTAGTAGTTGTGAATCTGCAAAAACAATTGTAGGATTGGGA
N E E E H F I S S C E S A K T I V G L G
GAAGTAATCCCTGCAGAAACACTGGGTATTTCAAGAGAAGAGAGAGAATCATTAGATAAA
E V I P A E T L G I S R E E R E S L D K
ACCTCTGAGAATCATGAAACAGTCCCAGGAGTGAAACCCAGTGAATATAATGTTATTCCT
T S E N H E T V P G V K P S E Y N V I P
GAGTTATCTCTGAAGCGGCCTGATAATCTGAAGGGGTTGTCTCCTATACAGAAAAGCCAT
E L S L K R P D N L K G L S P I Q K S H
GGCAATTTTGCAGGCTTGGGGTTAGCCTTCTCCATTCACACTGGAGCTACTGTACATCGC
G N F A G L G L A F S I H T G A T V H R
TGGCCAAGCTATAAGAATGTTCCTTCAGAAGATTGGGAACATCTCACCTTGTCTTCAGCT
W P S Y K N V P S E D W E H L T L S S A

AACGAATGGGCTCCACAAAAATTTGAAAGCATGTCTAATAGATCAACAGAAGATTGTCTC
N E W A P Q K F E S M S N R S T E D C L
GTACTCATATGCCATGGTCTTTATCATCTTCTGCGAAGAATTCTTTTAATTTTGCCAGAT
V L I C H G L Y H L L R R I L L I L P D
GTGATGCTCCCAGATGTATTGGACAAGCTGATTCAGCCAGACATTCTCATAGTTCTTGTG
V M L P D V L D K L I Q P D I L I V L V
AACCATCCATCTACTCTCATTCAGCAAGGAGTCATTAAACTTTTGAACACCTATTTCAGC
N H P S T L I Q Q G V I K L L N T Y F S
AGAGCAACTAGAGAACAAAAAGAGAAGTTTTTGAAGAACCGAGGTTTCTCCTTGTTAGCT
R A T R E Q K E K F L K N R G F S L L A
AATCAGCTGTATCTTCACCAAGGAACACAGGAAGTTATGGAATGCTTTCTAGAAATGCTT
N Q L Y L H Q G T Q E V M E C F L E M L
TTTGGTCGATCGGTTGGCCTGGATGAAGACTTTGATCTAGAAGATGTGAAAAATGCTGGG
F G R S V G L D E D F D L E D V K N A G
CATTTTCAAAAGTGGTGTGTCATTCCTGTTCTGGGCCTTATTGAGAATTCCCTCCATGAT
H F Q K W C V I P V L G L I E N S L H D
TGCATCCTGTTACATAACTCCTTTTGTTTCCTGCTGCAAATCATAAATTCTTGTCCTAAA
C I L L H N S F C F L L Q I I N S C P K
ATGGCAGACATGCTGTTGGATAATGGCTTGTTGTATGTGCTCTGTAATACTTTAGCAACA
M A D M L L D N G L L Y V L C N T L A T
CTTAATGGACTTGAAGCCAACATTCTCTCAAATGACTCCAGATTACTTGTGTATGATATT
L N G L E A N I L S N D S R L L V Y D I
CAGCAGTTACTTGCAGCAGTGACAATCCATGCTTGCAGCTGTTCAGGAACACAATATTTT
Q Q L L A A V T I H A C S C S G T Q Y F
CGGATTATTGAAGATCTTATGGTGTTACTTGGACATCTGCAAATGAGCAAGAATGACAGA
R I I E D L M V L L G H L Q M S K N D R
ACACAGAAAATGGCAGTTAATTTGCAATTCACAGTCTTGCAATCAGCTGTAAAGTATATA
T Q K M A V N L Q F T V L Q S A V K Y I
AAAACTGTAGGAAATCCAGATTCAGAGCATTTAAGCCATTCACTCCCTTTATCATTTGAA
K T V G N P D S E H L S H S L P L S F E
GATCATCATGCTATATTTCAAAAACATAAAAGCATCGCTGCACCAAGAAAATTTTCTATT
D H H A I F Q K H K S I A A P R K F S I
GCTCAGCCTGAGACATTCCTAATGAAAATGCATACAATTGCAAACGAGGCACTGGATTTT
A Q P E T F L M K M H T I A N E A L D F
ATGATGCTGCGTAGAATGAATCAAGACAGTTCTCTTTGTGCCACTGAAGCAGAGCTTATA
M M L R R M N Q D S S L C A T E A E L I
CAGAGGTTACAGAGGATTACTGTACTAACCACCAATAGATTCATGTATCAAGACCTTTCT
Q R L Q R I T V L T T N R F M Y Q D L S
GAAAATTGTCCTGAAATGATTGAGATCCCATTCCAGAATAGATCTTCCAGAATAGTTTCC

```
 E  N  C  P  E  M  I  E  I  P  F  Q  N  R  S  S  R  I  V  S
CAACAGGACAAGACTGAAAAGGAGGATGAACAGAAAGAACTTATCCCAAGAAACACTTTC
 Q  Q  D  K  T  E  K  E  D  E  Q  K  E  L  I  P  R  N  T  F
TTGAGAGAAATGTTTAAAATAATGATTGAAGGAATCGGATTATCTATTGGCACTTCCAGA
 L  R  E  M  F  K  I  M  I  E  G  I  G  L  S  I  G  T  S  R
TTAAGCACCCCCAAGCAACAGTGGAAGAAAATTCTCTTGTCTTGTAAGGATACTTTTCGA
 L  S  T  P  K  Q  Q  W  K  K  I  L  L  S  C  K  D  T  F  R
GTGCAGCTTGGGCGACTGCTTGTGCATGCTTTGTCTCCAACACGCTCTTTGCAAGAACGA
 V  Q  L  G  R  L  L  V  H  A  L  S  P  T  R  S  L  Q  E  R
AAGCACACTTTGGAATTCTTACATGAAATTAACCACCAGGAGATTTTGCGAGAGTGTTTA
 K  H  T  L  E  F  L  H  E  I  N  H  Q  E  I  L  R  E  C  L
AGCACAACTTTGCAGCATGGGCCTAAGTTGTCACTGTATTTATTTGAATTAATGCATGAT
 S  T  T  L  Q  H  G  P  K  L  S  L  Y  L  F  E  L  M  H  D
CATAAAGATGAATTAACCAAAGAAGAACAGCTATCGGTCGGAGCATTCATGAACACATTA
 H  K  D  E  L  T  K  E  E  Q  L  S  V  G  A  F  M  N  T  L
AAGCTTTGTGGGTACAAATACATTCCTCTCAATGCTCCACCGAAACCTGACCTCATAAAA
 K  L  C  G  Y  K  Y  I  P  L  N  A  P  P  K  P  D  L  I  K
GCAATGGAAGAGGACCATAAGAAATACCTGGGCGAAGAAGAGGTGAATAAAGCTGCTTGG
 A  M  E  E  D  H  K  K  Y  L  G  E  E  E  V  N  K  A  A  W
GAAAAGATAATGGCCAACAACTGGCAAACTCTATTTCAGCGCTTGGATGCAAAGTCAAAG
 E  K  I  M  A  N  N  W  Q  T  L  F  Q  R  L  D  A  K  S  K
GAGATTTCTAAAATTGCAGGGGACATCACTCAGGCTGCCTCTCTGTCCCAGGGAATGGAA
 E  I  S  K  I  A  G  D  I  T  Q  A  A  S  L  S  Q  G  M  E
AGAAAGAAGGTGATTCAGCACATCAGGGGGATGTATAAAACAGACTTAAGTGCCAGCAGA
 R  K  K  V  I  Q  H  I  R  G  M  Y  K  T  D  L  S  A  S  R
CACTGGCAGGAGCTCATTCAGCAGTTTACACATGATAGGGCTCTTTGGTATGACCCTGCC
 H  W  Q  E  L  I  Q  Q  F  T  H  D  R  A  L  W  Y  D  P  A
TCCTACCCAACATCTTGGCAGCTGGACCCAACCGAAGGACCTAACAGAGAGAGACGCCGC
 S  Y  P  T  S  W  Q  L  D  P  T  E  G  P  N  R  E  R  R  R
TTGCAGAGGTGCTACTTAACAATTCCAAATAAATACCTCCTCAAAGACAGGCAAAAATCG
 L  Q  R  C  Y  L  T  I  P  N  K  Y  L  L  K  D  R  Q  K  S
GAAGAGGTTCTTAAGCCCCCTCTGGCCTATCTGTTTGAAGACAAAACACATTCATCTCAC
 E  E  V  L  K  P  P  L  A  Y  L  F  E  D  K  T  H  S  S  H
TCTTCTACAGTGAAAGACAAAGCTGCCAGTGAGCATATAAGAGTTAATCGAAGATGTATA
 S  S  T  V  K  D  K  A  A  S  E  H  I  R  V  N  R  R  C  I
AATGTGGCCCCATCTAGAGAGACCCCTGGTGAATTGTTACTGGGCAAATGTGGAATGTAT
 N  V  A  P  S  R  E  T  P  G  E  L  L  L  G  K  C  G  M  Y
TTTGTGGAAGACAATGCTTCTGAAATTATTGAAAATTCAAGTTTTCAGGGAGAAACTGAG
 F  V  E  D  N  A  S  E  I  I  E  N  S  S  F  Q  G  E  T  E
```

```
CCGGCATCATTTTCTTGGATATTTGAAGAAATCAAAGAAGTGCACAAGCGTTGGTGGCAG
 P  A  S  F  S  W  I  F  E  E  I  K  E  V  H  K  R  W  W  Q
TTAAGAGATAATGCTGTAGAAATATTTTTAACAAATGGCAGAACGTTACTATTAGCATTT
 L  R  D  N  A  V  E  I  F  L  T  N  G  R  T  L  L  L  A  F
GACAACACAAAGGTGCGGGATGATGTGTACCACAATATCCTAACAAACAATCTTCCTAAT
 D  N  T  K  V  R  D  D  V  Y  H  N  I  L  T  N  N  L  P  N
CTCTTGGAGTATGGCAACATTACTGCATTAACCCATCTGTGGTATACTGGACAGATCACT
 L  L  E  Y  G  N  I  T  A  L  T  H  L  W  Y  T  G  Q  I  T
AATTTTGAATACCTCACTCATTTAAATAAACATGCTGGCCGTTCCTTCAACGATCTCATG
 N  F  E  Y  L  T  H  L  N  K  H  A  G  R  S  F  N  D  L  M
CAGTATCCAGTATTTCCCTTTATACTTTCCGACTATACTAGTGAAACATTGGACTTAAAT
 Q  Y  P  V  F  P  F  I  L  S  D  Y  T  S  E  T  L  D  L  N
GACCCATCTGTTTATAGAAATTTGATCAAGCCAATAGCTGTATAGtcaaaagaaaaggaa
 D  P  S  V  Y  R  N  L  I  K  P  I  A  V  *
gatcgttatgtggacacttacaagtacctagaagaagaatatcgtaaaggagctagagaa
gatgatccaatgcctcctgtacaaccatatcattatggttctcattattcaaatagtgga
acagttcttcactttctggttagaatgcctcctttcacaaagatgtttttagcttatcaa
gatcagagttttgatattccagacagaactttccattctactaacacaacctggcgcctt
tcatcatacgaatccatgactgatgtaaaggaactcatcccagaatttttttatcttcct
gaatttttagttaacagggaaggttttgattttggggtacgtcagaatggtgaaagagta
aaccacgtgaacctcccaccctgggctcgaaatgatccaagattatttatcctgatccat
cgtcaagctttagagtctgaccatgtttcccaaaccatctgccattggattgatttagta
tttgggtataaacaaaaaggcaaggcctccgttcaagctattaatgtctttcatcctgct
acatattttggaatggacgtgtcagctgttgaagatccagtccaaagaagagctctggag
accatgataaaaacatatgggcagactcctcgacagctgttccacactgcccacgtcagc
agatcgaaacctatggaaggagaactccctgctgcaatgggattattagtgcagtttgcg
ttcagagaaacaagggagcatacgaaagagattgtctatccaagtcctttaccttggatc
agaggcttaaaatgggggagtacgtaggttctcccagtgcccctgatccacttgtctgc
tttagccagccacatggagaaagatttggctccttgcaggctttaccaactagagcaatt
tgtggtttgtcccgaaagttctgccttctgatgatctatagcaaagagcaaggtgttaga
agcatgcatagcacagatatccagtggtcagccatcctgagttggggatatgcagataat
attttaagattaaaaagcaagcaaagtgaacctccagtaaattttatacagagttcacag
tttcaccaggtaacaagttgtgcttgggtgccagacagctgccaactttttactggaagt
aaatgtggagttatcacagcgtatatcaacagattcactagtagcactccttctgaaatc
gacatggagtctcagatgcatctatatggacacactgcagagatcaccagcttgttcgtc
tgcaaaccctatagcatcatgataagtgtgagcaaagatggaacctgtatcatatgggat
ttgaacaggctctgctatgtgcaaagtcttgctgggcacaaaagtcctgttacggcagtg
tctgctagtgaaacaacaggggacattgcaactgtttgcgattcagttggtggtggtagt
gacctcagactctggacagtcaatggtgacctggtgggacatgtgcattgcagagaaatc
```

atatgctctgttgctttctccaaccagcctgaaggaatctctatcaatgtgattgctgga
gggctagaaaatggagttgtgagattgtggagcacatgggatttgaaacctgtccgagaa
attacattttctaaatcaaataaaccaattgtcagccttaccttttcgtgtgatggccat
catttgtacaccgcaaataacgaaggaacggtgatctcttggtgtcgcaaggaccaacag
cgcttgaagcttcctatgttctactcattcctcagcagctatgcagctggt**tga**cagctc
tgtcggcttctcagctttggtaaattatgaagcactttcagtacagagtggactgtgaag
cctctttggttttgctagattttgaagtacatgtatgactttatcatcagcaagtagacc
tttgatgacgtcaacgaatggctgctacgtatgctcatgcaccaaatttatgaaccatat
cacagtgataagaaaattctggcttgcctttttgggtaccaaaaggacactaagggcgct
aattcagaagtatatatgctgcaatatgttatgtaacataaacatatttttatggatact
ttcctggacatacatattctaaatcttcaagcattagtgataacttttgctcatctgtta
tgtaaaaatataattaaaatatttcaaaggtttatataaaaaccagtgtttgtgtttaat
tcattattagattataatccaccttgttcttctatacatataaccatctcaaat

Bibliography

1. Ullate-Agote A, Milinkovitch MC, Tzika AC: The genome sequence of the corn snake (Pantherophis guttatus), a valuable resource for EvoDevo studies in squamates. Int J Dev Biol 2014, 58:881-888.

2. Ullate-Agote A, Chan YF, Tzika AC: A Step-by-Step Guide to Assemble a Reptilian Genome. Methods Mol Biol 2017, 1650:47-67.

3. Brykczynska U, Tzika AC, Rodriguez I, Milinkovitch MC: Contrasted evolution of the vomeronasal receptor repertoires in mammals and squamate reptiles. Genome Biol Evol 2013, 5:389-401.

4. Tzika AC, Ullate-Agote A, Grbic D, Milinkovitch MC: Reptilian Transcriptomes v2.0: An Extensive Resource for Sauropsida Genomics and Transcriptomics. Genome Biol Evol 2015, 7:1827-1841.
